# Application of 18*β*-glycyrrhetinic acid Fluorescent probes in cell imaging

**DOI:** 10.1080/14756366.2026.2631869

**Published:** 2026-03-09

**Authors:** Yifan Zhao, Hongxia Sun, Zhiwei Yan, Youde Wang, Shuai Li, Yachun Guo, Guangxin Miao, Tienan Wang, Liying Zhang, Chengjun Song

**Affiliations:** ^a^ Laboratory of Traditional Chinese Medicine Research and Development of Hebei Province, Institute of Traditional Chinese Medicine, Chengde Medical University, Chengde, Hebei, China; ^b^ Department of Pathogen Biology, Chengde Medical University, Chengde, Hebei, China; ^c^ Department of Human Anatomy, Chengde Medical University, Chengde, Hebei, China

**Keywords:** 18*β*-Glycyrrhetinic acid, anti-inflammatory targets, macrophages, fluorescence probes

## Abstract

Fluorescently labelled small molecule probes (fluorescent probes) play an important role in cell imaging and are often used in combination with light-affinity probes to determine the subcellular localisation of target proteins. To investigate the target proteins of 18*β*-glycyrrhetinic acid (18*β*-GA), which regulates the macrophage inflammatory response, we designed and synthesised three types of fluorescent probes. We analysed its structure-activity relationship by evaluating the biological activity and screening for fluorescent probes with high activity. Our results showed that modifying C-3 hydroxyl and C-30 carboxyl groups enhanced the anti-inflammatory activity of 18*β*-GA, and found that two preferred probes had similar effects on LPS-induced, inflammation-related factor release (IL-1β, TNF-α, IL-6, HDAC8, P-STAT3, and SOCS3) to those of 18*β*-GA. Fluorescence signals of the preferred probes **Ia** and **IIc** were observed in the cytoplasm. The above results indicated that the anti-inflammatory site of 18*β*-GA may be located in proteins in the cytoplasm, which would provide useful information for research on the anti-inflammatory targets of 18*β*-GA.

## GRAPHICAL ABSTRACT

**Figure:**
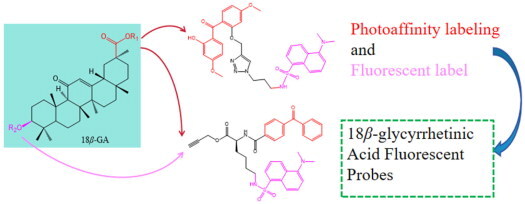

## Introduction

**Figure 1.**
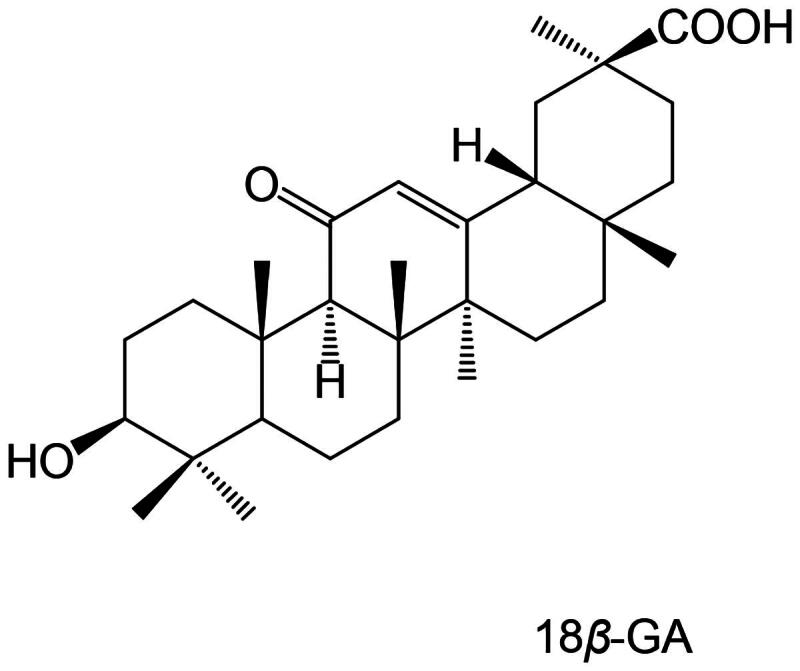
Structural diagram of 18*β*-glycyrrhetinic acid (18*β*-GA).

Inflammation is a complex immune response that serves as the body’s adaptive protective mechanism against various stimuli such as infection, injury, and allergies. The inflammation can be acute or chronic. Pneumonia is an acute infectious respiratory disease caused by one or more pathogens^1^. Chronic inflammation has a long-term impact on human health and is associated with various major vascular diseases that pose serious threats to human health^2^. Public health problems caused by inflammation can result in serious medical burdens and significant economic impacts^3^. 18*β*-Glycyrrhetinic acid (18*β*-GA, Figure 1) is a primary bioactive metabolite derived from glycyrrhizin, the main triterpenoid saponin found in the roots of liquorice (Glycyrrhiza species). Liquorice has a long history of use in traditional medicine systems worldwide for its diverse pharmacological properties, including anti-inflammatory, anti-viral, and hepatoprotective effects. 18*β*-GA itself has significant anti-inflammatory effects and great potential in the development of anti-inflammatory drugs^4^.

The mechanism of inflammation is complex and may involve multiple signalling pathways. The inflammatory response may be mediated by signalling pathways such as NF-κB, JAK/STAT, and PI3K/AKT^2^, which in turn release a large amount of inflammatory factors such as IL-1β, IL-6, and TNF-α under inflammatory conditions^5–8^. In addition, under the stimulation of inflammation, some transcription factors such as HDAC8, SOCS3, STAT3, and P-STAT3 are induced^9–12^. The anti-inflammatory effect of 18*β*-GA may be related to these cytokines^13–14^. Although some progress has been made in the study of the anti-inflammatory effects of 18*β*-GA, its target protein remains unclear; more research is needed in this area.

Modifying the C-3 hydroxyl and C-30 carboxyl groups of 18*β*-GA preserves its anti-inflammatory activity^15^. Therefore, in the present study, a series of new probe molecules with fluorescent labelling groups (18*β*-GA- PAL-ABP) were synthesised based on ABPP (Activity-Based Protein Profiling) technology using 18*β*-GA as the active group, which had different linkers. As a bridge between the active and reporter groups, the linker length changes the spatial distance and configuration freedom between the two, which affects the activity and targeting of the active group. In addition, by introducing a photoaffinity labelling group as a functional backup, when the probe molecule cannot label the target protein, covalent binding occurs by increasing the illumination conditions, thereby improving the binding stability of the probe molecule to the target protein. An inflammatory model was established by inducing macrophages with LPS, and the effects of the probes on the expression of inflammatory factors TNF-α, IL-6, IL-1β, and inflammatory pathway-related proteins HDAC8, SOCS3, STAT3, and P-STAT3 were determined. Finally, we selected probes with biological activity similar to that of 18*β*-GA and conducted cell localisation experiments to preliminarily evaluate the target location of 18*β*-GA while exerting its anti-inflammatory effects. The structure-activity relationship of the molecular structure of the probe affecting the anti-inflammatory effects of 18*β*-GA was analysed, providing a foundation for further exploring the targets of 18*β*-GA.

## Results and discussion

### Chemistry

**Scheme 1.**
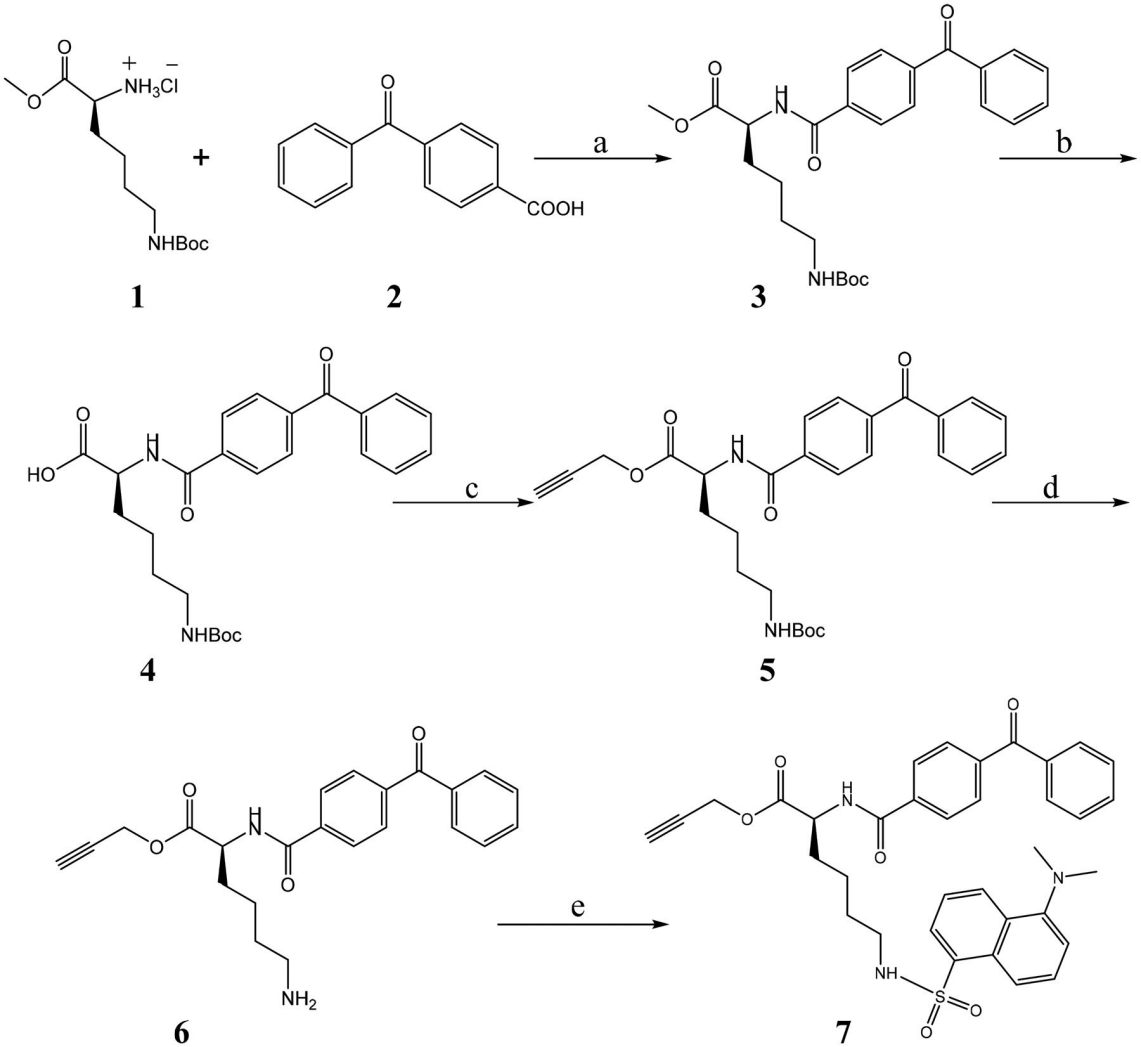
Synthetic routes of compounds **1**–**7**. Reagents and conditions: a. DMF, EDCI, DMAP, DMM, r.t., overnight, (73%); b. MeOH, NaOH, r.t., 3 h (91%); c. 3-Bromopropyne, K_2_CO_3_, DMF, r.t., 6 h (94%); d. TFA, DCM, 0 °C to r.t., 3 h (84%); e. DNS-Cl, DCM-MeOH, 0 °C to r.t., 8 h (49%).

The preparation method of intermediate **7** for the reporting group was designed according to a previously reported method (Scheme 1)^16^. Using 4-benzoylbenzoic acid and *L*-lysine methyl ester hydrochloride as reaction materials, the fluorescently labelled reporting group intermediate **7** was prepared in five steps. The specific steps were to use EDCI/DMAP to treat the reaction materials to afford compound **3**. Compound **3** was hydrolysed with NaOH to yielded carboxylic acid **4**, which was esterified with 3-Bromopropyne to form alkyne **5**. Subsequent deprotection of **5** using TFA gave amide **6**, and then amide **6** was treated with dansyl chloride (DNS-Cl) to generate intermediate **7**.

**Scheme 2.**
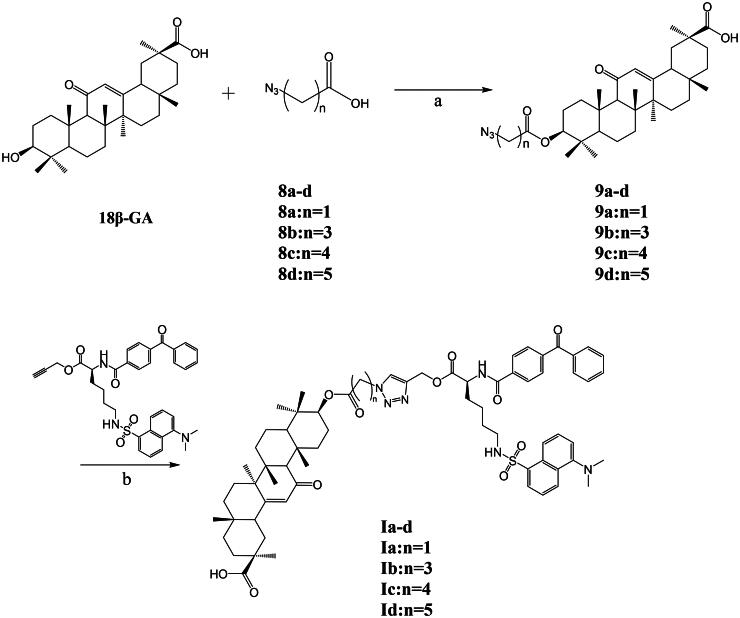
Synthetic routes of compounds **Ia**–**Id**. Reagents and conditions: a. DCM, EDCI, DMAP, 0 °C to r.t., 8 ∼ 14 h; b. THF-H_2_O, sodium ascorbate, CuSO_4_·5H_2_O, r.t., 8 ∼ 22 h.

To introduce a photoaffinity tag at the C-3 hydroxyl site of 18*β*-GA, a click chemistry strategy was employed between the 18*β*-GA and the photoaffinity tag moiety. After activating the carboxyl functional group in the azide with EDCI, a DMAP catalyst was added to form the amide intermediate, and then 18*β*-GA was added for the esterification reaction (Scheme 2). Four different lengths of linking groups, **8a**–**8d** (azidoacetic acid, 4-azidobutanoic acid, 5-azidobutanoic acid, and 6-azidoacetic acid), were esterified with 18*β*-GA to obtain the corresponding azide derivatives **9a**–**9d**. The click chemistry method mainly involves a reaction between the azide and alkynyl groups catalysed by Cu (I). First, azide derivatives **9a**–**9d** and alkynyl compound **7** were dissolved in a mixed solvent of THF-H_2_O. Then, under the catalysis of CuSO_4_·5H_2_O and sodium ascorbate, a dipole cycloaddition reaction occurred to obtain probes **Ia**–**Id**.

**Scheme 3.**
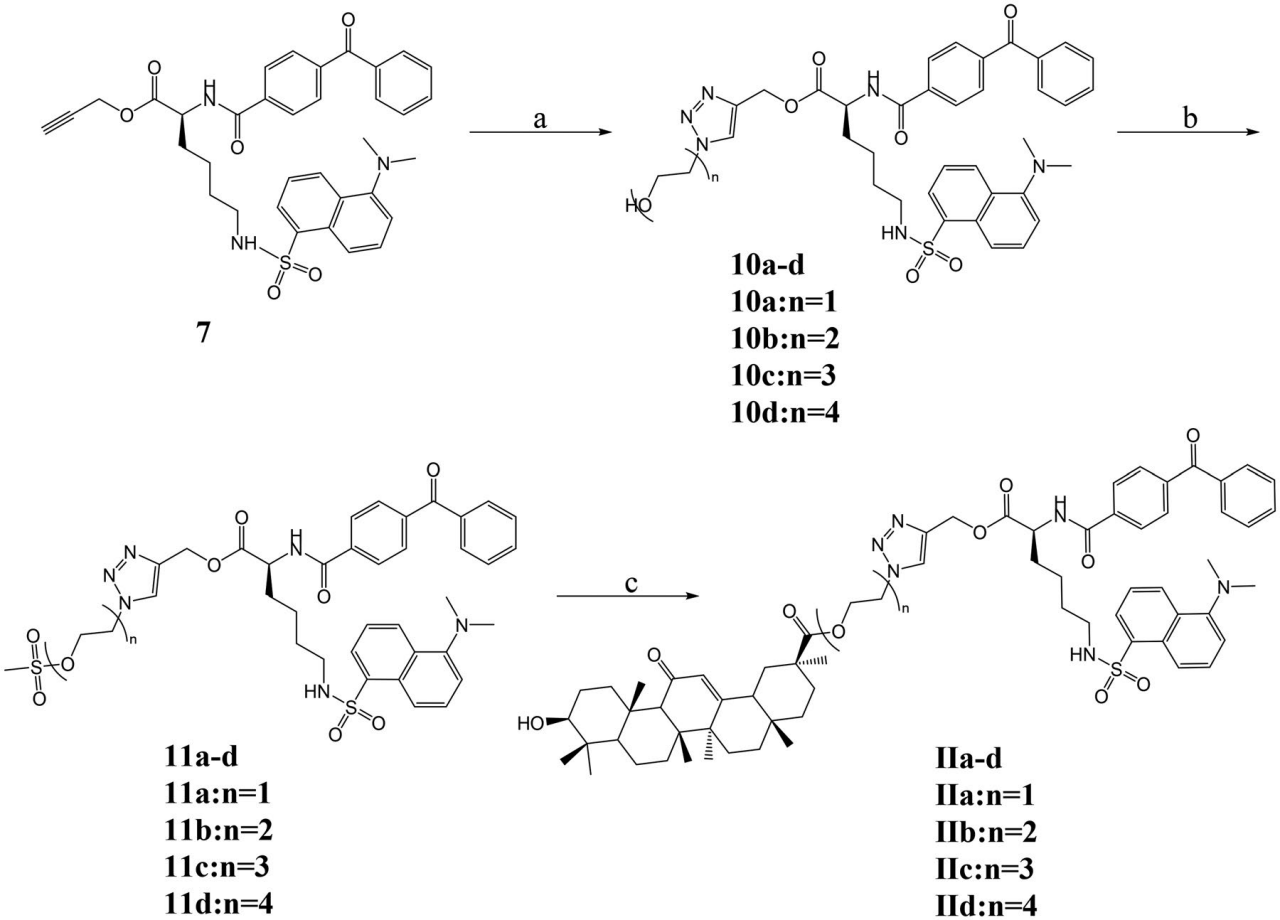
Synthetic routes of compounds **IIa**–**IId**. Reagents and conditions: a. Azido-PEG(n)-alcohol, CuSO_4_·5H_2_O, sodium ascorbate, THF-H_2_O, 30 °C, 6 h; b. Methylsulfonyl chloride, DIPEA, DCM, N_2_, 0 °C to r.t., 3 h; c.18*β*-GA, K_2_CO_3_, DMF, 75 °C, 12 h.

Compound **7** was reacted with the corresponding azides (2-azidoethanol, azido-PEG2-alcohol, azido-PEG3-alcohol, and azido-PEG4-alcohol) using the click chemistry method described in Scheme 2 to obtain the corresponding compounds **10a**–**10d**. Reacting compounds **10a**–**10d** were treated with methylsulfonyl chloride to produce their corresponding sulphonyl ester products **11a**–**11d**. **11a**–**11d** reacted with 18*β*-GA and successfully obtained the target compound probes **IIa**–**IId** (Scheme 3).

**Scheme 4.**
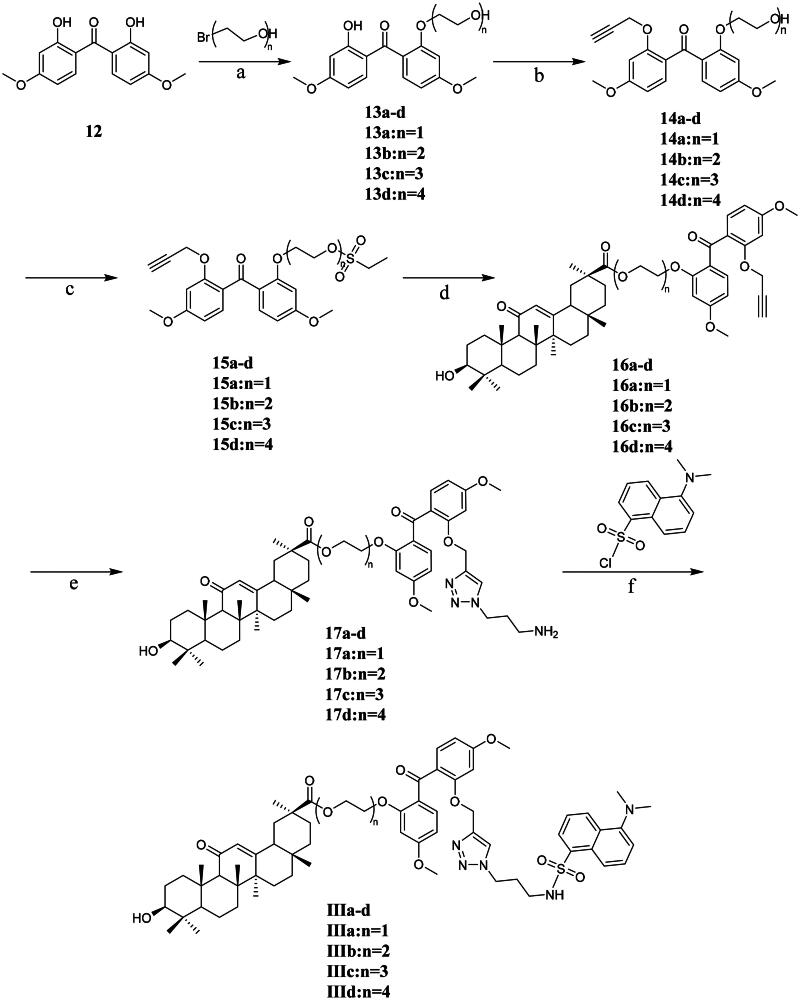
Synthetic routes of compounds **IIIa**-**IIId**. Reagents and conditions: a. t-BuOK, 18-crown-6, THF, 0 °C to r.t., 2 d; b. 3-Bromopropyne, t-BuOK, 18-crown-6, THF, r.t., overnight; c. ethyl sulphonyl chloride, DIPEA, DCM, r.t., overnight. d. 18*β*-GA, K_2_CO_3_, DMF, r.t., overnight; e. 3-azidopropan-1-amine, CuSO_4_·5H_2_O, sodium ascorbate, THF-H_2_O, r.t., overnight; f. Et_3_N, MeOH/DCM, 0 °C to r.t., overnight.

Compounds **IIIa**–**IIId** were prepared using bis (2-hydroxy-4-methoxyphenyl) ketone as the photoaffinity group **12**, as described in Scheme 4. Compound **12** was reacted with four different linking groups (2-bromoethanol,(2–(2-bromoethoxy)ethanol, 2–(2-bromoethoxy)ethoxy)ethanol, and 2–(2-(2-bromoethoxy)ethoxy)ethanol) in the presence of the phase transfer agent 18-crown-6 and potassium tert-butoxide to obtain alcohols **13a**–**13d**. The treatment of alcohols **13a**–**13d** with bromopropyne yielded alkynes **14a**–**14d**. Compounds **14a**–**14d** were reacted with ethyl sulphonyl chloride to produce the corresponding sulphonyl ester products **15a**–**15d**. Compounds **15a**–**15d** reacted with 18*β*-GA and successfully obtained intermediates **16a**–**16d**. Intermediates **16a**–**16d** reacted with their azide and alkyne groups under Cu (I) catalysis to form the triazole intermediates **17a**–**17d**. Intermediates **17a**–**17d** underwent a sulfonylation reaction with dansyl chloride to obtain the target probe molecules **IIIa**–**IIId**.

### Preliminary study on the biological activity of probe molecules

#### Effects of 18β-GA-PAL-ABP on inflammatory factors

Mouse mononuclear macrophages (RAW 264.7) were stimulated with LPS for 15 h to establish a macrophage inflammation model. ELISA and western blotting were used to determine the effects of the target compounds on macrophage inflammation.

**Figure 2.**
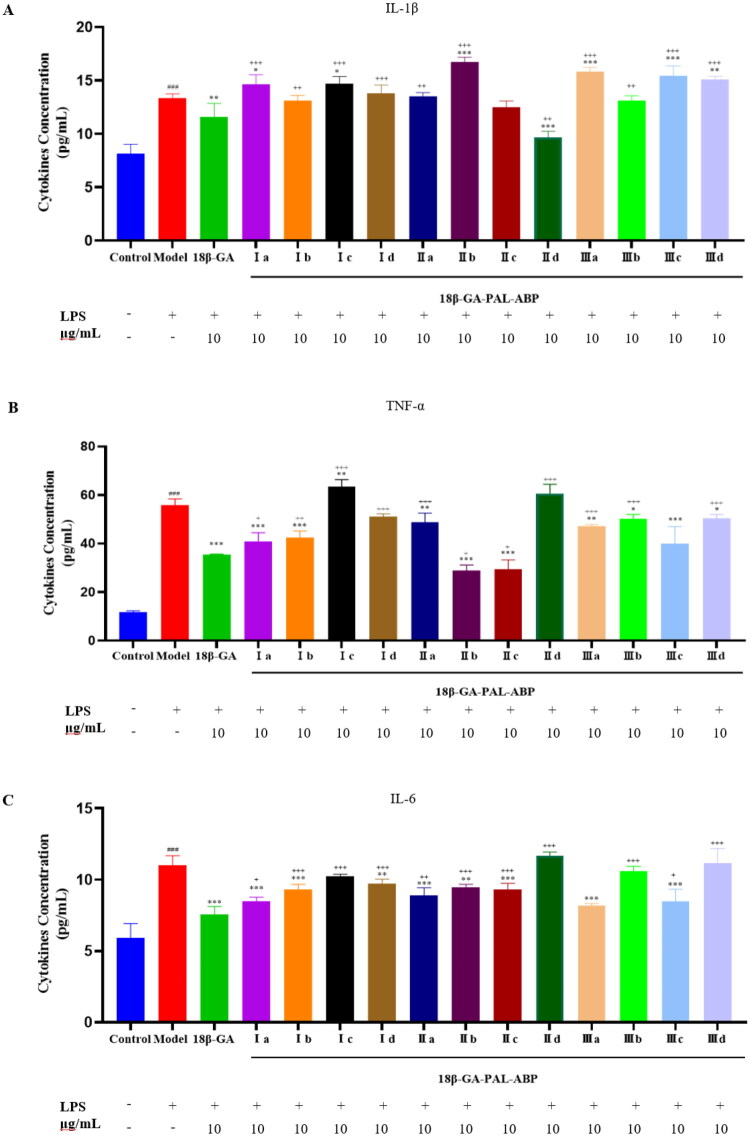
Effects of 18*β*-GA-PAL-ABP on the LPS-induced release of IL-1β, TNF-α, and IL-6 and drug concentrations. A: IL-1β content; B: TNF-α content; C: IL-6 content. Compared to the Control group, #*p* < 0.05, ##*p* < 0.01, ###*p* < 0.001. Compared to the Model group, *p < 0.05, **p < 0.01, ***p < 0.001. Compared to the 18*β*-GA group, +*p* < 0.05, ++*p* < 0.01, +++ *p* < 0.001.

IL-1β, IL-6, and TNF-α are common pro-inflammatory factors that have significant implications for the occurrence and development of inflammation^12^. IL-1β is an important pro-inflammatory cytokine that can be released through the NF-κB and JAK/STAT pathways^17^. TNF-α can synergize with IL-1β to further intensify the inflammatory response, producing a cascade amplification effect^18^. In addition, IL-6 regulates humoral and cellular immunity^19^. As shown in Figure 2, Compared with the Model group, we found that 18*β*-GA had significant inhibitory effects on IL-1β, IL-6, and TNF-α (*p <* 0.05), and **Ia**, **IIc**, and **IIIc** had significant inhibitory effects on IL-6 and TNF-α (*p* < 0.05). Except for IL-1β, most probes exhibited effects similar to those of 18*β*-GA on IL-6 and TNF-α (Figure 2.B, **C**), but in general their activity was weaker than that of 18*β*-GA. Probe molecules exhibited different activities towards inflammatory factors owing to their different linker lengths and modification sites.

**Figure 3.**
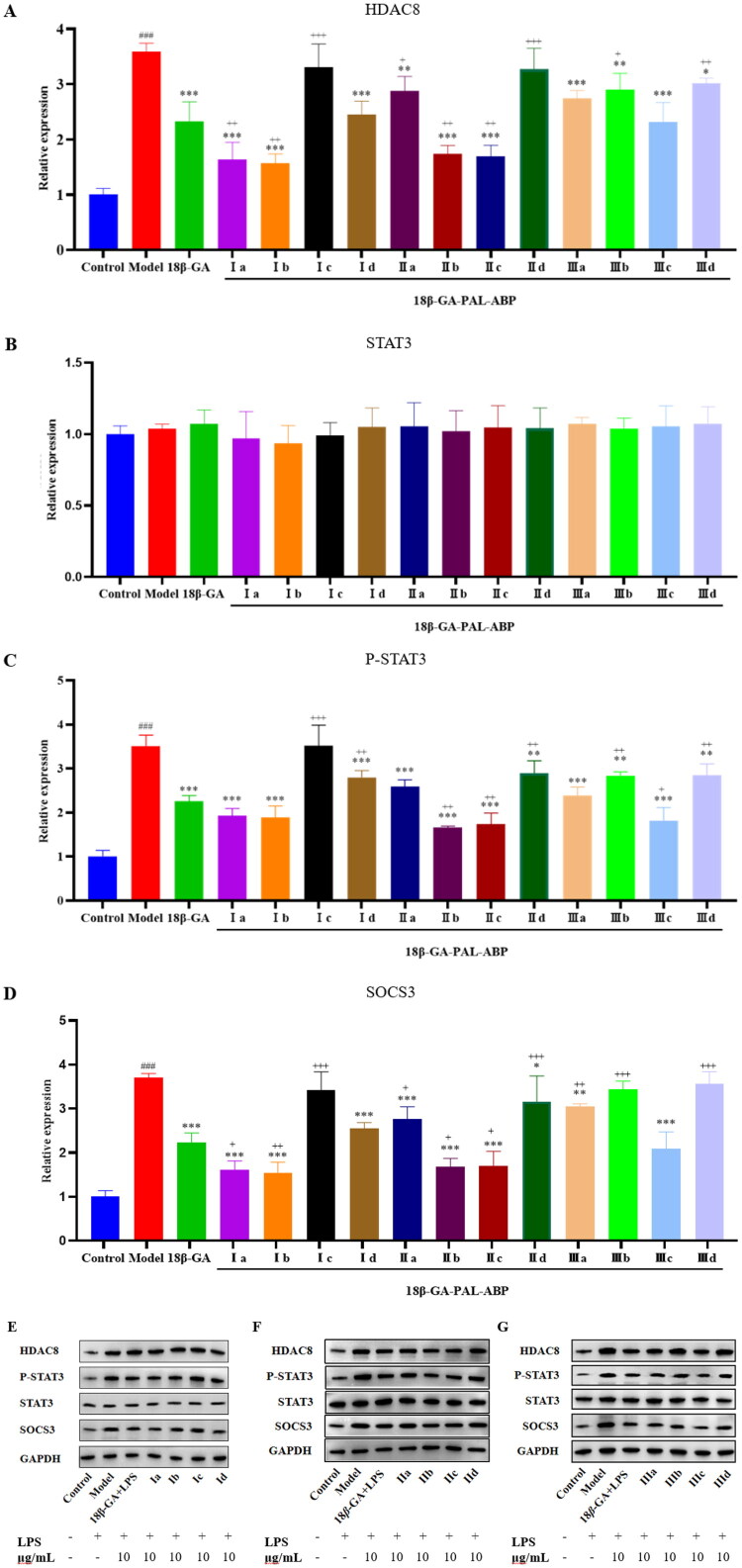
Effects of 18*β*-GA-PAL-ABP on LPS-induced release of HDAC8, STAT3, P-STAT3, and SOCS3. A: expression of HDAC8 protein; B: expression of STAT3 protein; C: expression of P-STAT3 protein; and D: expression of SOCS3 protein. E: The protein expression map in RAW 264.7 cells treated with Class **I** compounds and drug concentrations; F: The protein expression map in RAW 264.7 cells treated with Class **II** compounds and drug concentrations; G: The protein expression map in RAW 264.7 cells treated with Class **III** compounds and drug concentrations. Compared to the Control group, #*p* < 0.05, ##*p* < 0.01, ###*p* < 0.001. Compared to the Model group, *p < 0.05, **p < 0.01, ***p < 0.001. Compared to the 18β-GA group, +*p* < 0.05, ++*p* < 0.01, +++*p* < 0.001.

Although some progress has been made in the study of the anti-inflammatory effects of 18*β*-GA, the underlying mechanism and potential targets of its anti-inflammatory effects remain unclear. Therefore, for the potential target of 18*β*-GA, we spanned the cell membrane, expanding the target range from extracellular inflammatory factors to intracellular inflammatory signalling pathways. We chose HDAC8, which is closely related to the NF-κB pathway, and P-STAT3, which is involved in the regulation of the JAK/STAT, STAT3, and SOCS3 pathways. These inflammation-related signalling pathway proteins play a crucial regulatory role in inflammation. HDAC8 regulates the expression of key inflammatory factors, such as IL-1β^20–21^. STAT3 is a transcription factor induced by IL-6 that is phosphorylated to P-STAT3, allowing it to enter the nucleus and regulate the expression of downstream genes^12^^,^^22^. SOCS3 exerts anti-inflammatory effects by inhibiting IL-6-induced STAT3 phosphorylation, reducing the production of inflammatory mediators such as TNF-α and iNOS^23^. Western blotting results showed that most probes exhibited activity comparable to or even better than 18*β*-GA. Compared to the Model group, the expression levels of HDAC8, p-STAT3, and SOCS3 proteins were significantly reduced in groups **Ia**, **Ib**, **IIb**, **IIc**, and **IIIc** (*p* < 0.05) (Figure 3(A, C, D)). In addition, 18*β*-GA and the probes did not affect STAT3, and the anti-inflammatory effect of 18*β*-GA may not be directly related to STAT3 (Figure 3(B)).

#### Structure-activity relationship

**Figure 4.**
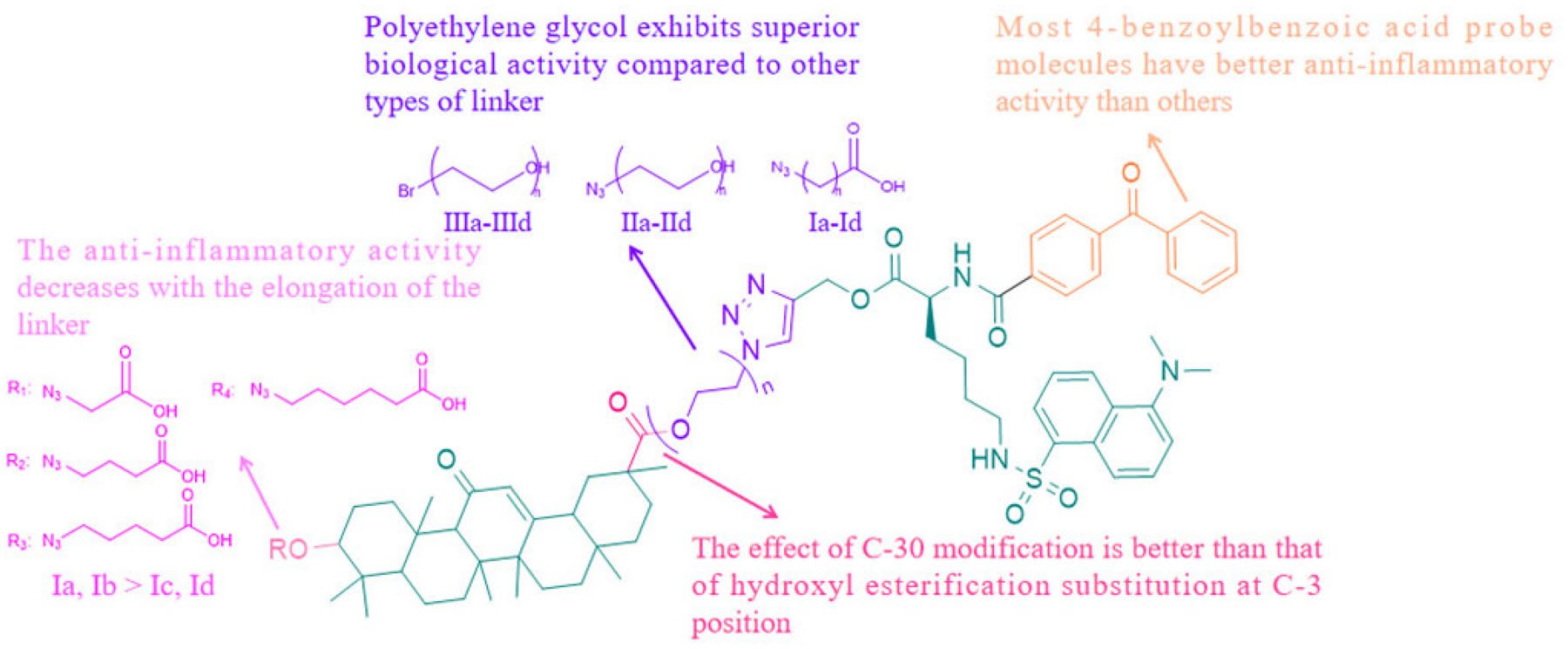
Structure-activity relationship of target probe molecules.

Based on the above results, we summarised the structure-activity relationship of 18*β*-GA (Figure 4). For different photoaffinity labelling groups, compared with 2-hydroxy-4-methoxyphenyl, most 4-benzoylbenzoic acid probe molecules had better inhibitory effects on inflammatory factors and inflammation-related proteins. In probe molecules containing 4-benzoylbenzoic acid, the inhibitory effects of inflammatory factors and inflammation-related proteins decreased with longer linkers (**Ia**, **Ib** > **Ic**, **Id**). At the same time, it was found that the effect of the C-30 modification seemed to be better than that of the hydroxyl esterification substitution at the C-3 position. When the linker was short, the anti-inflammatory activity of **IIa** was weak. However, when the linker was appropriately extended, their (**IIc**) biological activity was superior to that of the other types of linker probes (**Ic, IIIc**).

### Preliminary investigation of target proteins for active probe molecules

**Figure 5.**
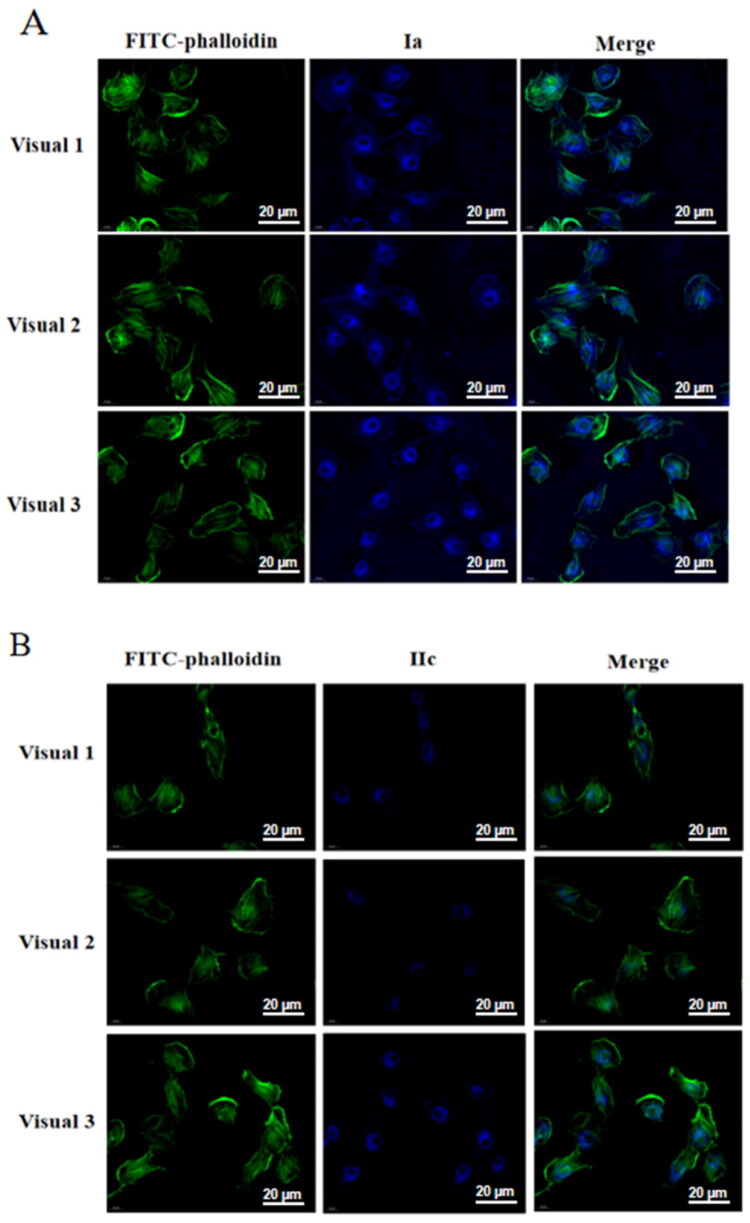
Immunofluorescence results of macrophages treated with probes **Ia** and **IIc**. Scale bar: 20 µm. **Ia**, **IIc:** UV excitation wavelength 330–380 nm, emission wavelength 420 nm (blue); FITC excitation wavelength of 495 nm and an emission wavelength of 520 nm (green).

Fluorescent probes are often used in combination with photoaffinity probes to process biological samples, achieve cellular localisation of target proteins, and directly image living cells^24–25^. Based on the activity test results of the probes, we selected probes **Ia** and **IIc** for cell localisation experiments to preliminarily explore the site of action of 18*β*-GA’s anti-inflammatory effects. The results of the cell immunofluorescence experiments (Figure 5) suggested that strong fluorescence signals were observed in the cytoplasm of **Ia** and **IIc**, further suggesting that the main target of 18*β*-GA may be a protein located in the cytoplasm.

**Figure 6.**
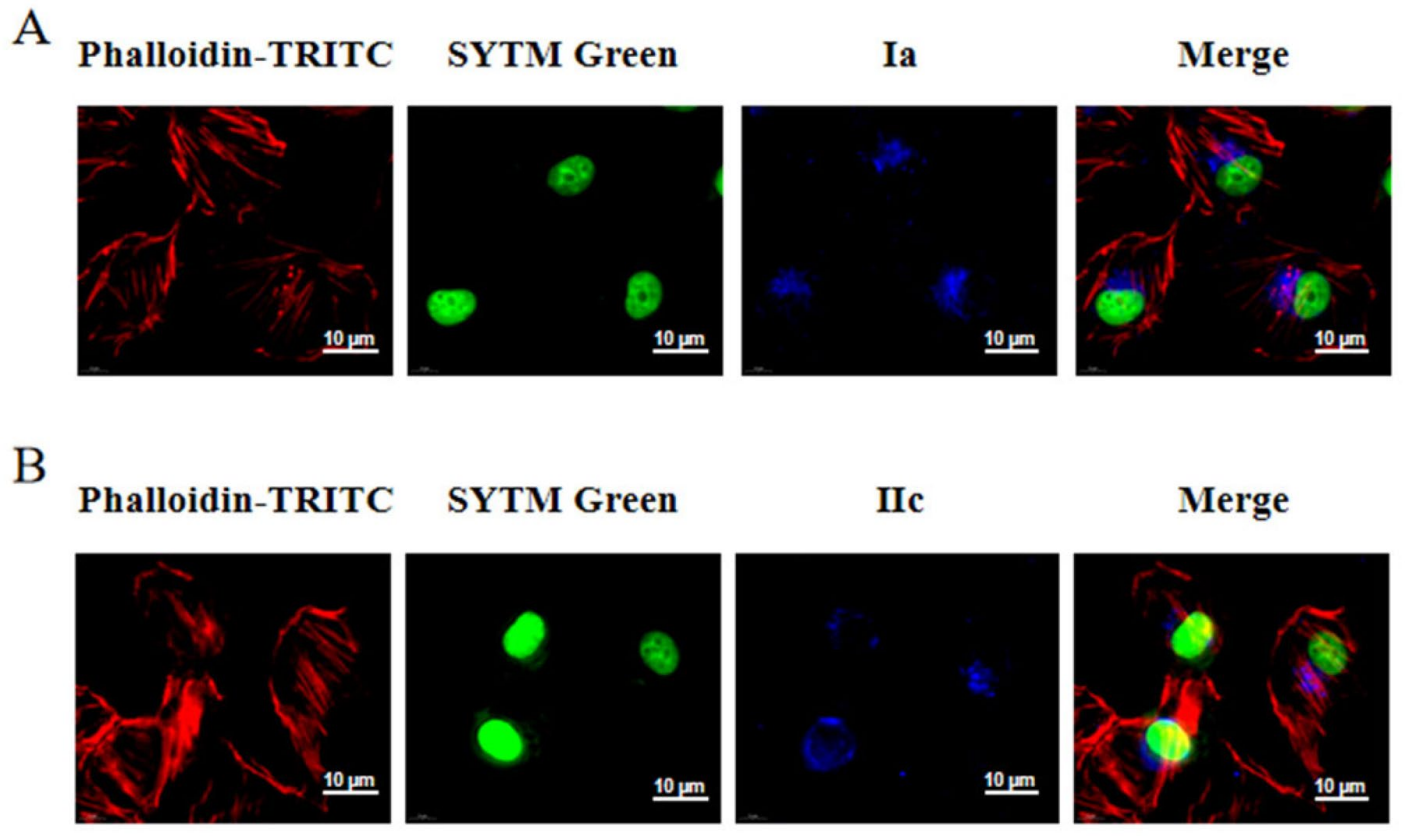
Cytoskeleton-nucleus dual staining to verify the subcellular location of probes **Ia** and **IIc**. Scale bar: 10 µm. **Ia**, **IIc**: UV excitation wavelength 330–380 nm, emission wavelength 420 nm (blue); Phalloidin-TRITC excitation wavelength of 540–546 nm, emission wavelength of 565–575 nm (red); SYTM Green excitation wavelength of 503 nm and an emission wavelength of 530 nm (green).

To further verify the subcellular localisation of the compound within cells, we employed a dual-labelling technique based on preliminary experiments (cytoskeleton staining only) to simultaneously stain the cytoskeleton (F-actin) and the nucleus (SYTM Green staining). The results (Figure 6) showed that the probe molecules exhibited a specific distribution in the perinuclear region. Most of the probe molecules (**Ia** and **IIc**) signals were clustered on one side of the perinuclear region, adjacent to the nuclear envelope structure, but did not enter the nucleus. This result suggested that the primary target of 18*β*-GA may be proteins located in the cytoplasm, exerting functional regulatory effects in the perinuclear region. This provides crucial clues for subsequent studies on its target proteins.

## Conclusion

The three types of probe molecules were synthesised using 18*β*-GA as the active group. Based on retaining the activity of 18*β*-GA, the C-3 hydroxyl and C-30 carboxyl moieties on their structures were modified, and azide acid, azide alcohol, and polyethylene glycol compounds were used as linkers. Dansulfonyl chloride compounds were introduced as fluorescent labelling groups to synthesise a series of 18*β*-GA photoaffinity-labelled molecular probes that could be used to identify anti-inflammatory target proteins.

The results of cell viability testing indicated that the probes inhibited the release of inflammation-related factors to varying degrees, and even some probes had better activity than 18*β*-GA. To perform pull-down experiments using photoaffinity labelling, high-throughput mass spectrometry technology was used to directly and systematically identify protein targets that specifically bind to 18*β*-GA in macrophages, providing an effective tool molecule. Unfortunately, this study has not yet performed covalent binding studies of intracellular proteins using active probes. The fluorescence distribution signals of probes **Ia** and **IIc** used for cell localisation were strong in the cytoplasm. Therefore, we speculated that the main target of 18*β*-GA may be a protein located in the cytoplasm. Therefore, the results of this study suggested that the main target of 18*β*-GA may be a protein located in the cytoplasm, greatly narrowing the screening range of target proteins.

## Materials and methods

### Chemistry

NMR spectra were recorded using a Bruker Ascend 600 MHz and a Bruker Avance 400 MHz, according to residual solvent signals of TMS (*δ* = 0.00 ppm). HRMS data were obtained using a 6200 series TOF G6203A high-resolution mass spectrometer and a SCIEX X500 Mass Spectrometer. Reverse-phase high-performance liquid chromatography was performed using an Agilent 1260 high-performance liquid chromatograph. The reaction was monitored on GF254 high-performance silica gel plates (TLC), and a high-pressure preparative chromatography system was used to separate and purify all target compounds. 18*β*-GA was purchased from Sa En Chemical Technology (Shanghai) Co., Ltd. All raw materials, reagents, and solvents were commercially available and used as received.

#### 10–(2-Azidoacetoxy)-2,4a,6a,6b,9,9,12a-heptamethyl-13-oxo-1,2,3,4,4a,5,6,6a,6b,7,8,8a,9,10,11,12,12a,12b,13,14b-icosahydropicene-2-carboxylic acid (9a)

The compound azidoacetic acid **8a** (0.12 g, 1.2 mmol) was dissolved in DCM. The EDCI (0.178 g, 0.929 mmol) and DMAP (0.146 g, 1.20 mmol) were added while stirring in an ice bath. After 15 min, 18*β*-GA (0.5 g, 0.6 mmol) was added. The reaction mixture was incubated at r.t. for 14 h. The reaction mixture was then poured into water, and the products were extracted with DCM (150 ml × 3). The extracted material was washed with brine and dried over Na_2_SO_4_. After removal of the solvent in vacuum, the residue was purified (silica gel, PE/EAC = 25%-30%, v/v) to obtain the white solid compound **9a** (0.116 g). Yield: 32%. HPLC analysis: 100%. m.p. 235–240 °C.^1^H-NMR (600 MHz, CDCl_3_) *δ* 5.72 (s, 1H), 4.65 (dd, *J* = 11.8, 4.7 Hz, 1H), 3.90 − 3.83 (m, 2H), 2.84 (dt, *J* = 13.5, 3.3 Hz, 1H), 2.38 (s, 1H), 2.20 (dd, *J* = 13.3, 3.5 Hz, 1H), 2.03 (ddd, *J* = 24.9, 11.1, 4.6 Hz, 2H), 1.94 (dt, *J* = 14.2, 3.9 Hz, 1H), 1.84 (td, *J* = 13.6, 4.3 Hz, 1H), 1.76 (qd, *J* = 13.2, 3.5 Hz, 1H), 1.71 − 1.57 (m, 4H), 1.51 − 1.45 (m, 1H), 1.43 (d, *J* = 9.6 Hz, 3H), 1.38 (s, 3H), 1.34 (dd, *J* = 15.5, 6.6 Hz, 1H), 1.23 (s, 4H), 1.17 (s, 3H), 1.14 (s, 3H), 1.11 − 1.01 (m, 2H), 0.91 (s, 6H), 0.84 (s, 3H), 0.82 (d, *J* = 11.6 Hz, 1H).^13^C-NMR (151 MHz, CDCl_3_) *δ* 200.27, 181.76, 169.61, 168.19, 128.44, 82.92, 61.66, 55.02, 50.68, 48.26, 45.47, 43.82, 43.24, 40.86, 38.72, 38.14, 37.72, 36.93, 32.67, 31.88, 30.91, 28.56, 28.46, 28.11, 26.49, 26.40, 23.56, 23.39, 18.69, 17.37, 16.75, 16.41. HRMS (ESI-TOF) calculated for C_32_H_47_N_3_O_5_ (M + H)^+^: 554.3594, observed: 554.3580 (Figure 1–3 for supplementary file 1).

#### 10-((4-Azidobutanoyl)oxy)-2,4a,6a,6b,9,9,12a-heptamethyl-13-oxo-1,2,3,4,4a,5,6,6a,6b,7,8,8a,9,10,11,12,12a,12b,13,14b-icosahydropicene-2-carboxylic acid (9b)

The compound 4-azidobutyric acid **8b** (0.548 g, 4.24 mmol) was dissolved in DCM. The EDCI (0.628 g, 6.36 mmol) and DMAP (0.518 g, 4.24 mmol) were added while stirring in an ice bath. After 15 min, 18*β*-GA (1.00 g, 2.12 mmol) was added. The reaction mixture was heated to room temperature for 12 h. The reaction mixture was then poured into water, and the products were extracted with DCM (120 ml × 3). The extracted material was washed with brine and dried over Na_2_SO_4_. After removing the solvent under vacuum, the residue was purified (silica gel, PE/EAC = 22%, v/v) to obtain the white solid compound **9b** (0.293 g). Yield: 24%. HPLC analysis: 100%. m.p. 245–255 °C. ^1^H-NMR (600 MHz, DMSO-*d*_6_) *δ* 12.19 (s, 1H), 5.41 (s, 1H), 4.46 (dd, *J* = 11.8, 4.4 Hz, 1H), 3.36 (t, *J* = 6.8 Hz, 2H), 2.67 − 2.61 (m, 1H), 2.38 (td, *J* = 16.0, 15.0, 7.9 Hz, 3H), 2.09 (ddd, *J* = 17.9, 12.5, 4.5 Hz, 2H), 1.84 − 1.73 (m, 4H), 1.71 − 1.60 (m, 4H), 1.55 − 1.46 (m, 2H), 1.44 − 1.32 (m, 7H), 1.26 (td, *J* = 14.0, 3.2 Hz, 1H), 1.15 (d, *J* = 13.3 Hz, 1H), 1.12 − 1.03 (m, 10H), 0.96 (d, *J* = 13.6 Hz, 1H), 0.90 (d, *J* = 11.7 Hz, 1H), 0.83 (s, 6H), 0.76 (s, 3H).^13^C-NMR (151 MHz, DMSO-*d*_6_) *δ* 199.36, 178.12, 172.39, 170.31, 127.70, 80.29, 61.28, 54.14, 50.47, 48.52, 45.32, 43.54, 43.42, 41.08, 38.32, 38.09, 37.98, 36.96, 32.37, 31.99, 31.49, 30.81, 28.85, 28.27, 28.13, 26.55, 26.25, 24.45, 23.65, 23.47, 18.78, 17.38, 17.08, 16.61. HRMS (ESI-TOF) calculated for C_34_H_51_N_3_O_5_ (M + H)^+^: 582.3907, observed: 582.3898 (Figure 4–6 for supplementary file 1).

#### 10-((5-Azidopentanoyl)oxy)-2,4a,6a,6b,9,9,12a-heptamethyl-13-oxo-1,2,3,4,4a,5,6,6a,6b,7,8,8a,9,10,11,12,12a,12b,13,14b-icosahydropicene-2-carboxylic acid (9c)

The compound 5-azidovaleric acid **8c** (0.320 g, 2.12 mmol) was dissolved in DCM. The EDCI (0.414 g, 3.18 mmol) and DMAP (0.258 g, 2.12 mmol) were added while stirring in an ice bath. After 15 min, 18*β*-GA (0.500 g, 1.06 mmol) was added. The reaction mixture was heated to room temperature for 14 h. The reaction mixture was then poured into water, and the products were extracted with DCM (150 ml × 3). The extracted material was washed with brine and dried over Na_2_SO_4_. After removing the solvent under vacuum, the residue was purified (silica gel, PE/EAC = 22%, v/v) to obtain the white solid compound **9c** (0.185 g). Yield: 30%. HPLC analysis: 97%. m.p. 245–250 °C. ^1^H-NMR (600 MHz, CDCl_3_) *δ* 5.72 (s, 1H), 4.54 (dd, *J* = 11.8, 4.6 Hz, 1H), 3.30 (t, *J* = 6.7 Hz, 2H), 2.80 (dt, *J* = 13.4, 3.2 Hz, 1H), 2.36 (dd, *J* = 13.8, 6.6 Hz, 3H), 2.19 (dd, *J* = 13.3, 3.4 Hz, 1H), 2.02 (ddd, *J* = 24.8, 11.1, 3.4 Hz, 2H), 1.94 (dt, *J* = 13.5, 3.2 Hz, 1H), 1.84 (td, *J* = 13.6, 4.3 Hz, 1H), 1.72 (tdd, *J* = 14.5, 9.8, 6.0 Hz, 3H), 1.68 − 1.59 (m, 5H), 1.50 − 1.39 (m, 4H), 1.37 (s, 3H), 1.36 − 1.30 (m, 1H), 1.28 − 1.11 (m, 11H), 1.09 − 1.01 (m, 2H), 0.88 (d, *J* = 4.8 Hz, 6H), 0.84 (s, 4H).^13^C-NMR (151 MHz, CDCl_3_) *δ* 200.38, 181.71, 172.97, 169.53, 128.44, 80.66, 61.70, 55.02, 51.10, 48.26, 45.48, 43.82, 43.23, 40.84, 38.76, 38.11, 37.72, 36.95, 34.15, 32.70, 31.88, 30.91, 28.56, 28.46, 28.37, 28.12, 26.48, 26.41, 23.62, 23.38, 22.28, 18.69, 17.38, 16.78, 16.41. HRMS (ESI-TOF) calculated for C_35_H_53_N_3_O_5_ (M + H)^+^: 596.4063, observed: 596.4063 (Figure 7–9 for supplementary file 1).

#### 10-((6-Azidohexanoyl)oxy)-2,4a,6a,6b,9,9,12a-heptamethyl-13-oxo-1,2,3,4,4a,5,6,6a,6b,7,8,8a,9,10,11,12,12a,12b,13,14b-icosahydropicene-2-carboxylic acid (9d)

The compound 6-azido-hexanoic acid **8d** (0.333 g, 2.12 mmol) was dissolved in DCM. The EDCI (0.315 g, 3.18 mmol) and DMAP (0.260 g, 2.12 mmol) were added while stirring in an ice bath. After 15 min, 18*β*-GA (0.500 g, 1.06 mmol) was added. The reaction mixture was heated to room temperature for 8 h. The reaction mixture was then poured into water, and the products were extracted with DCM (120 ml × 3). The extracted material was washed with brine and dried over Na_2_SO_4_. After removing the solvent under vacuum, the residue was purified (silica gel, PE/EAC = 25%, v/v) to obtain the white solid compound **9d** (0.215 g). Yield: 33%. HPLC analysis: 100%. m.p. 230–235 °C. ^1^H-NMR (600 MHz, CDCl_3_) *δ* 5.72 (s, 1H), 4.53 (dd, *J* = 11.8, 4.6 Hz, 1H), 3.28 (t, *J* = 6.9 Hz, 2H), 2.80 (dt, *J* = 13.3, 3.2 Hz, 1H), 2.37 (s, 1H), 2.33 (t, *J* = 7.4 Hz, 2H), 2.19 (dd, *J* = 13.4, 3.4 Hz, 1H), 2.01 (td, *J* = 13.3, 3.5 Hz, 2H), 1.94 (dt, *J* = 13.5, 3.2 Hz, 1H), 1.84 (td, *J* = 13.6, 4.3 Hz, 1H), 1.73 − 1.65 (m, 4H), 1.64 − 1.57 (m, 5H), 1.49 − 1.45 (m, 1H), 1.44 − 1.40 (m, 5H), 1.37 (s, 3H), 1.34 (dd, *J* = 15.5, 6.6 Hz, 1H), 1.25 (s, 1H), 1.23 (s, 3H), 1.20 (d, *J* = 3.9 Hz, 1H), 1.17 (s, 3H), 1.13 (s, 3H), 1.09 − 1.01 (m, 2H), 0.88 (s, 3H), 0.87 (s, 3H), 0.84 (s, 3H), 0.81 (d, *J* = 11.1 Hz, 1H). ^13^C-NMR (151 MHz, CDCl_3_) *δ* 200.40, 181.46, 173.31, 169.52, 128.46, 80.52, 61.71, 55.03, 51.26, 48.27, 45.49, 43.81, 43.23, 40.86, 38.77, 38.11, 37.72, 36.96, 34.58, 32.71, 30.92, 29.72, 28.58, 28.55, 28.46, 28.10, 26.48, 26.41, 26.30, 24.64, 23.62, 23.38, 18.69, 17.38, 16.78, 16.42. HRMS (ESI-TOF) calculated for C_36_H_55_N_3_O_5_ (M + H)^+^: 610.4220, observed: 610.4219 (Figure 10–12 for supplementary file 1).

#### 10–(2-(4-(((N^2^-(3-Benzoylbenzoyl)-N^6^-((5-(dimethylamino)naphthalen-1-yl)sulphonyl)lysyl)oxy)methyl)-1H-1,2,3-triazol-1-yl)acetoxy)-2,4a,6a,6b,9,9,12a-heptamethyl-13-oxo-1,2,3,4,4a,5,6,6a,6b,7,8,8a,9,10,11,12,12a,12b,13,14b-icosahydropicene-2-carboxylic acid (Ia)

The compound **7** (0.036 g, 0.058 mmol) was dissolved in a mixed solvent of THF and water (V/V = 1/1, 10 ml), and the compound **9a** (0.04 g, 0.07 mmol), CuSO_4_·5H_2_O (0.00300 g, 0.0116 mmol), and sodium ascorbate (0.004 g, 0.02 mmol) were added in sequence while stirring for 22 h at r.t. The reaction mixture was then poured into water, and the products were extracted with DCM (150 ml × 3). The extracted material was washed with brine and dried over Na_2_SO_4_. After removing the solvent under vacuum, the residue was purified (silica gel, MeOH/DCM = 3%, v/v) to obtain the light yellow solid compound **Ia** (0.059 g). Yield: 86%. m.p. 175–180 °C. HPLC analysis: 98%. ^1^H-NMR (600 MHz, CDCl_3_) *δ* 8.52 (d, *J* = 8.5 Hz, 1H), 8.27 (d, *J* = 8.6 Hz, 1H), 8.18 (d, *J* = 7.2 Hz, 1H), 7.93 (d, *J* = 8.3 Hz, 2H), 7.87 (s, 1H), 7.84 (d, J = 8.2 Hz, 2H), 7.79 (d, *J* = 7.3 Hz, 2H), 7.61 (t, *J* = 7.4 Hz, 1H), 7.49 (q, *J* = 7.4 Hz, 4H), 7.16 (d, *J* = 7.5 Hz, 1H), 7.02 (d, *J* = 6.7 Hz, 1H), 5.68 (s, 1H), 5.42 (d, *J* = 12.8 Hz, 1H), 5.33 − 5.28 (m, 2H), 5.29 − 5.16 (m, 3H), 4.77 (q, *J* = 7.3 Hz, 1H), 4.60 (dd, *J* = 11.6, 4.5 Hz, 1H), 2.87 (s, 8H), 2.79 (d, *J* = 13.7 Hz, 1H), 2.34 (s, 1H), 2.18 (d, *J* = 15.6 Hz, 1H), 2.06 − 1.96 (m, 2H), 1.95 − 1.74 (m, 5H), 1.74 − 1.57 (m, 5H), 1.48 − 1.37 (m, 8H), 1.35 (s, 4H), 1.25 (s, 1H), 1.21 (s, 3H), 1.17 (d, *J* = 12.7 Hz, 1H), 1.11 (d, *J* = 9.5 Hz, 6H), 1.02 (d, *J* = 12.2 Hz, 2H), 0.83 (d, *J* = 14.4 Hz, 6H), 0.79 (d, *J* = 8.9 Hz, 4H). ^13^C-NMR (151 MHz, CDCl_3_) *δ* 200.17, 195.97, 180.81, 172.10, 169.74, 166.46, 166.03, 151.97, 142.51, 140.36, 137.01, 136.87, 134.85, 132.91, 130.38, 130.13, 130.11, 129.89, 129.60, 129.32, 128.47, 128.37, 128.34, 127.26, 125.58, 123.19, 118.74, 115.23, 83.65, 61.56, 58.48, 54.82, 53.44, 52.61, 51.20, 48.28, 45.43, 45.41, 43.79, 43.23, 42.59, 40.92, 38.57, 38.10, 37.71, 36.86, 32.58, 31.87, 31.52, 30.95, 29.70, 28.81, 28.55, 28.44, 28.08, 26.48, 26.37, 23.46, 23.38, 21.89, 18.66, 17.31, 16.58, 16.39. HRMS (ESI-TOF) calculated for C_67_H_82_N_6_O_11_S (M-H)^-^: 1177.5684, observed: 1177.5712 (Figure 13–15 for supplementary file 1).

#### 10-((4–(4-(((N^2^-(3-Benzoylbenzoyl)-N^6^-((5-(dimethylamino)naphthalen-1-yl)sulphonyl)lysyl)oxy)methyl)-1H-1,2,3-triazol-1-yl)butanoyl)oxy)-2,4a,6a,6b,9,9,12a-heptamethyl-13-oxo-1,2,3,4,4a,5,6,6a,6b,7,8,8a,9,10,11,12,12a,12b,13,14b-icosahydropicene-2-carboxylic acid (Ib)

The compound **7** (0.181 g, 0.290 mmol) was dissolved in a mixed solvent of THF and water (V/V = 1/1, 20 ml), and the compound **9b** (0.205 g, 0.350 mmol), CuSO_4_·5H_2_O (0.015 g, 0.060 mmol) and sodium ascorbate (0.016 g, 0.080 mmol) were added in sequence while stirring for 20 h at r.t. The reaction mixture was then poured into water, and the products were extracted with DCM (120 ml × 3). The extracted material was washed with brine and dried over Na_2_SO_4_. After removal of the solvent in vacuum, the residue was purified (silica gel, MeOH/DCM = 3%, v/v) to obtain pale yellow solid compound **Ib** (0.17 g). Yield: 49%. m.p. 150–155 °C. HPLC analysis: 100%. ^1^H-NMR (600 MHz, DMSO-*d*_6_) *δ* 12.20 (s, 1H), 8.87 (d, *J* = 7.2 Hz, 1H), 8.44 (d, *J* = 8.5 Hz, 1H), 8.29 (d, *J* = 8.6 Hz, 1H), 8.14 (s, 1H), 8.09 − 8.06 (m, 1H), 8.00 (d, *J* = 8.3 Hz, 2H), 7.89 (t, *J* = 5.8 Hz, 1H), 7.81 (d, *J* = 8.3 Hz, 2H), 7.78 − 7.74 (m, 2H), 7.71 (t, *J* = 7.4 Hz, 1H), 7.62 − 7.54 (m, 4H), 7.23 (d, *J* = 7.5 Hz, 1H), 5.41 (s, 1H), 5.18 (s, 2H), 4.44 (dd, *J* = 11.8, 4.4 Hz, 1H), 4.39 (t, *J* = 7.0 Hz, 2H), 4.30 (q, *J* = 7.1 Hz, 1H), 2.81 (s, 6H), 2.78 − 2.72 (m, 2H), 2.63 (dd, *J* = 10.4, 3.2 Hz, 1H), 2.39 (s, 1H), 2.30 (tt, *J* = 16.6, 8.1 Hz, 2H), 2.13 − 2.02 (m, 4H), 1.82 − 1.60 (m, 8H), 1.53 − 1.43 (m, 2H), 1.40 − 1.22 (m, 12H), 1.15 − 1.01 (m, 11H), 0.96 (d, *J* = 13.0 Hz, 1H), 0.87 (d, *J* = 11.9 Hz, 1H), 0.83 − 0.74 (m, 9H). ^13^C-NMR (151 MHz, DMSO-*d*_6_) *δ* 199.38, 195.86, 172.25, 172.15, 170.80, 170.34, 166.46, 151.79, 142.23, 139.87, 137.45, 137.10, 136.60, 133.54, 130.21, 129.91, 129.79, 129.55, 129.51, 129.15, 128.62, 128.22, 128.14, 127.68, 125.15, 124.02, 119.59, 115.55, 80.38, 61.27, 58.21, 55.39, 54.13, 53.30, 49.06, 48.52, 45.52, 45.31, 43.54, 43.41, 42.64, 41.09, 38.30, 38.07, 37.98, 36.94, 32.35, 31.99, 31.11, 30.23, 29.24, 28.85, 28.28, 28.15, 26.54, 26.24, 25.75, 23.64, 23.46, 23.22, 18.76, 17.36, 17.06, 16.60. HRMS (ESI-TOF) calculated for C_69_H_86_N_6_O_11_S (M-H)^-^: 1205.5997; observed: 1205.6023 (Figure 16–18 for supplementary file 1).

#### 10-((5–(4-(((N^2^-(3-Benzoylbenzoyl)-N^6^-((5-(dimethylamino)naphthalen-1-yl)sulphonyl)lysyl)oxy)methyl)-1H-1,2,3-triazol-1-yl)pentanoyl)oxy)-2,4a,6a,6b,9,9,12a-heptamethyl-13-oxo-1,2,3,4,4a,5,6,6a,6b,7,8,8a,9,10,11,12,12a,12b,13,14b-icosahydropicene-2-carboxylic acid (Ic)

The compound **7** (0.119 g, 0.190 mmol) was dissolved in a mixed solvent of THF and water (V/V = 1/1, 8 ml), and the compound **9c** (0.14 g, 0.23 mmol), CuSO_4_·5H_2_O (0.0090 g, 0.038 mmol), and sodium ascorbate (0.012 g, 0.060 mmol) were added in sequence while stirring for 22 h at r.t. The reaction mixture was then poured into water, and the products were extracted with DCM (120 ml × 3). The extracted material was washed with brine and dried over Na_2_SO_4_. After removal of the solvent in vacuum, the residue was purified (silica gel, MeOH/DCM = 3%, v/v) to obtain the pale yellow solid compound **Ic** (0.17 g). Yield: 73%. m.p. 145–150 °C. HPLC analysis: 100%. ^1^H-NMR (600 MHz, CDCl_3_) *δ* 8.53 (d, *J* = 8.2 Hz, 1H), 8.30 (d, *J* = 8.4 Hz, 1H), 8.20 (d, *J* = 7.1 Hz, 1H), 7.95 (d, *J* = 8.1 Hz, 2H), 7.84 (d, *J* = 8.2 Hz, 2H), 7.79 (d, *J* = 7.2 Hz, 2H), 7.71 (s, 1H), 7.61 (t, *J* = 7.4 Hz, 1H), 7.50 (dt, *J* = 11.5, 7.9 Hz, 4H), 7.17 (d, *J* = 7.4 Hz, 1H), 7.06 (d, *J* = 6.9 Hz, 1H), 5.68 (s, 1H), 4.76 (q, *J* = 6.7 Hz, 1H), 4.51 (dd, *J* = 11.8, 4.6 Hz, 1H), 4.40 (t, *J* = 6.9 Hz, 2H), 2.88 (s, 8H), 2.80 − 2.74 (m, 1H), 2.37 − 2.34 (m, 3H), 2.18 (dd, *J* = 13.2, 3.4 Hz, 1H), 2.05 − 1.90 (m, 6H), 1.82 (td, *J* = 14.1, 4.6 Hz, 2H), 1.64 (tt, *J* = 21.3, 10.2 Hz, 6H), 1.45 (dq, *J* = 34.5, 9.0, 8.2 Hz, 9H), 1.38 − 1.29 (m, 5H), 1.26 (q, *J* = 5.7, 4.1 Hz, 1H), 1.23 − 1.10 (m, 11H), 1.05 − 1.00 (m, 2H), 0.85 (s, 9H), 0.79 (d, *J* = 11.6 Hz, 1H). ^13^C-NMR (151 MHz, CDCl_3_) *δ* 200.36, 196.01, 180.63, 172.81, 172.14, 169.69, 166.53, 142.19, 140.35, 137.02, 136.90, 134.87, 132.92, 130.33, 130.13, 129.86, 129.61, 129.40, 128.47, 128.39, 128.35, 127.30, 123.90, 123.26, 118.82, 115.26, 80.84, 61.67, 58.65, 54.96, 53.45, 52.62, 50.14, 48.30, 45.46, 45.45, 43.76, 43.23, 42.54, 40.92, 38.71, 38.07, 37.70, 36.93, 33.80, 32.66, 31.88, 31.45, 30.95, 29.71, 29.58, 28.76, 28.54, 28.43, 28.14, 26.48, 26.39, 23.63, 23.37, 21.95, 21.92, 18.68, 17.36, 16.78, 16.43. HRMS (ESI-TOF) calculated for C_70_H_88_N_6_O_11_S (M-H)^-^: 1219.6154; observed: 1219.6182 (Figure 19–21 for supplementary file 1).

#### 10-((6–(4-(((N^2^-(3-Benzoylbenzoyl)-N^6^-((5-(dimethylamino)naphthalen-1-yl)sulphonyl)lysyl)oxy)methyl)-1H-1,2,3-triazol-1-yl)hexanoyl)oxy)-2,4a,6a,6b,9,9,12a-heptamethyl-13-oxo-1,2,3,4,4a,5,6,6a,6b,7,8,8a,9,10,11,12,12a,12b,13,14b-icosahydropicene-2-carboxylic acid (Id)

The compound **7** (0.026 g, 0.042 mmol) was dissolved in a mixed solvent of THF and water (V/V = 1/1, 8 ml), and the compound **9d** (0.030 g, 0.050 mmol), CuSO_4_·5H_2_O (0.002 g, 0.008 mmol), and sodium ascorbate (0.002 g, 0.01 mmol) were added in sequence while stirring for 8 h at r.t. The reaction mixture was then poured into water, and the products were extracted with DCM (120 ml × 3). The extracted material was washed with brine and dried over Na_2_SO_4_. After removal of the solvent under vacuum, the residue was purified (silica gel, MeOH/DCM = 3%, v/v) to obtain the pale yellow solid compound **Id** (0.036 g). Yield: 70%. m.p. 165–170 °C. HPLC analysis: 100%. ^1^H-NMR (600 MHz, DMSO-*d*_6_) *δ* 8.86 (d, *J* = 7.2 Hz, 1H), 8.44 (d, *J* = 8.5 Hz, 1H), 8.29 (d, *J* = 8.6 Hz, 1H), 8.12 (s, 1H), 8.08 (dd, *J* = 7.2, 0.9 Hz, 1H), 8.00 (d, *J* = 8.3 Hz, 2H), 7.89 (t, *J* = 5.8 Hz, 1H), 7.81 (d, *J* = 8.3 Hz, 2H), 7.78 − 7.74 (m, 2H), 7.71 (t, *J* = 7.4 Hz, 1H), 7.62 − 7.55 (m, 4H), 7.23 (d, *J* = 7.5 Hz, 1H), 5.41 (s, 1H), 5.17 (s, 2H), 4.41 (dd, *J* = 11.8, 4.4 Hz, 1H), 4.32 (dt, *J* = 19.0, 7.1 Hz, 3H), 2.81 (s, 6H), 2.76 (q, *J* = 6.9 Hz, 2H), 2.62 (dd, *J* = 10.3, 3.2 Hz, 1H), 2.39 (s, 1H), 2.25 (hept, *J* = 7.7, 7.3 Hz, 2H), 2.08 (td, *J* = 12.4, 11.7, 4.0 Hz, 2H), 1.79 (p, *J* = 7.5, 7.0 Hz, 3H), 1.72 (dd, *J* = 14.3, 4.3 Hz, 1H), 1.69 − 1.64 (m, 4H), 1.60 (dd, *J* = 13.8, 3.5 Hz, 1H), 1.52 (dt, *J* = 21.9, 11.0 Hz, 3H), 1.43 (dd, *J* = 13.4, 3.9 Hz, 1H), 1.34 (d, *J* = 9.7 Hz, 10H), 1.24 (ddd, *J* = 27.2, 15.1, 5.8 Hz, 5H), 1.10 (s, 5H), 1.04 (d, *J* = 12.2 Hz, 6H), 0.95 (d, *J* = 12.1 Hz, 1H), 0.86 (d, *J* = 11.7 Hz, 1H), 0.79 (d, *J* = 4.1 Hz, 6H), 0.75 (s, 3H).^13^C-NMR (151 MHz, DMSO-*d*_6_) *δ* 199.39, 195.86, 178.13, 172.87, 172.24, 170.32, 166.46, 151.79, 142.07, 139.88, 137.46, 137.10, 136.59, 133.54, 130.20, 129.91, 129.79, 129.55, 129.52, 129.15, 128.61, 128.22, 128.14, 127.68, 125.02, 124.02, 119.59, 115.55, 79.98, 61.28, 58.24, 54.14, 53.30, 49.66, 48.52, 45.52, 45.31, 43.54, 43.40, 42.64, 41.08, 38.31, 38.04, 37.98, 36.94, 34.17, 32.35, 31.99, 30.81, 30.23, 29.82, 29.24, 28.84, 28.27, 28.12, 26.53, 26.24, 25.78, 24.43, 23.66, 23.45, 23.22, 18.77, 17.37, 17.06, 16.61. HRMS (ESI-TOF) calculated for C_71_H_90_N_6_O_11_S (M-H)^-^: 1233.6310, observed: 1233.6361 (Figure 22–24 for supplementary file 1).

#### General procedure for synthesis of compounds 10a-10d

The compound **7** (0.30 g, 0.48 mmol) and the corresponding Azido-PEG_(n)_-alcohol (0.075 g, 0.57 mmol) were dissolved in a mixed solvent of THF and water (V/V = 1/1, 30 ml) at r.t. CuSO_4_·5H_2_O (0.026 g, 0.010 mmol) and sodium ascorbate (0.04 g, 0.2 mmol) were then added in sequence while stirring. Next, the reaction process was moved to 30 °C for 6 h. Finally, the product was purified (silica gel, MeOH/DCM = 2%–4%, V/V) to obtain compounds **10a**-**10d**.

##### (1–(2-Hydroxyethyl)-1*H*-1,2,3-triazol-4-yl)methyl *N*^2^-(4-benzoylbenzoyl)-*N*^6^-((5-(dimethylamino)naphthalen-1-yl)sulphonyl)-*L*-lysinate (**10a**)

A light yellow solid was obtained in 53% yield. HPLC analysis: 98%. m.p. 79–81 °C. ^1^H-NMR (400 MHz, CDCl_3_) *δ* 8.56 (d, *J* = 7.6 Hz, 1H), 8.31 (d, *J* = 8.2 Hz, 1H), 8.17 (d, *J* = 6.0 Hz, 1H), 7.88 (t, *J* = 8.4 Hz, 3H), 7.77 (t, *J* = 6.8 Hz, 4H), 7.60 (t, *J* = 7.6 Hz, 1H), 7.53 − 7.46 (m, 4H), 7.24 (s, 1H), 7.19 (d, *J* = 6.8 Hz, 1H), 5.65 (s, 1H), 5.31 (q, *J* = 12.4 Hz, 2H), 4.63 (q, *J* = 7.6 Hz,1H), 4.50 (t, *J* = 4.44 Hz, 2H), 4.01 (t, *J* = 4.8 Hz, 2H), 2.90 (s, 6H), 2.87 − 2.84 (m, 2H), 1.83 − 1.78 (m, 3H), 1.42 − 1.41 (m, 4H). ^13^C NMR (100 MHz, CDCl_3_) *δ* 196.04, 172.08, 166.86, 142.18, 140.32, 136.90, 136.71, 132.95, 130.11, 130.06, 129.53, 129.39, 128.47, 128.30, 127.30, 125.23, 77.35, 77.24, 77.03, 76.72, 61.04, 58.48, 53.06, 52.83, 45.54, 42.49, 30.96, 28.75, 22.03. Chemical Formula: C_37_H_40_N_6_O_7_S; ESI-MS *m/z*: 713.3 (M + H)^+^ (Figure 25–27 for supplementary file 1).

##### (1–(2-(2-Hydroxyethoxy)ethyl)-1*H*-1,2,3-triazol-4-yl)methyl *N^2^*-(4-benzoylbenzoyl)-*N^6^*-((5-(dimethylamino)naphthalen-1-yl)sulphonyl)-*L*-lysinate (**10b**)

A light green solid was obtained in 83% yield. HPLC analysis: 98%. m.p. 53–57 °C. ^1^H NMR (600 MHz, DMSO-*d*_6_) *δ* 8.87 (d, *J* = 7.2 Hz, 1H), 8.44 (d, *J* = 9.0 Hz, 1H), 8.29 (d, *J* = 9.0 Hz, 1H), 8.13 (s, 1H), 8.08 (dd, *J* = 7.2, 1.2 Hz, 1H), 8.00 (d, *J* = 12.0 Hz, 2H), 7.88 (t, *J* = 6.0 Hz, 1H), 7.81 (d, *J* = 8.4 Hz, 2H), 7.77 − 7.76 (m, 2H), 7.73 − 7.70 (m, 1H), 7.61 − 7.55 (m, 4H), 7.23 (d, *J* = 7.8 Hz, 1H), 5.18 (s, 2H), 4.61 (t, *J* = 5.4 Hz, 1H), 4.52 (t, *J* = 5.4 Hz, 2H), 4.32 (q, *J* = 7.2 Hz, 1H), 3.80 (t, *J* = 4.8 Hz, 2H), 3.47 − 3.34 (m, 2H), 3.42 − 3.40 (m, 2H), 2.81 (s, 6H), 2.77 − 2.76 (m, 2H), 1.67 − 1.63 (m, 2H), 1.36 − 1.25 (m, 4H). ^13^C NMR (150 MHz, DMSO-*d*_6_) *δ* 195.90, 172.26, 166.48, 151.80, 141.98, 139.89, 137.46, 137.11, 136.58, 133.54, 130.21, 129.92, 129.80, 129.55, 129.51, 129.16, 128.64, 128.23, 128.15, 125.62, 124.04, 119.58, 115.56, 72.53, 69.11, 60.57, 58.23, 53.27, 49.93, 45.52, 42.65, 40.41, 40.27, 40.13, 40.00, 39.86, 39.72, 39.58, 30.26, 29.25, 23.24. Chemical Formula: C_39_H_44_N_6_O_8_S; HRMS (ESI-TOF) calculated for C_39_H_45_N_6_O_8_S (M + H)^+^: 757.3020; observed: 757.3020 (Figure 28–30 for supplementary file 1).

##### (1–(2–(2-(2-Hydroxyethoxy)ethoxy)ethyl)-1*H*-1,2,3-triazol-4-yl)methyl *N^2^*-(4-benzoylbenzoyl)-*N^6^*-((5-(dimethylamino)naphthalen-1-yl)sulphonyl)-*L*-lysinate (**10c**)

A light green solid was obtained in 79% yield. HPLC analysis: 95%. m.p. 46–50 °C. ^1^H NMR (600 MHz, DMSO-*d*_6_) *δ* 8.87 (d, *J* = 7.2 Hz, 1H), 8.44 (d, *J* = 8.4 Hz, 1H), 8.29 (d, *J* = 9.0 Hz, 1H), 8.12 (s, 1H), 8.08 (d, *J* = 6.6 Hz, 1H), 8.00 (d, *J* = 8.4 Hz, 2H), 7.88 (t, *J* = 5.4 Hz, 1H), 7.81 (d, *J* = 8.4 Hz, 2H), 7.76 (d, *J* = 7.2 Hz, 2H), 7.71 (t, *J* = 7.2 Hz, 1H), 7.61 − 7.55 (m, 4H), 7.23 (d, *J* = 7.2 Hz, 1H), 5.17 (s, 2H), 4.56 (t, *J* = 5.4, 1H), 4.52 (t, *J* = 4.8, 2H), 4.32 (q, *J* = 7.2 Hz, 1H), 3.80 (t, *J* = 4.8 Hz, 2H), 3.51 − 3.50 (m, 2H), 3.47 − 3.45 (m, 4H), 3.37 (t, *J* = 5.4 Hz, 2H), 2.81 (s, 6H), 2.78 − 2.74 (m, 2H), 1.67–1.63 (m, 2H), 1.37– 1.26 (m, 4H). ^13^C NMR (150 MHz, DMSO-*d*_6_) *δ* 195.90, 172.25, 166.47, 151.80, 141.97, 139.89, 137.47, 137.11, 136.58, 133.54, 130.20, 129.92, 129.80, 129.54, 129.51, 129.16, 128.64, 128.23, 128.15, 125.63, 124.04, 119.58, 115.56, 72.77, 70.06, 70.00, 69.11, 60.67, 58.22, 53.27, 49.87, 45.52, 42.65, 40.41, 40.27, 40.14, 40.00, 39.86, 39.72, 39.58, 30.27, 29.26, 23.24. Chemical Formula: C_41_H_48_N_6_O_9_S; HRMS (ESI-TOF) calculated for C_41_H_49_N_6_O_9_S (M + H)^+^: 801.3282; observed: 801.3229 (Figure 31–33 for supplementary file 1).

##### (1–(2–(2-(2-(2-Hydroxyethoxy)ethoxy)ethoxy)ethyl)-1*H*-1,2,3-triazol-4-yl)methyl *N*^2^–(4-benzoylbenzoyl)-*N*^6^-((5-(dimethylamino)naphthalen-1-yl)sulphonyl)-*L*-lysinate (**10d**)

A yellow oil was obtained in 85% yield. HPLC analysis: 99%. ^1^H NMR (400 MHz, DMSO-*d*_6_) *δ* 8.86 (d, *J* = 7.6 Hz, 1H), 8.44 (d, *J* = 8.4 Hz, 1H), 8.30 (d, *J* = 8.4 Hz, 1H), 8.11 (s, 1H), 8.09 (dd, *J* = 7.2, 1.2 Hz, 1H), 8.01 (d, *J* = 8.6 Hz, 2H), 7.87 (t, *J* = 5.8 Hz, 1H), 7.81 (d, *J* = 8.6 Hz, 2H), 7.78 − 7.76 (m, 2H), 7.74 − 7.70 (m, 1H), 7.62 − 7.55 (m, 4H), 7.23 (d, *J* = 8.0 Hz, 1H), 5.18 (s, 2H), 4.53 (t, *J* = 5.2 Hz, 2H), 4.33 (q, *J* = 6.8 Hz, 1H), 3.80 (t, *J* = 5.6 Hz, 2H), 3.51 − 3.45 (m, 11H), 3.40 (t, *J* = 4.8 Hz, 2H), 2.82 (s, 6H), 2.77 (q, *J* = 6.6 Hz, 2H), 1.66 (q, *J* = 7.2 Hz, 2H), 1.38 − 1.21 (m, 4H). ^13^C NMR (100 MHz, DMSO-*d*_6_) *δ* 195.89, 172.24, 166.47, 151.81, 141.99, 139.91, 137.50, 137.14, 136.62, 133.53, 130.19, 129.89, 129.80, 129.57, 129.54, 129.15, 128.62, 128.22, 128.15, 125.56, 124.02, 119.60, 115.56, 72.79, 70.25, 70.20, 70.10, 70.01, 69.12, 60.69, 58.23, 53.27, 49.90, 45.52, 42.66, 40.66, 40.45, 40.24, 40.03, 39.83, 39.62, 39.41, 30.30, 29.27, 23.23. Chemical Formula: C_43_H_52_N_6_O_10_S; ESI-MS *m/z*: 845.3 (M + H)^+^ (Figure 34–36 for supplementary file 1).

#### General procedure for synthesis of compounds 11a-11d

The compounds **10a**-**10d** (0.30 g, 0.40 mmol) were dissolved in DCM (20 ml). Under a N_2_ atmosphere, the DIPEA (0.180 g, 1.39 mmol) and corresponding sulphonyl chloride (0.240 g, 1.87 mmol) were added in an ice bath while stirring. Next, the reaction was moved to r.t. for 3 h. Finally, the product was purified (silica gel, MeOH/DCM = 2%-3%, v/v) to obtain crude compounds **11a**-**11d**.

#### General procedure for synthesis of compounds IIa-IId

The compound 18*β*-GA (0.15 g, 0.32 mmol) and the corresponding compounds **11a-11d** (0.24 g, 0.28 mmol) were dissolved in DMF (10 ml). K_2_CO_3_ (0.190 g, 1.37 mmol) was then added and stirred overnight at 75 °C. The reaction mixture was then poured into water, and the products were extracted using EAC. The extract was washed with saturated aqueous NaHCO_3_ and brine and dried over Na_2_SO_4_. After removal of the solvent in a vacuum, the residue was purified (silica gel, MeOH/DCM = 3%-4%, v/v) to obtain target compounds **IIa**–**IId**.

##### 2-(4-(((*N*^2^-(4-Benzoylbenzoyl)-*N*^6^-((5-(dimethylamino)naphthalen-1-yl)sulphonyl)-*L*-lysyl)oxy)methyl)-1*H*-1,2,3-triazol-1-yl)ethyl 10-hydroxy-2,4a,6a,6b,9,9,12a-heptamethyl-13-oxo-1,2,3,4,4a,5,6,6a,6b,7,8,8a,9,10,11,12,12a,12b,13,14b-icosahydropicene-2-carboxylate (**IIa**)

A light yellow solid was obtained in 67% yield. HPLC analysis: 97%. m.p. 108–110 °C. ^1^H NMR (400 MHz, DMSO-*d*_6_) *δ* 8.87 (d, *J* = 7.6 Hz, 1H), 8.44 (d, *J* = 8.4 Hz, 1H), 8.29 (d, *J* = 8.8 Hz, 1H), 8.19 (s, 1H), 8.08 (d, *J* = 7.2 Hz, 1H), 8.01 (d, *J* = 8.4 Hz, 2H), 7.91 (t, *J* = 6.0 Hz, 1H), 7.81 (d, *J* = 8.0 Hz, 2H), 7.76 (d, *J* = 7.2 Hz, 2H), 7.72 (t, *J* = 7.2 Hz, 1H), 7.62 − 7.54 (m, 4H), 7.23 (d, *J* = 7.6 Hz, 1H), 5.76 (s, 1H), 5.32 (s, 1H), 5.16 (s, 2H), 4.68 − 4.66 (m, 2H), 4.49 − 4.47 (m, 2H), 4.35 − 4.29 (m, 2H), 3.03 − 2.98 (m, 1H), 2.81 (s, 6H), 2.76 (q, *J* = 6.8 Hz, 2H), 2.57 (d, *J* = 14.0 Hz, 1H), 2.29 (s, 1H), 2.03 − 1.97 (m, 1H), 1.87 (t, *J* = 7.6 Hz, 1H), 1.71 − 1.57 (m, 7H), 1.53 − 1.49 (m, 2H), 1.41 − 1.32 (m, 5H), 1.29 (s, 3H), 1.23 − 1.16 (m, 4H), 1.07 (d, *J* = 14.0 Hz, 1H), 1.02 (s, 3H), 1.00 (s, 3H), 0.96 (s, 3H), 0.93 − 0.90 (m, 4H), 0.86–8.85 (m, 1H), 0.69 − 0.66 (m, 7H). ^13^C NMR (100 MHz, DMSO-*d*_6_) *δ* 199.52, 195.86, 175.95, 172.21, 166.39, 151.78, 142.22, 139.86, 137.44, 137.09, 136.59, 133.55, 130.21, 129.93, 129.79, 129.54, 129.51, 129.16, 128.58, 128.22, 128.16, 125.44, 124.02, 119.58, 115.54, 77.04, 62.75, 61.56, 58.20, 55.39, 54.53, 53.20, 49.09, 47.99, 45.52, 45.28, 43.94, 43.25, 42.67, 39.23, 38.93, 37.59, 37.08, 32.53, 31.87, 30.56, 30.30, 29.31, 28.59, 28.43, 28.07, 27.43, 26.43, 26.15, 23.44, 23.28, 18.75, 17.59, 16.65, 16.47. Chemical Formula: C_67_H_84_N_6_O_10_S; HRMS (ESI-TOF) calculated for C_67_H_84_N_6_O_10_NaS (M + Na)^+^: 1187.5867; observed: 1187.5867 (Figure 37–39 for supplementary file 1).

##### 2–(2-(4-(((*N^2^*-(4-Benzoylbenzoyl)-*N^6^*-((5-(dimethylamino)naphthalen-1-yl)sulphonyl)-*L*-lysyl)oxy)methyl)-1*H*-1,2,3-triazol-1-yl)ethoxy)ethyl 10-hydroxy-2,4a,6a,6b,9,9,12a-heptamethyl-13-oxo-1,2,3,4,4a,5,6,6a,6b,7,8,8a,9,10,11,12,12a,12b,13,14b-icosahydropicene-2-carboxylate (**IIb**)

A white solid was obtained in 57% yield. HPLC analysis: 97%. m.p. 110–114 °C. ^1^H NMR (600 MHz, DMSO-*d*_6_) *δ* 8.86 (d, *J* = 7.2 Hz, 1H), 8.44 (d, *J* = 8.4 Hz, 1H), 8.29 (d, *J* = 8.4 Hz, 1H), 8.08 (d, *J* = 7.8 Hz, 2H), 8.00 (d, *J* = 8.4 Hz, 2H), 7.88 (t, *J* = 5.4 Hz, 1H), 7.81 (d, *J* = 8.4 Hz, 2H), 7.76 (d, *J* = 7.2 Hz, 2H), 7.71 (t, *J* = 7.2 Hz, 1H), 7.61 − 7.55 (m, 4H), 7.23 (d, *J* = 7.8 Hz, 1H), 5.45 (s, 1H), 5.16 (s, 2H), 4.54 (t, *J* = 5.4 Hz, 2H), 4.32 (q, *J* = 7.2 Hz,1H), 4.29 (d, *J* = 4.8 Hz, 1H), 4.20 − 4.17 (m, 1H), 4.11 − 4.07 (m, 1H), 3.83 (t, *J* = 5.4 Hz, 2H), 3.62 − 3.56 (m, 2H), 3.03– 2.99 (m, 1H), 2.81 (s, 6H), 2.76 (q, *J* = 6.0 Hz, 2H), 2.57 (dt, *J* = 13.2, 3.0 Hz, 1H), 2.31 (s, 1H), 2.08 − 2.02 (m, 1H), 2.00 (d, *J* = 8.4 Hz, 1H), 1.78 (d, *J* = 13.2 Hz, 1H), 1.72 − 1.60 (m, 6H), 1.55 − 1.49 (m, 2H), 1.42 − 1.30 (m, 11H), 1.25 − 1.16 (m, 3H), 1.10 (d, *J* = 12.6 Hz, 1H), 1.06 (s, 3H), 1.01 (d, *J* = 3.0 Hz, 6H), 0.96 − 0.90 (m, 5H), 0.70 (d, *J* = 19.8 Hz, 7H). ^13^C NMR (150 MHz, DMSO-*d*_6_) *δ* 199.60, 195.85, 176.14, 172.23, 169.93, 166.43, 151.79, 141.96, 139.87, 137.47, 137.10, 136.60, 133.54, 130.20, 129.91, 129.79, 129.56, 129.52, 129.15, 128.61, 128.22, 128.15, 127.79, 125.46, 124.01, 119.59, 115.54, 77.05, 68.98, 68.77, 63.31, 61.60, 58.18, 54.55, 53.24, 49.79, 48.34, 45.52, 45.31, 43.97, 43.29, 42.65, 40.92, 40.42, 40.28, 40.14, 40.00, 39.86, 39.72, 39.58, 39.23, 38.96, 37.70, 37.09, 32.55, 31.92, 30.78, 30.29, 29.26, 28.68, 28.59, 27.98, 27.42, 26.51, 26.20, 23.41, 23.25, 18.78, 17.60, 16.65, 16.46, 14.56. Chemical Formula: C_69_H_88_N_6_O_11_S; HRMS(ESI-TOF) calculated for C_69_H_88_N_6_O_11_NaS (M + Na)^+^: 1231.6129; observed: 1231.6138 (Figure 40–42 for supplementary file 1).

##### 2–(2-(2–(4-(((*N^2^*-(4-Benzoylbenzoyl)-*N^6^*-((5-(dimethylamino)naphthalen-1-yl)sulphonyl)-*L*-lysyl)oxy)methyl)-1*H*-1,2,3-triazol-1-yl)ethoxy)ethoxy)ethyl 10-hydroxy-2,4a,6a,6b,9,9,12a-heptamethyl-13-oxo-1,2,3,4,4a,5,6,6a,6b,7,8,8a,9,10,11,12,12a,12b,13,14b-icosahydropicene-2-carboxylate (**IIc**)

A light yellow solid was obtained at a yield of 0.04%. HPLC analysis: 97%. m.p. 95–99 °C. ^1^H NMR (600 MHz, DMSO-*d*_6_) *δ* 8.86 (d, *J* = 7.2 Hz, 1H), 8.44 (d, *J* = 9.0 Hz, 1H), 8.29 (d, *J* = 8.4 Hz, 1H), 8.08 (d, *J* = 9.0 Hz, 2H), 8.00 (d, *J* = 7.8 Hz, 2H), 7.88 (t, *J* = 5.4 Hz, 1H), 7.81 (d, *J* = 7.8 Hz, 2H), 7.76 (d, *J* = 7.8 Hz, 2H), 7.71 (t, *J* = 7.8 Hz, 1H), 7.61 − 7.54 (m, 4H), 7.23 (d, *J* = 7.2 Hz, 1H), 5.46 (s, 1H), 5.17 (s, 2H), 4.51 (t, *J* = 5.4 Hz, 2H), 4.32 (t, *J* = 7.2 Hz, 1H), 4.29 (d, *J* = 5.4 Hz, 1H), 4.25 − 4.21 (m, 1H), 4.09 − 4.06 (m, 1H), 3.78 (t, *J* = 5.4 Hz, 2H), 3.56 − 3.54 (m, 2H), 3.52 − 3.48(m, 4H), 3.03 − 2.99 (m, 1H), 2.81 (s, 6H), 2.76 (q, *J* = 6.6 Hz, 2H), 2.57 (dt, *J* = 13.8, 3.0 Hz, 1H), 2.31 (s, 1H), 2.08 − 2.01 (m, 2H), 1.80 (d, *J* = 13.2 Hz, 1H), 1.73 − 1.70 (m, 3H), 1.67 − 1.60 (m, 3H), 1.54 − 1.50 (m, 2H), 1.41 − 1.30 (m,11H), 1.23 − 1.19 (m, 3H), 1.12 (d, *J* = 13.2 Hz, 1H), 1.08 (s, 3H), 1.01 (d, *J* = 3.0 Hz, 6H), 0.95 − 0.90 (m, 5H), 0.70 (d, *J* = 18.0 Hz, 7H). ^13^C NMR (150 MHz, DMSO-*d*_6_) *δ* 199.60, 195.86, 176.17, 172.24, 169.96, 166.43, 151.79, 141.96, 139.87, 137.47, 137.11, 136.59, 133.54, 130.20, 129.90, 129.79, 129.55, 129.52, 129.15, 128.62, 128.22, 128.15, 127.81, 125.51, 124.02, 119.59, 115.55, 77.04, 69.96, 69.94, 69.14, 69.00, 63.44, 61.60, 58.21, 55.39, 54.55, 53.25, 49.84, 48.28, 45.52, 45.30, 43.99, 43.29, 42.64, 40.94, 40.42, 40.28, 40.14, 40.00, 39.86, 39.72, 39.58, 39.23, 38.95, 37.70, 37.09, 32.55, 31.91, 30.85, 30.29, 29.26, 28.66, 28.59, 28.05, 27.43, 26.52, 26.21, 23.44, 23.24, 18.78, 17.60, 16.64, 16.46. Chemical Formula: C_71_H_92_N_6_O_12_S; HRMS (ESI-TOF) calculated for C_71_H_92_N_6_O_12_NaS (M + Na)^+^: 1275.6392; observed: 1275.6400 (Figure 43–45 for supplementary file 1).

##### 2–(2–(2-(2-(4-(((*N*^2^-(4-Benzoylbenzoyl)-*N*^6^-((5-(dimethylamino)naphthalen-1-yl)sulphonyl)-*L*-lysyl)oxy)methyl)-1*H*-1,2,3-triazol-1-yl)ethoxy)ethoxy)ethoxy)ethyl 10-hydroxy-2,4a,6a,6b,9,9,12a-heptamethyl-13-oxo-1,2,3,4,4a,5,6,6a,6b,7,8,8a,9,10,11,12,12a,12b,13,14b-icosahydropicene-2-carboxylate (**IId**)

A light yellow solid was obtained in 21% yield. HPLC analysis: 98%, m.p. 90–93 °C. ^1^H NMR (400 MHz, DMSO-*d*_6_) *δ* 8.86 (d, *J* = 7.2 Hz, 1H), 8.44 (d, *J* = 8.6 Hz, 1H), 8.29 (d, *J* = 8.8 Hz, 1H), 8.10 − 8.07 (m, 2H), 8.00 (d, *J* = 8.4 Hz, 2H), 7.87 (t, *J* = 5.6 Hz, 1H), 7.81 (d, *J* = 8.6 Hz, 2H), 7.77 (s, 1H), 7.75 (d, *J* = 1.2 Hz, 1H), 7.74 − 7.69 (m, 1H), 7.62 − 7.54 (m, 4H), 7.23 (d, *J* = 7.6 Hz, 1H), 5.46 (s, 1H), 5.17 (s, 2H), 4.51 (t, *J* = 5.2 Hz, 2H), 4.35 − 4.22 (m, 3H), 4.12 − 4.06 (m, 1H), 3.79 (t, *J* = 5.6 Hz, 2H), 3.59 (t, *J* = 5.2 Hz, 2H), 3.52 − 3.43 (m, 8H), 3.04 − 2.99 (m, 1H), 2.82 (s, 6H), 2.76 (q, *J* = 6.4 Hz, 2H), 2.57 (d, *J* = 13.2 Hz, 1H), 2.32 (s, 1H), 2.10 − 2.02 (m, 2H), 1.81 (d, *J* = 12.8 Hz, 1H), 1.73 − 1.60 (m, 6H), 1.56 − 1.50 (m, 2H), 1.42 − 1.36 (m, 4H), 1.33 (s, 3H), 1.30 − 1.23 (m, 6H), 1.14 − 1.10 (m, 1H), 1.09 (s, 3H), 1.02 (s, 6H), 0.94 − 0.93 (m, 2H), 0.91 (s, 3H), 0.73 (s, 3H), 0.69 − 0.67 (m, 4H). ^13^C NMR (100 MHz, DMSO-*d*_6_) *δ* 199.55, 195.85, 176.18, 172.23, 169.90, 166.46, 151.80, 141.98, 139.89, 137.48, 137.11, 136.62, 133.52, 130.18, 129.88, 129.79, 129.57, 129.54, 129.14, 128.61, 128.21, 128.14, 127.85, 125.52, 123.99, 119.60, 115.54, 77.07, 70.14, 70.10, 70.07, 69.96, 69.11, 69.00, 63.45, 61.62, 58.22, 54.57, 53.27, 49.88, 48.26, 45.51, 45.30, 43.98, 43.29, 42.65, 40.98, 39.23, 38.99, 37.71, 37.11, 32.57, 31.91, 30.88, 30.30, 29.26, 28.66, 28.59, 28.06, 27.42, 26.52, 26.24, 23.44, 23.23, 18.78, 17.60, 16.62, 16.44. Chemical Formula: C_73_H_96_N_6_O_13_S; HRMS (ESI-TOF) calculated for C_73_H_97_N_6_O_13_S (M + H)^+^: 1297.6834; observed: 1297.6835 (Figure 46–48 for supplementary file 1).

#### General procedure for synthesis of compounds 13a-13d

Compound **12** (1 eq) was dissolved in THF (60 ml). t-BuOK (1 eq), 18-crown-6 (1 eq), and the corresponding bromoethanol (1 eq) were added sequentially in an ice bath. After 30 min, the reaction system was heated to room temperature for 2 days. The reaction mixture was then poured into water, and the products were extracted with DCM. The extracted material was washed with brine and dried over Na_2_SO_4_. After removing the solvent under vacuum, the residue was purified (silica gel, PE/EAC = 2/1, v/v) to obtain compounds **13a-13d**.

##### (2-Hydroxy-4-methoxyphenyl)(2–(2-hydroxyethoxy)-4-methoxyphenyl)metha none (13a)

A white solid was obtained in 30% yield. m.p. 112–115 °C. ^1^H NMR (600 MHz, CDCl_3_) *δ* 12.67 (s, 1H), 7.36 (d, *J* = 8.9 Hz, 1H), 7.29 (d, *J* = 8.4 Hz, 1H), 6.59 (dd, *J* = 8.4, 2.2 Hz, 1H), 6.55 (d, *J* = 2.1 Hz, 1H), 6. 48 (d, *J* = 2.4 Hz, 1H), 6.37 (dd, *J* = 8.9, 2.4 Hz, 1H), 4.12 (t, *J* = 4. 38 Hz, 2H), 3.86 (s, 3H), 3.84 (s, 3H), 3.80 − 3.76 (m, 2H), 2.67 (t, *J* = 6.7 Hz, 1H). ^13^C NMR (151 MHz, CDCl_3_) *δ* 199.1, 166.3, 166.0, 163.0, 135.3, 131.5, 121.2, 114.3, 107.4, 105.5, 100.9, 100.8, 71.0, 61. 1, 55.7, 55.6. Chemical Formula: C_17_H_18_O_6_; HRMS (ESI-TOF) calculated for C_17_H_18_O_6_Na (M *+* Na)^+^: 341.1001; observed: 341.1010 (Figure 49–51 for supplementary file 1).

##### (2-Hydroxy-4-methoxyphenyl)(2–(2–(2-hydroxyethoxy)ethoxy)-4-methoxyphenyl)methanone (13b)

A light yellow liquid was obtained in 29% yield. ^1^H-NMR (600 MHz, CDCl_3_) *δ* 12.71 (s, 1H), 7.31 (d, *J* = 8.9 Hz, 1H), 7.26 (d, *J* = 8.3 Hz, 1H), 6.58 (dd, *J* = 8.4, 2.2 Hz, 1H), 6.54 (d, *J* = 2.2 Hz, 1H), 6.46 (d, *J* = 2.4 Hz, 1H), 6.35 (dd, *J* = 9.0, 2.5 Hz, 1H), 4.11 − 4.09 (m, 2H), 3.85 (s, 3H), 3.84 (s, 3H), 3.68 (dd, *J* = 5.3, 4.1 Hz, 2H), 3.60 − 3.58 (m, 2H), 3.46 − 3.44 (m, 2H), 2.10 (s, 1H). ^13^C-NMR (151 MHz, CDCl_3_) *δ* 199.37, 166.08, 165.67, 162.74, 157.34, 135.57, 130.72, 121.36, 114.59, 107.22, 105.23, 100.54, 100.02, 72.56, 69.13, 68.50, 61.70, 55.61, 55.56. Chemical Formula: C_19_H_22_O_7_; HRMS (ESI-TOF) calculated for C_19_H_23_O_7_ (M + H)^+^: 363.1445; observed: 363.1432 (Figure 52–54 for supplementary file 1).

##### (2-Hydroxy-4-methoxyphenyl)(2–(2–(2–(2-hydroxyethoxy)ethoxy)ethoxy)-4-methoxyphenyl)methanone (13c)

A light yellow liquid was obtained in 23% yield. ^1^H-NMR (600 MHz, CDCl_3_) *δ* 12.75 (s, 1H), 7.31 (d, *J* = 8.9 Hz, 1H), 7.27 (d, *J* = 8.4 Hz, 1H), 6.57 (dd, *J* = 8.4, 2.3 Hz, 1H), 6.54 (d, *J* = 2.2 Hz, 1H), 6.46 (d, *J* = 2.5 Hz, 1H), 6.35 (dd, *J* = 8.9, 2.5 Hz, 1H), 4.09 (t, *J* = 4.9 Hz, 2H), 3.85 (d, *J* = 5.9 Hz, 6H), 3.71 − 3.68 (m, 3H), 3.66 (t, *J* = 4.9 Hz, 2H), 3.63 (ddd, *J* = 5.8, 3.3, 1.2 Hz, 2H), 3.60 − 3.57 (m, 4H), 3.50 (m, *J* = 7.7, 6.0, 4.5, 3.4, 1.8 Hz, 4H). ^13^C-NMR (151 MHz, CDCl_3_) *δ* 165.97, 165.56, 162.77, 157.35, 135.62, 130.63, 121.32, 114.62, 107.23, 105.31, 100.46, 99.83, 72.51, 70.81, 70.49, 70.46, 70.26, 69.23, 68.63, 61.69, 55.59, 55.55. Chemical Formula: C_21_H_26_O_8_; HRMS (ESI-TOF) calculated for C_21_H_27_O_8_ (M + H)^+^: 407.1707; observed: 407.1696 (Figure 55–57 for supplementary file 1).

##### (2-Hydroxy-4-methoxyphenyl)(2–(2-(2-(2-(2-hydroxyethoxy)ethoxy)ethoxy)ethoxy)-4-methoxyphenyl)methanone (13d)

A light yellow liquid was obtained in 23% yield. ^1^H-NMR (600 MHz, CDCl_3_) *δ* 12.75 (s, 1H), 7.29 (dd, *J* = 23.5, 8.7 Hz, 2H), 6.59 − 6.54 (m, 2H), 6.46 (d, *J* = 2.5 Hz, 1H), 6.35 (dd, *J* = 8.9, 2.5 Hz, 1H), 4.11 − 4.08 (m, 2H), 3.85 (d, *J* = 5.9 Hz, 6H), 3.67 (q, *J* = 4.4 Hz, 4H), 3.54 − 3.50 (m, 4H), 3.49 (dt, *J* = 6.0, 1.8 Hz, 2H). ^13^C-NMR (151 MHz, CDCl_3_) *δ* 199.38, 166.00, 165.57, 162.77, 157.32, 135.61, 130.64, 121.35, 114.62, 107.26, 105.29, 100.45, 99.91, 72.43, 70.90, 70.34, 69.29, 68.70, 61.72, 55.60, 55.55. Chemical Formula: C_23_H_30_O_9_; HRMS (ESI-TOF) calculated for C_23_H_30_O_9_Na (M + Na)^+^: 473.1788; observed: 473.1797 (Figure 58–60 for supplementary file 1).

#### General procedure for synthesis of compounds 14a-14d

The compounds **13a-13d** (1 eq) were dissolved in THF (30 ml). t-BuOK (1 eq), 18-crown-6 (1 eq), and propargyl bromide (1.2 eq) were added sequentially at r.t. After stirring overnight, the reaction mixture was poured into water, and the products were extracted using DCM. The extracted material was washed with brine and dried over Na_2_SO_4_. After removing the solvent under vacuum, the residue was purified (silica gel, PE/EAC = 1/4, v/v) to obtain compounds **14a**–**14d.**

(2-(2-Hydroxyethoxy)-4-methoxyphenyl)(4-methoxy-2-(prop-2-yn-1-yloxy)phenyl)methanone (**14a**)

A white solid was obtained in 63% yield. m.p. 99–104 °C. ^1^H NMR (600 MHz, CDCl_3_) *δ* 7.54 (d, *J* = 8.6 Hz, 1H), 7.50 (d, *J* = 8.5 Hz, 1H), 6.62 (d, *J* = 2.2 Hz, 1H), 6.59 (dd, *J* = 8.5, 2.2 Hz, 1H), 6.53 (dd, *J* = 8.6, 2.3 Hz, 1H), 6.41 (d, *J* = 2.2 Hz, 1H), 4.54 (d, *J* = 2.4 Hz, 2H), 4.0 1 − 3.97 (m, 2H), 3.85 (d, *J* = 5.2 Hz, 6H), 3.62 (q, *J* = 5.0 Hz, 2H), 2.46 (t, *J* = 2.4 Hz, 1H), 2.30 (t, *J* = 7.0 Hz, 1H). 13 C NMR (151 M Hz, CDCl_3_) *δ* 192.99, 163.80, 163.16, 159.46, 157.58, 132.91, 131.99, 124.84, 123.54, 105.79, 105.33, 100.31, 99.80, 78.13, 75.96, 70.40, 61. 13, 56.46, 55.64, 55.53. Chemical Formula: C_20_H_20_O_6_; HRMS (ESI-TOF) calculated for C_20_H_21_O_6_ (M *+* H)^+^: 357.1339; observed: 357.1317 (Figure 61–63 for supplementary file 1).

##### (2–(2–(2-Hydroxyethoxy)ethoxy)-4-methoxyphenyl)(4-methoxy-2-(prop-2-yn-1-yloxy)phenyl)methanone (14b)

A light yellow liquid was obtained in 23% yield. ^1^H-NMR (600 MHz, CDCl_3_) *δ* 7.54 (dd, *J* = 12.1, 8.5 Hz, 2H), 6.60 (d, *J* = 2.3 Hz, 1H), 6.57 (dd, *J* = 8.5, 2.3 Hz, 1H), 6.52 (dd, *J* = 8.6, 2.3 Hz, 1H), 6.40 (d, *J* = 2.3 Hz, 1H), 4.52 (d, *J* = 2.4 Hz, 2H), 4.01 − 3.98 (m, 2H), 3.85 (d, *J* = 7.3 Hz, 6H), 3.62 (dd, *J* = 5.2, 4.0 Hz, 2H), 3.49 (t, *J* = 4.7 Hz, 2H), 3.40 − 3.37 (m, 2H), 2.46 (t, *J* = 2.4 Hz, 1H). ^13^C-NMR (151 MHz, CDCl_3_) *δ* 192.73, 163.55, 163.10, 159.27, 157.99, 132.53, 132.33, 124.83, 123.94, 105.64, 105.01, 100.27, 99.30, 78.40, 75.77, 72.49, 69.19, 68.25, 61.70, 56.52, 55.56, 55.49. Chemical Formula: C_22_H_24_O_7_; HRMS (ESI-TOF) calculated for C_22_H_25_O_7_ (M + H)^+^: 401.1601; observed: 401.1590 (Figure 64– 66 for supplementary file 1).

##### (2–(2–(2–(2-Hydroxyethoxy)ethoxy)ethoxy)-4-methoxyphenyl)(4-methoxy-2-(prop-2-yn-1-yloxy)phenyl)methanone (14c)

A light yellow liquid was obtained in 51% yield. ^1^H-NMR (600 MHz, CDCl_3_) *δ* 7.57 (d, *J* = 8.5 Hz, 1H), 7.53 (d, *J* = 8.5 Hz, 1H), 6.59 (d, *J* = 2.3 Hz, 1H), 6.54 (ddd, *J* = 18.5, 8.5, 2.3 Hz, 2H), 6.39 (d, *J* = 2.3 Hz, 1H), 4.52 (d, *J* = 2.4 Hz, 2H), 3.97 (t, *J* = 4.9 Hz, 2H), 3.85 (d, *J* = 7.2 Hz, 6H), 3.72 − 3.68 (m, 2H), 3.64 (dd, *J* = 6.1, 3.4 Hz, 2H), 3.63 − 3.57 (m, 4H), 3.56 − 3.52 (m, 2H), 3.45 − 3.39 (m, 4H), 2.50 (t, *J* = 2.4 Hz, 1H). ^13^C-NMR (151 MHz, CDCl_3_) *δ* 192.65, 163.59, 163.06, 159.28, 158.02, 132.32, 132.25, 124.94, 123.96, 105.54, 105.05, 100.12, 99.06, 78.44, 75.89, 72.51, 70.71, 70.51, 70.46, 70.27, 69.26, 68.18, 61.69, 56.44, 55.54, 55.48. Chemical Formula: C_24_H_28_O_8_; HRMS (ESI-TOF) calculated for C_24_H_29_O_8_ (M + H)^+^: 445.1863; observed: 445.1857 (Figure 67–69 for supplementary file 1).

##### (2-(2-(2-(2-(2-Hydroxyethoxy)ethoxy)ethoxy)ethoxy)-4-methoxyphenyl)(4-methoxy-2-(prop-2-yn-1-yloxy)phenyl)methanone (14d)

A light yellow liquid was obtained in 52% yield. ^1^H-NMR (600 MHz, CDCl_3_) *δ* 7.55 (dd, *J* = 18.9, 8.5 Hz, 2H), 6.60 (d, *J* = 2.2 Hz, 1H), 6.54 (ddd, *J* = 19.7, 8.5, 2.3 Hz, 2H), 6.40 (d, *J* = 2.3 Hz, 1H), 4.52 (d, *J* = 2.4 Hz, 2H), 3.98 (t, *J* = 4.8 Hz, 2H), 3.85 (d, *J* = 7.8 Hz, 6H), 3.70 (t, *J* = 4.6 Hz, 2H), 3.56 (q, *J* = 5.0 Hz, 4H), 3.48 − 3.40 (m, 4H), 2.48 (t, *J* = 2.4 Hz, 1H). ^13^C-NMR (151 MHz, CDCl_3_) *δ* 163.57, 163.07, 159.24, 158.05, 132.40, 132.30, 124.87, 123.95, 105.55, 105.03, 100.22, 99.18, 78.43, 75.82, 72.47, 70.83, 70.35, 69.33, 68.28, 61.72, 56.48, 55.55, 55.48. Chemical Formula: C_26_H_32_O_9_; HRMS (ESI-TOF) calculated for C_26_H_33_O_9_ (M + H)^+^: 489.2128; observed: 489.2104 (Figure 70–72 for supplementary file 1).

#### General procedure for synthesis of compounds 15a-15d

The compounds **14a-14d** (1 eq) and DIPEA (5 eq) were dissolved in DCM (30 ml). Ethyl sulphonyl chloride (2 eq) was added dropwise at r.t. After stirring overnight, the reaction mixture was poured into water, and the products were extracted using DCM. The extracted material was washed with brine and dried over Na_2_SO_4_. After removing the solvent under vacuum, the residue was purified (silica gel, PE/EAC = 1/7, v/v) to obtain compounds **15a**–**15d**.

##### 2–(5-Methoxy-2–(4-methoxy-2-(prop-2-yn-1-yloxy)benzoyl)phenoxy)ethylethanesulfonate (15a)

A light yellow solid was obtained in 90% yield. m.p. 95–100 °C. ^1^H NMR (600 MHz, CDCl_3_) *δ* 7.54 (dd, *J* = 8.5, 4.3 Hz, 2H), 6.60 − 6.55 (m, 3H), 6.39 (d, *J* = 2.3 Hz, 1H), 4.52 (d, *J* = 2.5 Hz, 2H), 4.13 (ddd, *J* = 22.9, 6.2, 3.8 Hz, 4H), 3.86 (d, *J* = 8.5 Hz, 6H), 3.01 (q, *J* = 7.4 Hz, 2H), 2.4 7 (t, *J* = 2.4 Hz, 1H), 1.31 (t, *J* = 7.4 Hz, 3H). ^13^C NMR (151 MHz, CDCl_3_) *δ* 192.35, 163.47, 163.39, 158.41, 157.93, 132.43, 132.33, 124. 29, 105.90, 105.66, 99.97, 99.61, 78.27, 75.91, 67.41, 66.50, 56.32, 55.5 7, 44.84, 8.06. Chemical Formula: C_22_H_24_O_8_S; HRMS (ESI-TOF) calculated for C_22_H_25_O_8_S (M *+* H)^+^: 449.1271; observed: 449.1248 (Figure 73–75 for supplementary file 1).

##### 2–(2-(5-Methoxy-2–(4-methoxy-2-(prop-2-yn-1-yloxy)benzoyl)phenoxy)ethoxy)ethyl ethanesulfonate (15b)

A light yellow liquid was obtained in 72% yield. ^1^H-NMR (600 MHz, CDCl_3_) *δ* 7.54 (dd, *J* = 11.9, 8.5 Hz, 2H), 6.60 (d, *J* = 2.3 Hz, 1H), 6.57 (dd, *J* = 8.5, 2.3 Hz, 1H), 6.54 (dd, *J* = 8.6, 2.3 Hz, 1H), 6.39 (d, *J* = 2.3 Hz, 1H), 4.52 (d, *J* = 2.4 Hz, 2H), 4.22 − 4.20 (m, 2H), 3.99 − 3.97 (m, 2H), 3.85 (d, *J* = 8.2 Hz, 6H), 3.48 (td, *J* = 4.6, 1.9 Hz, 4H), 3.12 (q, *J* = 7.4 Hz, 2H), 2.47 (t, *J* = 2.4 Hz, 1H), 1.36 (t, *J* = 7.4 Hz, 3H). ^13^C-NMR (151 MHz, CDCl_3_) *δ* 163.54, 163.19, 159.08, 158.03, 132.41, 124.72, 124.00, 105.56, 105.04, 100.16, 99.17, 78.36, 75.86, 69.45, 69.27, 68.91, 68.43, 56.43, 55.61, 55.51, 45.00, 8.10. Chemical Formula: C_24_H_28_O_9_S; HRMS (ESI-TOF) calculated for C_24_H_29_O_9_S (M + H)^+^: 493.1533; observed: 493.1523 (Figure 76–78 for supplementary file 1).

##### 2–(2-(2–(5-Methoxy-2–(4-methoxy-2-(prop-2-yn-1-yloxy)benzoyl)phenoxy)ethoxy)ethoxy)ethyl ethanesulfonate (15c)

A light yellow liquid was obtained in 80% yield. ^1^H-NMR (600 MHz, CDCl_3_) *δ* 7.57 (d, *J* = 8.6 Hz, 1H), 7.52 (d, *J* = 8.5 Hz, 1H), 6.59 (d, *J* = 2.3 Hz, 1H), 6.54 (ddd, *J* = 18.7, 8.5, 2.3 Hz, 2H), 6.39 (d, *J* = 2.3 Hz, 1H), 4.51 (d, *J* = 2.4 Hz, 2H), 4.35 − 4.32 (m, 2H), 3.97 (t, *J* = 4.9 Hz, 2H), 3.85 (d, *J* = 6.8 Hz, 6H), 3.75 − 3.72 (m, 2H), 3.65 − 3.62 (m, 2H), 3.62 − 3.59 (m, 2H), 3.54 − 3.51 (m, 2H), 3.44 − 3.39 (m, 4H), 3.17 (q, *J* = 7.4 Hz, 2H), 2.49 (t, *J* = 2.4 Hz, 1H), 1.40 (t, *J* = 7.4 Hz, 3H). ^13^C-NMR (151 MHz, CDCl_3_) *δ* 192.56, 163.58, 163.06, 159.29, 132.33, 132.21, 123.95, 105.56, 105.02, 100.14, 99.07, 78.44, 75.87, 70.74, 70.64, 70.55, 70.47, 69.28, 69.07, 68.92, 68.20, 56.47, 55.55, 55.48, 45.02, 8.16. Chemical Formula: C_26_H_32_O_10_S; HRMS (ESI-TOF) calculated for C_26_H_33_O_10_S (M + H)^+^: 537.1795; observed:537.1789 (Figure 79–81 for supplementary file 1).

##### 2–(2-(2–(5-Methoxy-2–(4-methoxy-2-(prop-2-yn-1-yloxy)benzoyl)phenoxy)ethoxy)ethoxy)ethoxy)ethyl ethanesulfonate (15d)

A light yellow liquid was obtained in 51% yield. ^1^H-NMR (600 MHz, CDCl_3_) *δ* 7.56 (d, *J* = 8.6 Hz, 1H), 7.52 (d, *J* = 8.5 Hz, 1H), 6.59 (d, *J* = 2.3 Hz, 1H), 6.56 (dd, *J* = 8.5, 2.3 Hz, 1H), 6.53 (dd, *J* = 8.6, 2.3 Hz, 1H), 6.39 (d, *J* = 2.3 Hz, 1H), 4.51 (d, *J* = 2.4 Hz, 2H), 4.34 − 4.31 (m, 2H), 3.97 (t, *J* = 4.8 Hz, 2H), 3.85 (d, *J* = 7.1 Hz, 6H), 3.73 − 3.69 (m, 2H), 3.57 − 3.53 (m, 2H), 3.45 − 3.38 (m, 4H), 3.16 (q, *J* = 7.4 Hz, 2H), 2.48 (t, *J* = 2.4 Hz, 1H), 1.39 (t, *J* = 7.4 Hz, 3H). ^13^C-NMR (151 MHz, CDCl_3_) *δ* 192.54, 163.56, 163.09, 159.24, 158.03, 132.36, 132.26, 124.87, 123.95, 105.58, 104.99, 100.16, 99.14, 78.42, 75.85, 70.71, 70.63, 69.35, 69.05, 68.89, 68.28, 56.48, 55.57, 55.49, 44.99, 8.14. Chemical Formula: C_28_H_37_O_11_S; HRMS (ESI-TOF) calculated for C_28_H_36_O_11_S (M + H)^+^: 581.2057; observed: 581.2033 (Figure 82–84 for supplementary file 1).

#### General procedure for synthesis of compounds 16a-16d

The compound 18*β*-GA (1.3 eq) and K_2_CO_3_ (2.8 eq) were dissolved in DMF (40 ml). The corresponding compounds **15a**–**15d** (1 eq) were added at r.t. After stirring overnight, the reaction mixture was poured into water, and the products were extracted using DCM. The extracted material was washed with brine and dried over Na_2_SO_4_. After removing the solvent under vacuum, the residue was purified (silica gel, PE/EAC = 3/1, v/v) to obtain compounds **16a**–**16d**.

##### 2–(5-Methoxy-2–(4-methoxy-2-(prop-2-yn-1-yloxy)benzoyl)phenoxy)ethyl (2*S*,3*S*,4a*S*,6a*S*,6b*R*,10*S*,12a*S*)-10-hydroxy-2,4a,6a,6b,9,9,12a-heptamethyl-13-oxo-1,2,3,4,4a,5,6,6a,6b,7,8,8a,9,10,11,12,12a,12b,13,14b-icosahydropicene-3-carboxylate (16a)

A white solid was obtained in 66% yield. m.p. 181–184 °C. ^1^H NMR (600 MHz, CDCl_3_) *δ* 7.57 (d, *J* = 8.6 Hz, 1H), 7.51 (d, *J* = 8.5 Hz, 1H), 6.58 (d, *J* = 2.3 Hz, 1H), 6.55 (ddd, *J* = 8.5, 4.5, 2.2 Hz, 2H), 6.38 (d, *J* = 2.3 Hz, 1H), 5.61 (s, 1H), 4.49 (d, *J* = 2.4 Hz, 2H), 4.04 − 3.94 (m, 4H), 3.86 (s, 3H), 3.85 (s, 3H), 3.22 (dt, *J* = 11.1, 5.4 Hz, 1H), 2.78 (dt, *J* = 13.5, 3.6 Hz, 1H), 2.46 (t, *J* = 2.4 Hz, 1H), 2.33 (s, 1H), 2.07 − 2.04 (m, 1H), 1.99 (td, *J* = 13.6, 4.5 Hz, 1H), 1.89 (dq, *J* = 13.4, 3.2 Hz, 1H), 1.86 − 1.77 (m, 2H), 1.62 (m, *J* = 16.7, 12.9, 10.2, 3.1 Hz, 4H), 1.48 − 1.38 (m, 2H), 1.35 (s, 3H), 1.34 − 1.30 (m, 2H), 1.29 − 1.24 (m, 1H), 1.22 − 1.15 (m, 2H), 1.13 (s, 3H), 1.11 (s, 3H), 1. 06 (s, 3H), 1.00 (s, 3H), 1.00 − 0.94 (m, 2H), 0.80 (s, 3H), 0.75 (s, 3H), 0.69 (dd, *J* = 12.0, 1.8 Hz, 1H). ^13^C NMR (151 MHz, CDCl_3_) *δ* 200.09, 192.54, 176.10, 169.07, 163.54, 163.21, 158.99, 157.82, 132.24, 132.16, 128.55, 124.85, 124.29, 105.75, 105.51, 100.03, 99.46, 78.77, 78.36, 75.76, 66.38, 62.34, 61.82, 56.36, 55.59, 55.52, 54.94, 48.15, 45.37, 43.90, 43.19, 40.97, 39.14, 37.74, 37.09, 32.76, 31.78, 30.98, 28.48, 28.23, 28.10, 27.32, 26.44, 26.37, 23.41, 18.68, 17.49, 16.36, 15.59. Chemical Formula: C_50_H_64_O_9_; HRMS (ESI-TOF) calculated for C_50_H_64_O_9_Na (M *+*Na)^+^: 831.4448; observed: 831.4465 (Figure 85–87 for supplementary file 1).

##### 2–(2-(5-Methoxy-2–(4-methoxy-2-(prop-2-yn-1-yloxy)benzoyl)phenoxy)ethoxy)ethyl(2S,4aS,6aS,6bR,8aR,10S,12aS,12bR,14bR)-10-hydroxy-2,4a,6a,6b,9,9,12a-heptamethyl-13-oxo-1,2,3,4,4a,5,6,6a,6b,7,8,8a,9,10,11,12,12a,12b,13,14b-icosahydropicene-2-carboxylate (16b)

A light yellow liquid was obtained in 47% yield. ^1^H-NMR (600 MHz, CDCl_3_) *δ* 7.58 (d, *J* = 8.5 Hz, 1H), 7.53 (d, *J* = 8.5 Hz, 1H), 6.60 (d, *J* = 2.2 Hz, 1H), 6.57 (dd, *J* = 8.5, 2.2 Hz, 1H), 6.52 (dd, *J* = 8.6, 2.2 Hz, 1H), 6.43 (d, *J* = 2.3 Hz, 1H), 5.64 (s, 1H), 4.51 (d, *J* = 2.4 Hz, 2H), 4.20 (ddd, *J* = 12.0, 6.2, 3.4 Hz, 1H), 4.05 (ddd, *J* = 12.0, 5.8, 3.3 Hz, 1H), 4.00 (t, *J* = 4.7 Hz, 2H), 3.86 (s, 3H), 3.85 (s, 3H), 3.48 − 3.37 (m, 4H), 3.22 (dt, *J* = 10.6, 4.9 Hz, 1H), 2.77 (dt, *J* = 13.5, 3.6 Hz, 1H), 2.47 (t, *J* = 2.5 Hz, 1H), 2.33 (s, 1H), 2.08 (dd, *J* = 14.2, 4.1 Hz, 1H), 2.04 − 1.94 (m, 2H), 1.90 (dt, *J* = 13.7, 3.5 Hz, 1H), 1.84 (d, *J* = 4.7 Hz, 1H), 1.63 (dt, *J* = 26.7, 13.1 Hz, 5H), 1.48 − 1.37 (m, 4H), 1.35 (s, 3H), 1.35 − 1.31 (m, 1H), 1.17 (d, *J* = 14.0 Hz, 1H), 1.13 (s, 6H), 1.10 (s, 3H), 1.00 (s, 4H), 0.96 (dd, *J* = 13.1, 4.2 Hz, 1H), 0.81 (s, 3H), 0.78 (s, 3H), 0.70 (d, *J* = 13.1 Hz, 1H). ^13^C-NMR (151 MHz, CDCl_3_) *δ* 200.18, 192.59, 176.33, 169.4, 163.66, 163.06, 159.34, 158.07, 132.28, 132.25, 128.37, 125.07, 123.88, 105.50, 105.24, 100.15, 98.92, 78.74, 78.45, 75.81, 69.43, 69.21, 68.38, 63.23, 61.83, 56.49, 55.57, 55.47, 54.94, 48.31, 45.37, 44.02, 43.17, 41.14, 39.14, 37.62, 37.06, 32.75, 31.79, 31.17, 28.55, 28.23, 28.10, 27.29, 26.50, 26.40, 23.37, 18.66, 17.49, 16.36, 15.60. Chemical Formula: C_52_H_68_O_10_; HRMS (ESI-TOF) calculated for C_52_H_68_O_10_Na (M + Na)^+^: 875.4710; observed: 875.4734 (Figure 88–90 for supplementary file 1).

##### 2–(2-(2–(5-Methoxy-2–(4-methoxy-2-(prop-2-yn-1-yloxy)benzoyl)phenoxy)ethoxy)ethoxy)ethyl(2*S*,4a*S*,6a*S*,6b*R*,8a*R*,10*S*,12a*S*,12b*R*,14b*R*)-10-hydroxy-2,4a,6a,6b,9,9,12a-heptamethyl-13-oxo-1,2,3,4,4a,5,6,6a,6b,7,8,8a,9,10,11,12,12a,12b,13,14b-icosahydropicene-2-carboxylate (16c)

A light yellow liquid was obtained in 47% yield. ^1^H-NMR (600 MHz, CDCl_3_) *δ* 7.58 (d, *J* = 8.5 Hz, 1H), 7.52 (d, *J* = 8.5 Hz, 1H), 6.59 (d, *J* = 2.4 Hz, 1H), 6.56 (dd, *J* = 8.5, 2.3 Hz, 1H), 6.53 (dd, *J* = 8.6, 2.3 Hz, 1H), 6.39 (d, *J* = 2.3 Hz, 1H), 5.64 (s, 1H), 4.51 (d, *J* = 2.5 Hz, 2H), 4.28 (ddd, *J* = 11.9, 5.7, 4.3 Hz, 1H), 4.22 − 4.17 (m, 1H), 3.96 (t, *J* = 5.0 Hz, 2H), 3.85 (s, 3H), 3.84 (s, 3H), 3.65 (ddd, *J* = 6.1, 4.2, 2.1 Hz, 2H), 3.55 − 3.52 (m, 2H), 3.41 (q, *J* = 5.3 Hz, 4H), 3.22 (dd, *J* = 11.3, 5.0 Hz, 1H), 2.78 (dt, *J* = 13.5, 3.6 Hz, 1H), 2.48 (t, *J* = 2.4 Hz, 1H), 2.33 (s, 1H), 2.12 (dd, *J* = 13.5, 2.6 Hz, 1H), 2.04 − 1.96 (m, 2H), 1.94 − 1.89 (m, 1H), 1.82 (td, *J* = 13.8, 4.7 Hz, 1H), 1.73 (d, *J* = 17.2 Hz, 1H), 1.68 − 1.58 (m, 5H), 1.47 − 1.38 (m, 3H), 1.36 (s, 3H), 1.33 − 1.30 (m, 2H), 1.18 (dt, *J* = 13.8, 3.5 Hz, 1H), 1.15 (s, 3H), 1.13 (s, 3H), 1.12 (s, 3H), 1.00 (s, 3H), 0.96 (dd, *J* = 13.1, 4.2 Hz, 1H), 0.80 (s, 3H), 0.80 (s, 3H), 0.70 (d, *J* = 10.0 Hz, 1H). ^13^C-NMR (151 MHz, CDCl_3_) *δ* 200.17, 192.61, 176.35, 169.30, 163.60, 163.04, 159.34, 158.04, 132.34, 132.20, 128.51, 125.05, 123.99, 105.54, 105.01, 100.17, 99.10, 78.76, 78.46, 75.84, 70.74, 70.42, 69.30, 69.17, 68.10, 63.27, 61.83, 56.49, 55.54, 55.47, 54.94, 48.23, 45.38, 44.02, 43.19, 41.10, 39.14, 37.70, 37.09, 32.76, 31.81, 31.16, 28.55, 28.29, 28.10, 27.30, 26.49, 26.42, 23.42, 18.68, 17.49, 16.38, 15.59. Chemical Formula: C_54_H_72_O_11_; HRMS (ESI-TOF) calculated for C_54_H_73_O_11_ (M + H)^+^: 897.5154; observed: 897.5120 (Figure 91–93 for supplementary file 1).

##### 2–(2–(2-(2-(5-Methoxy-2–(4-methoxy-2-(prop-2-yn-1-yloxy)benzoyl)phenoxy)ethoxy)ethoxy)ethoxy)ethyl(2*S*,4a*S*,6a*S*,6b*R*,8a*R*,10*S*,12a*S*,12b*R*,14b*R*)-10-hydroxy-2,4a,6a,6b,9,9,12a-heptamethyl-13-oxo-1,2,3,4,4a,5,6,6a,6b,7,8,8a,9,10,11,12,12a,12b,13,14b-icosahydropicene-2-carboxylatev (16d)

A light yellow liquid was obtained in 44% yield. ^1^H-NMR (600 MHz, CDCl_3_) *δ* 7.58 (d, *J* = 8.6 Hz, 1H), 7.52 (d, *J* = 8.5 Hz, 1H), 6.59 (d, *J* = 2.2 Hz, 1H), 6.56 (dd, *J* = 8.5, 2.3 Hz, 1H), 6.53 (dd, *J* = 8.6, 2.3 Hz, 1H), 6.39 (d, *J* = 2.2 Hz, 1H), 5.65 (s, 1H), 4.51 (d, *J* = 2.5 Hz, 2H), 4.30 (ddd, *J* = 12.0, 5.7, 4.1 Hz, 1H), 4.20 (ddd, *J* = 11.9, 5.5, 3.9 Hz, 1H), 3.96 (t, *J* = 5.0 Hz, 2H), 3.85 (s, 3H), 3.84 (s, 3H), 3.68 (ddd, *J* = 6.0, 4.0, 2.1 Hz, 2H), 3.65 − 3.59 (m, 4H), 3.54 − 3.50 (m, 2H), 3.43 − 3.37 (m, 4H), 3.22 (dd, *J* = 11.4, 4.9 Hz, 1H), 2.77 (dt, *J* = 13.5, 3.5 Hz, 1H), 2.50 (t, *J* = 2.4 Hz, 1H), 2.33 (s, 1H), 2.13 (dd, *J* = 14.4, 3.5 Hz, 1H), 2.03 − 1.97 (m, 2H), 1.94 − 1.90 (m, 1H), 1.82 (td, *J* = 13.7, 4.7 Hz, 1H), 1.67 − 1.58 (m, 5H), 1.47 − 1.38 (m, 3H), 1.36 (s, 3H), 1.34 − 1.30 (m, 2H), 1.20 − 1.16 (m, 1H), 1.15 (s, 3H), 1.13 (s, 3H), 1.12 (s, 3H), 1.00 (s, 5H), 0.80 (s, 6H), 0.69 (d, *J* = 11.6 Hz, 1H). ^13^C-NMR (151 MHz, CDCl_3_) *δ* 200.15, 192.61, 176.35, 169.32, 163.59, 163.04, 159.31, 158.01, 132.31, 132.15, 128.47, 124.98, 123.94, 105.53, 105.03, 100.09, 99.01, 78.68, 78.43, 75.89, 70.68, 70.50, 70.41, 69.22, 69.16, 68.07, 63.27, 61.80, 56.44, 55.52, 55.45, 54.92, 48.20, 45.36, 44.00, 43.17, 41.08, 39.11, 37.68, 37.05, 32.73, 31.79, 31.13, 28.53, 28.26, 28.09, 27.26, 26.46, 26.40, 23.40, 18.66, 17.46, 16.35, 15.59. Chemical Formula: C_56_H_76_O_12_; HRMS (ESI-TOF) calculated for C_56_H_76_O_12_Na (M + Na)^+^: 963.5235; observed: 963.5270 (Figure 9 4–96 for supplementary file 1).

#### General procedure for synthesis of compounds 17a-17d

The compounds **16a**–**16d** (1 eq) and sodium ascorbate (0.07 eq) and CuSO_4_·5H_2_O (0.06 eq) were dissolved in a mixed solvent of THF and water (V/V = 1/1, 30 ml). Subsequently, 3-azidopropan-1-amine (1.2 eq) was added at r.t. After stirring overnight, the reaction mixture was poured into water, and the products were extracted using DCM. The extracted material was washed with brine and dried over Na_2_SO_4_. After removing the solvent under vacuum, the residue was purified (silica gel, MeOH/DCM = 1/10, v/v) to give crude compounds **17a**–**17d**.

#### General procedure for synthesis of compounds IIIa-IIId

The compounds **17a**–**17d** (1 eq) and Et_3_N (1.4 eq) were dissolved in a mixed solvent of MeOH and DCM (v/v = 1/1, 30 ml). Dansyl chloride (1.4 eq) was then added in an ice bath. After 30 min, the reaction was incubated at r.t. overnight. After the reaction was complete, the reaction mixture was poured into water, and the products were extracted using DCM. The extracted material was washed with brine and dried over Na_2_SO_4_. After removal of the solvent in a vacuum, the residue was purified (silica gel, MeOH/DCM = 1/25, V/V) to give target compounds **IIIa–IIId**.

##### 2–(2-(2-((1–(3-((5-(Dimethylamino)naphthalene)-1-sulfonamido)propyl)-1*H*-1,2,3-triazol-4-yl)methoxy)-4-methoxybenzoyl)-5-methoxyphenoxy)ethyl(2*S*,4a*S*,6a*S*,6b*R*,8a*R*,10*S*,12a*S*,12b*R*,14b*R*)-10-hydroxy-2,4a,6a,6b,9,9,12a-heptamethyl-13-oxo-1,2,3,4,4a,5,6,6a,6b,7,8,8a,9,10,11,12,12a,12b,13,14b-icosahydropicene-2-carboxylate (IIIa)

A light green solid was obtained in 65% yield. HPLC analysis: 95%, m.p. 144–147 °C. ^1^H-NMR (600 MHz, CDCl_3_) *δ* 8.54 (d, *J* = 8.5 Hz, 1H), 8.30 (d, *J* = 8.6 Hz, 1H), 8.21 (dd, *J* = 7.3, 1.3 Hz, 1H), 7.58 − 7.49 (m, 4H), 7.18 (d, *J* = 7.5 Hz, 1H), 6.75 (s, 1H), 6.55 (dd, *J* = 8.5, 2.2 Hz, 1H), 6.51 (d, *J* = 2.3 Hz, 1H), 6.49 (dd, *J* = 8.5, 2.3 Hz, 1H), 6.31 (d, *J* = 2.3 Hz, 1H), 5.65 (t, *J* = 6.3 Hz, 1H), 5.60 (s, 1H), 4.99 (s, 2H), 4.26 (t, *J* = 6.8 Hz, 2H), 4.04 − 3.94 (m, 4H), 3.87 (s, 3H), 3.80 (s, 3H), 3.22 (dd, *J* = 11.3, 4.9 Hz, 1H), 2.88 (s, 6H), 2.83 (q, *J* = 6.4 Hz, 2H), 2.76 (dt, *J* = 13.4, 3.6 Hz, 1H), 2.32 (s, 1H), 2.04 − 1.94 (m, 4H), 1.90 − 1.76 (m, 4H), 1.68 − 1.57 (m, 4H), 1.47 − 1.37 (m, 3H), 1.34 (s, 3H), 1.33 − 1.28 (m, 1H), 1.21 − 1.14 (m, 2H), 1.12 (s, 3H), 1.09 (s, 3H), 1.03 (s, 3H), 1.00 (s, 3H), 0.99 − 0.93 (m, 2H), 0.80 (s, 3H), 0.73 (s, 3H), 0.69 (dd, *J* = 11.9, 1.7 Hz, 1H). ^13^C-NMR (151 MHz, CDCl_3_) *δ* 200.30, 192.41, 176.11, 169.41, 163.75, 163.21, 159.20, 158.52, 152.14, 143.85, 134.49, 132.46, 132.22, 130.63, 129.95, 129.64, 129.54, 128.59, 128.45, 124.72, 124.15, 123.22, 122.48, 118.56, 115.31, 106.06, 105.65, 99.21, 99.16, 78.74, 66.68, 63.12, 62.26, 61.82, 55.64, 54.92, 48.20, 46.89, 45.41, 45.39, 43.91, 43.21, 40.96, 39.92, 39.14, 39.12, 37.69, 37.09, 32.74, 31.77, 30.96, 30.18, 28.45, 28.19, 28.10, 27.30, 26.44, 26.35, 23.38, 18.67, 17.48, 16.38, 15.59. Chemical Formula: C_65_H_83_N_5_O_11_S; HRMS (ESI-TOF) calculated for C_65_H_83_N_5_O_11_SNa (M + Na)^+^: 1164.5708; observed:1164.5712 (Figure 97–99 for supplementary file 1).

##### 2–(2–(2-(2-((1–(3-((5-(Dimethylamino)naphthalene)-1-sulfonamido)propyl)-1*H*-1,2,3-triazol-4-yl)methoxy)-4-methoxybenzoyl)-5-methoxyphenoxy)ethoxy)ethyl(2*S*,4a*S*,6a*S*,6b*R*,8a*R*,10*S*,12a*S*,12b*R*,14b*R*)-10-hydroxy-2,4a,6a,6b,9,9,12a-heptamethyl-13-oxo-1,2,3,4,4a,5,6,6a,6b,7,8,8a,9,10,11,12,12a,12b,13,14-bicosahydropicene-2-carboxylate (IIIb)

A light green solid was obtained in a 68% yield. HPLC analysis: 98%, m.p. 126–130 °C. ^1^H-NMR (600 MHz, CDCl_3_) *δ* 8.53 (d, *J* = 8.4 Hz, 1H), 8.30 (d, *J* = 8.6 Hz, 1H), 8.20 (d, *J* = 7.3 Hz, 1H), 7.54 (ddd, *J* = 17.3, 13.3, 8.5 Hz, 4H), 7.17 (d, *J* = 7.5 Hz, 1H), 6.71 (s, 1H), 6.56 (dd, *J* = 8.6, 2.0 Hz, 1H), 6.52 (d, *J* = 1.9 Hz, 1H), 6.46 (dd, *J* = 8.5, 2.1 Hz, 1H), 6.35 − 6.31 (m, 1H), 5.84 (dt, *J* = 12.4, 5.1 Hz, 1H), 5.62 (s, 1H), 5.00 (s, 2H), 4.23 (t, *J* = 6.5 Hz, 2H), 4.18 (ddd, *J* = 11.7, 5.6, 3.6 Hz, 1H), 4.05 − 3.99 (m, 1H), 3.94 (d, *J* = 4.2 Hz, 2H), 3.85 (s, 3H), 3.79 (s, 3H), 3.45 − 3.34 (m, 4H), 3.21 (dd, *J* = 11.3, 4.6 Hz, 1H), 2.87 (s, 6H), 2.83 (q, *J* = 6.3 Hz, 2H), 2.73 (d, *J* = 13.0 Hz, 1H), 2.32 (s, 1H), 2.16 − 2.04 (m, 2H), 2.03 − 1.93 (m, 4H), 1.88 (d, *J* = 13.5 Hz, 1H), 1.79 (td, *J* = 13.6, 4.5 Hz, 1H), 1.68 − 1.58 (m, 5H), 1.45 − 1.37 (m, 2H), 1.35 (s, 3H), 1.33 − 1.23 (m, 3H), 1.16 (d, *J* = 16.3 Hz, 1H), 1.12 (s, 3H), 1.10 (s, 3H), 1.08 (s, 3H), 1.00 (s, 3H), 0.98 − 0.92 (m, 1H), 0.80 (s, 3H), 0.75 (s, 3H), 0.68 (d, *J* = 11.2 Hz, 1H). ^13^C-NMR (151 MHz, CDCl_3_) *δ* 200.28, 192.59, 176.33, 169.55, 163.54, 163.38, 159.60, 158.63, 152.07, 143.83, 134.57, 132.36, 132.20, 130.57, 129.91, 129.54, 128.53, 128.28, 124.57, 124.30, 123.21, 122.49, 118.64, 115.29, 105.76, 105.34, 99.18, 98.51, 78.67, 69.43, 69.18, 68.47, 63.20, 63.05, 61.81, 55.62, 55.58, 54.90, 48.33, 46.92, 45.40, 45.37, 44.01, 43.18, 41.10, 39.90, 39.13, 39.11, 37.60, 37.04, 32.72, 31.77, 31.14, 30.16, 28.53, 28.21, 28.11, 27.25, 26.48, 26.37, 23.35, 18.65, 17.47, 16.36, 15.63. Chemical Formula: C_67_H_87_N_5_O_12_S; HRMS (ESI-TOF) calculated for C_67_H_87_N_5_O_12_SNa (M + Na)^+^: 1208.5970; observed: 1208.5996 (Figure 100–102 for supplementary file 1).

##### 2–(2–(2-(2-(2-((1–(3-((5-(Dimethylamino)naphthalene)-1-sulfonamido)propyl)-1*H*-1,2,3-triazol-4-yl)methoxy)-4-methoxybenzoyl)-5-methoxyphenoxy)ethoxy)ethoxy)ethyl(2*S*,4a*S*,6a*S*,6b*R*,8a*R*,10*S*,12a*S*,12b*R*,14b*R*)-10-hydroxy-2,4a,6a,6b,9,9,12a-heptamethyl-13-oxo-1,2,3,4,4a,5,6,6a,6b,7,8,8a,9,10,11,12,12a,12b,13,14b-icosahydropicene-2-carboxylate (IIIc)

A light green solid was obtained in 78% yield. HPLC analysis: 98%, m.p. 110–114 °C. ^1^H-NMR (600 MHz, CDCl_3_) *δ* 8.54 (d, *J* = 8.5 Hz, 1H), 8.29 (d, *J* = 8.6 Hz, 1H), 8.21 (dd, *J* = 7.3, 1.4 Hz, 1H), 7.57 − 7.49 (m, 4H), 7.18 (d, *J* = 7.6 Hz, 1H), 6.73 (s, 1H), 6.55 (dd, *J* = 8.6, 2.2 Hz, 1H), 6.51 (d, *J* = 2.2 Hz, 1H), 6.48 (dd, *J* = 8.5, 2.3 Hz, 1H), 6.28 (d, *J* = 2.4 Hz, 1H), 5.64 (d, *J* = 3.6 Hz, 2H), 5.01 (s, 2H), 4.28 − 4.22 (m, 3H), 4.16 (dt, *J* = 11.9, 4.7 Hz, 1H), 3.91 − 3.87 (m, 2H), 3.85 (s, 3H), 3.79 (s, 3H), 3.68 − 3.58 (m, 2H), 3.51 (dd, *J* = 5.6, 3.3 Hz, 2H), 3.39 (dt, *J* = 9.7, 5.0 Hz, 4H), 3.22 (dd, *J* = 11.3, 4.9 Hz, 1H), 2.88 (s, 6H), 2.82 (q, *J* = 6.3 Hz, 2H), 2.76 (dt, *J* = 13.3, 3.5 Hz, 1H), 2.33 (s, 1H), 2.11 (dd, *J* = 14.5, 3.4 Hz, 1H), 2.03 − 1.88 (m, 6H), 1.81 (td, *J* = 13.7, 4.7 Hz, 1H), 1.68 − 1.56 (m, 5H), 1.47 − 1.38 (m, 3H), 1.36 (s, 3H), 1.31 (d, *J* = 27.7 Hz, 2H), 1.20 − 1.15 (m, 1H), 1.13 (s, 3H), 1.12 (s, 3H), 1.11 (s, 3H), 1.00 (s, 3H), 0.99 − 0.93 (m, 1H), 0.80 (s, 3H), 0.78 (s, 3H), 0.69 (d, *J* = 11.8 Hz, 1H). ^13^C-NMR (151 MHz, CDCl_3_) *δ* 200.23, 192.61, 176.35, 169.42, 163.53, 163.32, 159.58, 158.58, 152.10, 134.52, 132.33, 132.31, 130.60, 129.92, 129.61, 129.53, 128.57, 128.46, 124.54, 124.46, 123.23, 122.46, 118.61, 115.31, 105.51, 105.43, 99.21, 98.77, 78.73, 70.70, 70.35, 69.31, 69.13, 68.31, 63.23, 63.12, 61.82, 55.59, 54.92, 48.24, 46.90, 45.42, 45.39, 44.01, 43.20, 41.09, 39.90, 39.14, 37.68, 37.08, 32.75, 31.80, 31.14, 30.18, 28.55, 28.28, 28.11, 27.28, 26.48, 26.40, 23.41, 18.69, 17.49, 16.38, 15.61. Chemical Formula: C_69_H_91_N_5_O_13_S; HRMS (ESI-TOF) calculated for C_69_H_91_N_5_O_13_SNa (M + Na)^+^: 1252.6232; observed: 1252.6228 (Figure 103–105 for supplementary file 1).

##### 2–(2–(2–(2-(2-(2-((1–(3-((5-(Dimethylamino)naphthalene)-1-sulfonamido)propyl)-1*H*-1,2,3-triazol-4-yl)methoxy)-4-methoxybenzoyl)-5-methoxyphenoxy)ethoxy)ethoxy)ethoxy)ethyl(2*S*,4a*S*,6a*S*,6b*R*,8a*R*,10*S*,12a*S*,12b*R*,14b*R*)-10-hydroxy-2,4a,6a,6b,9,9,12a-heptamethyl-13-oxo-1,2,3,4,4a,5,6,6a,6b,7,8,8a,9,10,11,12,12a,12b,13,14b-icosahydropicene-2-carboxylate (IIId)

A light green solid was obtained in 63% yield. HPLC analysis: 99%, m.p. 106–110 °C. ^1^H-NMR (600 MHz, CDCl_3_) *δ* 8.54 (d, *J* = 8.5 Hz, 1H), 8.29 (d, *J* = 8.6 Hz, 1H), 8.21 (d, *J* = 8.5 Hz, 1H), 7.58 − 7.49 (m, 4H), 7.18 (d, *J* = 7.5 Hz, 1H), 6.72 (s, 1H), 6.55 (dd, *J* = 8.6, 2.2 Hz, 1H), 6.51 (d, *J* = 2.2 Hz, 1H), 6.48 (dd, *J* = 8.6, 2.3 Hz, 1H), 6.27 (d, *J* = 2.3 Hz, 1H), 5.64 (s, 1H), 5.62 (t, *J* = 5.4 Hz, 1H), 5.02 (s, 2H), 4.30 − 4.22 (m, 3H), 4.18 (dt, *J* = 12.0, 4.4 Hz, 1H), 3.89 (t, *J* = 4.8 Hz, 2H), 3.85 (s, 3H), 3.79 (s, 3H), 3.66 (dd, *J* = 6.8, 2.8 Hz, 2H), 3.59 (d, *J* = 6.9 Hz, 4H), 3.52 − 3.48 (m, 2H), 3.38 (dt, *J* = 14.0, 4.5 Hz, 4H), 3.22 (dd, *J* = 11.4, 4.9 Hz, 1H), 2.88 (s, 6H), 2.82 (q, *J* = 6.3 Hz, 2H), 2.77 (dt, *J* = 13.5, 3.6 Hz, 1H), 2.33 (s, 1H), 2.14 − 2.10 (m, 1H), 2.04 − 1.90 (m, 6H), 1.81 (td, *J* = 13.7, 4.7 Hz, 1H), 1.67 − 1.58 (m, 5H), 1.43 (dd, *J* = 28.1, 12.7 Hz, 3H), 1.36 (s, 3H), 1.34 − 1.29 (m, 2H), 1.17 (d, *J* = 14.1 Hz, 1H), 1.14 (s, 3H), 1.12 (s, 3H), 1.11 (s, 3H), 1.00 (s, 3H), 0.95 (dd, *J* = 13.3, 4.0 Hz, 1H), 0.80 (s, 3H), 0.79 (s, 3H), 0.69 (d, *J* = 13.8 Hz, 1H). ^13^C-NMR (151 MHz, CDCl_3_) *δ* 200.20, 192.62, 176.37, 169.37, 163.54, 163.31, 159.57, 158.58, 152.09, 143.99, 134.53, 132.31, 130.60, 129.92, 129.61, 129.53, 128.56, 128.48, 124.53, 124.46, 123.23, 122.42, 118.62, 115.32, 105.52, 105.47, 99.19, 98.73, 78.73, 70.68, 70.49, 70.41, 69.27, 69.17, 68.32, 63.28, 63.12, 61.82, 55.59, 54.93, 48.24, 46.88, 45.42, 45.38, 44.02, 43.19, 41.10, 39.89, 39.14, 37.69, 37.08, 32.75, 31.81, 31.15, 30.19, 28.55, 28.29, 28.11, 27.29, 26.48, 26.41, 23.41, 18.68, 17.49, 16.38, 15.61. Chemical Formula: C_71_H_95_N_5_O_14_S; HRMS (ESI-TOF) calculated for C_71_H_95_N_5_O_14_SNa (M + Na)^+^: 1296.6494; observed: 1296.6490 (Figure 106–108 for supplementary file 1).

### Cell culture

RAW 264.7 cells were purchased from the National Collection of Authenticated Cell Cultures (catalog no. SCSP-5036). The cells were cultured in DMEM medium (11965–118, Gibco), supplemented with 10% FBS (10099141, Gibco), 1% penicillin and streptomycin (PB180120, Procell, Wuhan, China), and were maintained at 37 °C in a humidified atmosphere containing 5% CO_2_.

#### ELISA for detecting inflammatory factors

Two mL of a RAW 264.7 cell suspension with a density of 2 × 10^4^ ml and 500 µL in 6-well plates was prepared and cultured at 37 °C in a 5% CO_2_ incubator. After 24 h, treatment was carried out according to the required experimental groupings (18*β*-GA 10 µg/mL or a series of compounds at 10 µg/mL). After 24 h, the cells were stimulated with LPS (1 µg/mL) (HY-D1056, MCE, Shanghai, China) for 12 h, and the supernatant was collected to detect the levels of TNF-α, IL-1β, and IL-6 using ELISA. Mouse TNF-α, IL-1β and IL-6 ELISA Kits were used: PT512, PI301 and PI326 (Beyotime, China).

#### Western blotting for detecting inflammatory pathway-related proteins

Cell samples were collected, and the freeze-dried samples were dissolved in 500 µL RIPA lysis buffer (P0013C, Beyotime, China). After centrifugation at 14000 g for 30 min at 4 °C, the proteins were collected and quantified. Determination of protein concentration was performed using BCA assays. After denaturation at 95 °C and cooling, the protein was separated by SDS-PAGE electrophoresis. A 10% SDS-PAGE separation gel was added, and a 4% concentrated gel was added for gel filling. The voltage was set to 40 V, and the film transfer was performed for 4 h. After decolorisation at room temperature, 5% skim milk powder was used, and the mixture was incubated for 2 h. The primary antibody was incubated overnight at 4 °C, and the secondary antibody (7074, CST, 1:6000) was incubated at r.t. for 1 h. After washing the membranes with TBST, chemiluminescence was detected. A T4600 was used for protein blotting. GAPDH (8884, CST, 1:5000), HDAC8 (#66042, CST, 1:1500), P-STAT3 (#9154, CST, 1:1500), STAT3 (#12640, CST, 1:1000), SOCS3 (#52113, CST, 1:2000), SDS-PAGE reagents (M00657, Genescript, Nanjing, China).

### Immunofluorescence

Two mL of RAW 264.7 cell suspension with a density of 2 × 10^4^ ml and 500 µL in 6-well plates were prepared and cultured at 37 °C in a 5% CO_2_ incubator. After 24 h, treatment was carried out according to the required experimental groupings (Compounds **Ia** and **IIc** at 10 µg/mL). After 24 h, the cells were fixed with 4% paraformaldehyde and washed with PBS (pH 7.4). After slightly drying the slices, FITC phalloidin (40735ES75, Yeasen, Shanghai, China) was added dropwise to the circles for staining and incubated at r.t. in the dark for 10 min. After washing again with PBS, the slices were sealed with an anti-fluorescence quenching sealing agent (G1401, ServiceBio), observed under a fluorescence microscope, and images were collected. Compounds **Ia** and **IIc** emitted blue light at a UV excitation wavelength of 330–380 nm, an emission wavelength of 420 nm, FITC excitation wavelength of 495 nm, emission wavelength of 520 nm, and green emission.

After 24 h, the cells were fixed with 4% paraformaldehyde and washed with PBS (pH 7.4) (Cell processing was the same as above). Then, incubate with 1 ml of Phalloidin-TRITC (HY-P2270, MCE) working solution at room temperature for 5–10 min. After washing with PBS, dry the slides slightly and SYTM Green (HY-D2208, MCE) was added dropwise to the circles for staining and incubated at r.t. in the dark for 10 min. After washing again with PBS, the slices were sealed with an anti-fluorescence quenching sealing agent (G1401, ServiceBio), observed under a fluorescence microscope, and images were collected. Compounds **Ia** and **IIc** emitted blue light at a UV excitation wavelength of 330–380 nm, an emission wavelength of 420 nm, Phalloidin-TRITC excitation wavelength of 540–546 nm, emission wavelength of 565–575 nm, and red emission, SYTM Green excitation wavelength of 503 nm, emission wavelength of 530 nm, and green emission.

### Statistical analysis

Statistical analyses and plotting were performed using GraphPad Prism version 8.0.2. Multiple differences were analysed using one-way ANOVA. All experiments were repeated at least three times, and the data are expressed as mean ± standard deviation (SD). *p* < 0.05 was considered statistically significant.

## Supplementary Material

supplementary file 1.docx

## Data Availability

The data presented in this study are available upon request from the corresponding author.
