# Supplementary material for: Application of 18β-glycyrrhetinic acid Fluorescent probes in cell imaging
Source: J Enzyme Inhib Med Chem. 2026 Mar 9;41(1):2631869. doi: 10.1080/14756366.2026.2631869 (PMC12973793; doi:10.1080/14756366.2026.2631869)
Supplement: supplementary file 1.docx [file IENZ_A_2631869_SM6717.docx]

Spectrum of Class Ⅰ compounds:


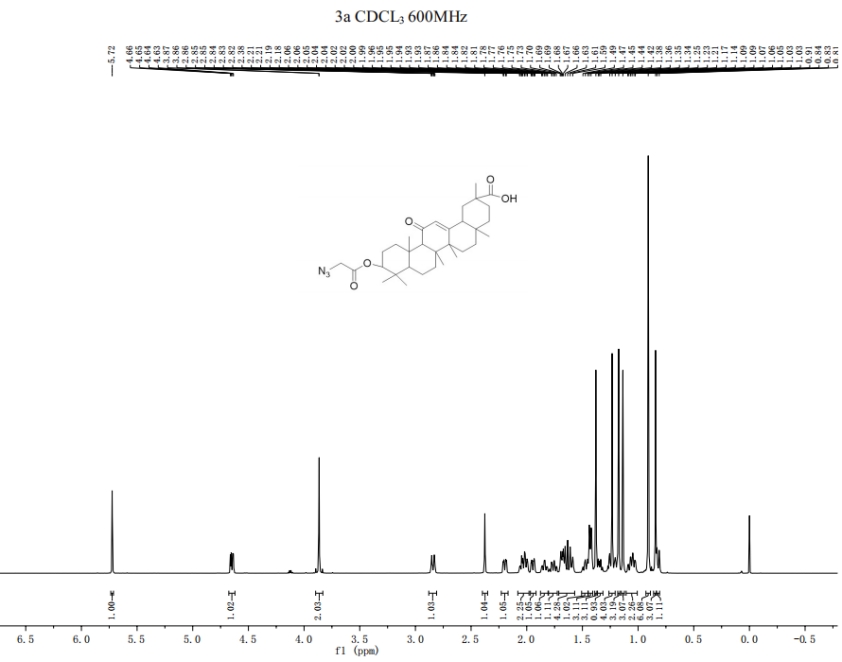


Fig. 1 The ^1^H-NMR spectrum of compound **9a**


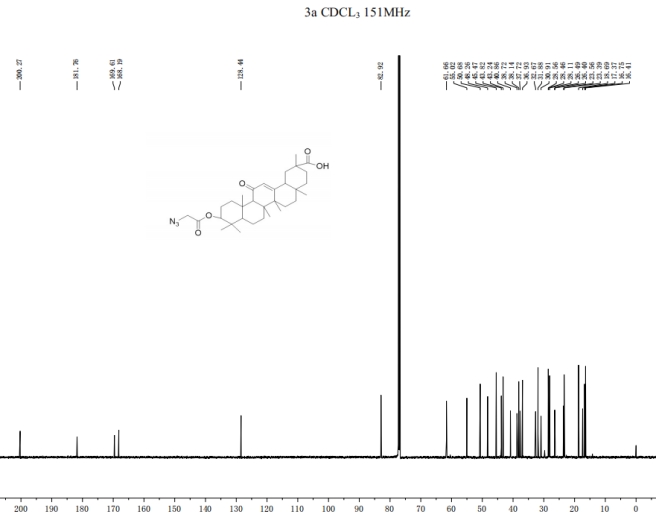


Fig. 2 The ^13^C-NMR spectrum of compound **9a**

|  |  |
| --- | --- |
|  | 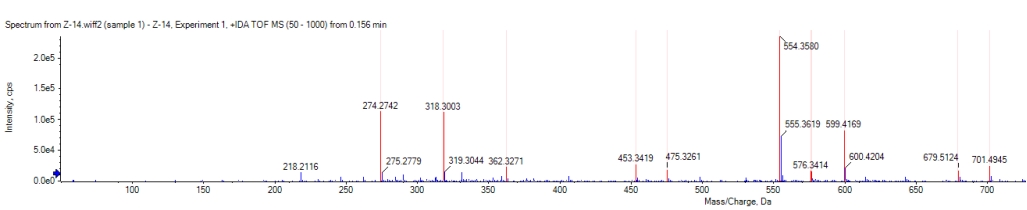 |

Fig. 3 The high-resolution mass spectrum of compound **9a**


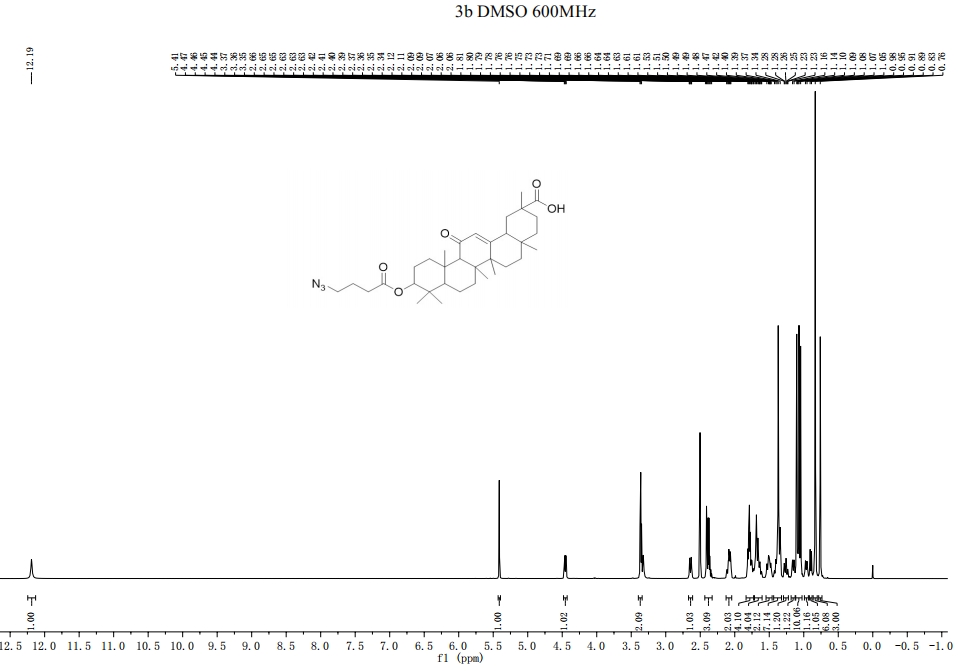


Fig. 4 The ^1^H-NMR spectrum of compound **9b**

|  |  |
| --- | --- |
|  | 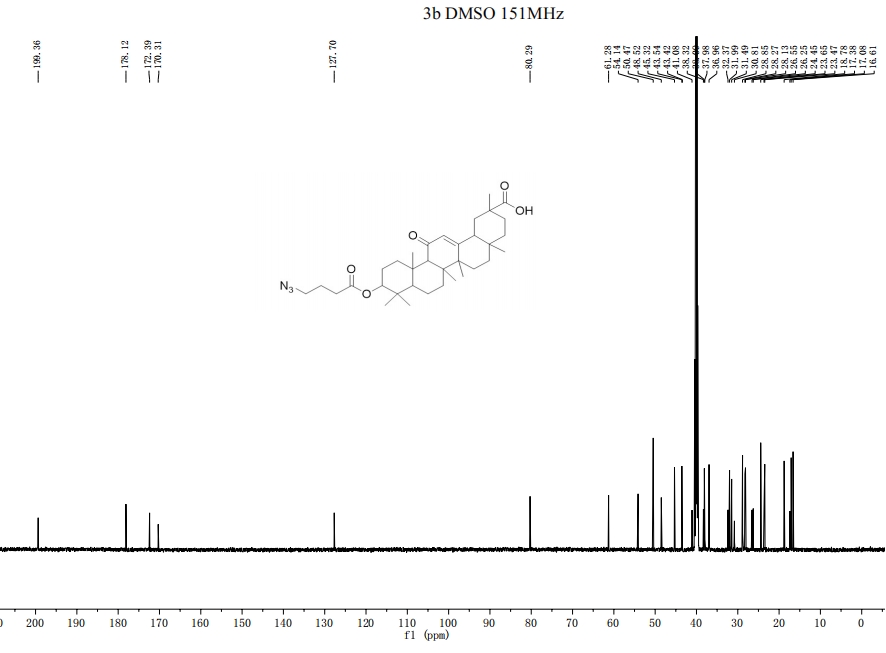 |

Fig. 5 The ^13^C-NMR spectrum of compound **9b**

|  |  |
| --- | --- |
|  | 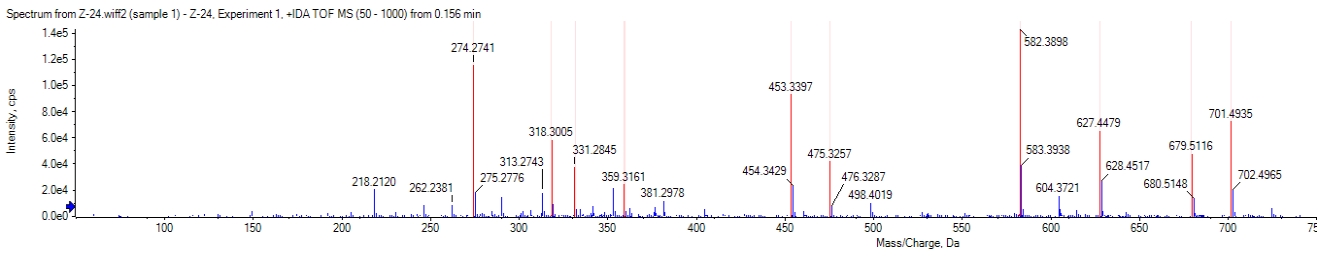 |

Fig. 6 The high-resolution mass spectrum of compound **9b**


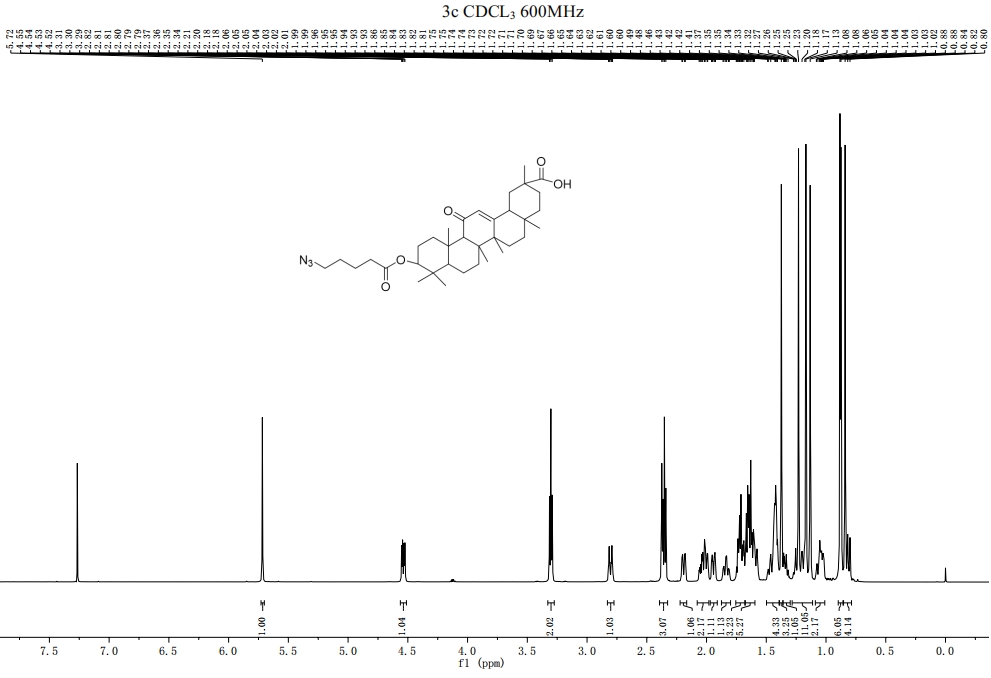


Fig. 7 The ^1^H-NMR spectrum of compound **9c**

|  |  |
| --- | --- |
|  | 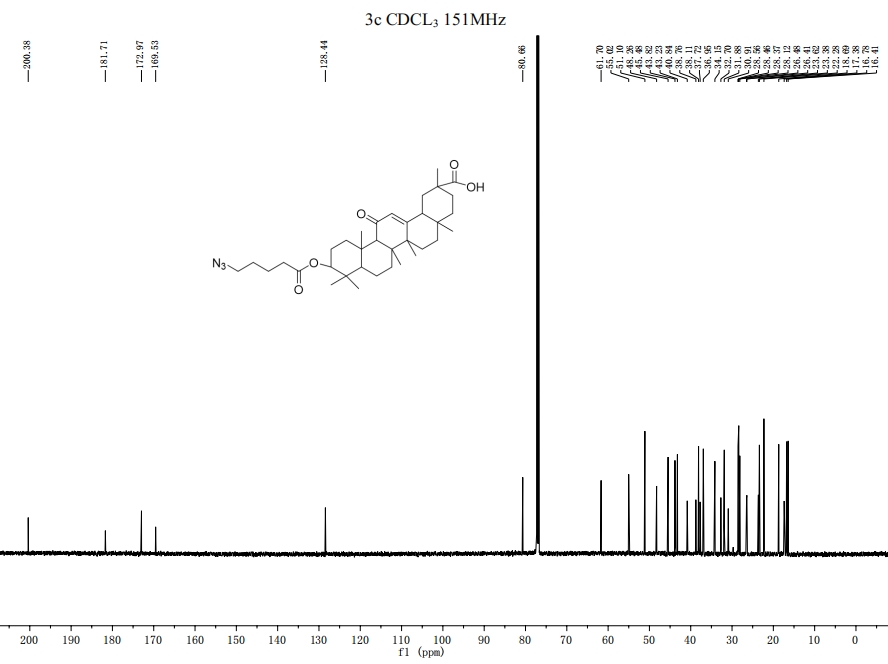 |

Fig. 8 The ^13^C-NMR spectrum of compound **9c**

|  |  |
| --- | --- |
|  | 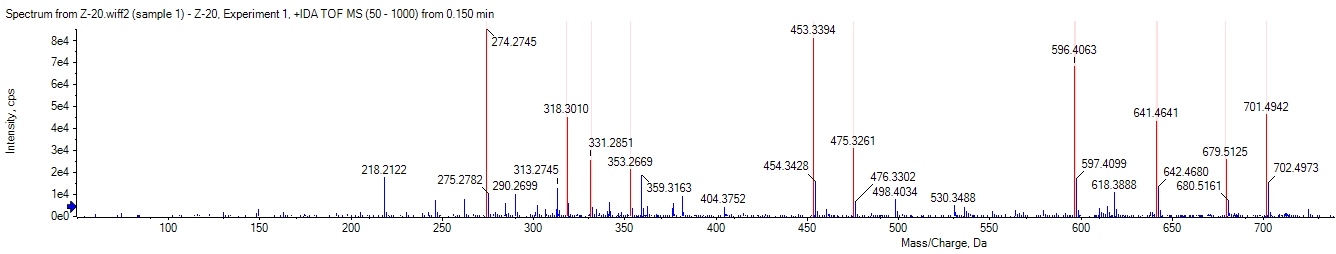 |

Fig. 9 The high-resolution mass spectrum of compound **9c**

|  |  |
| --- | --- |
|  | 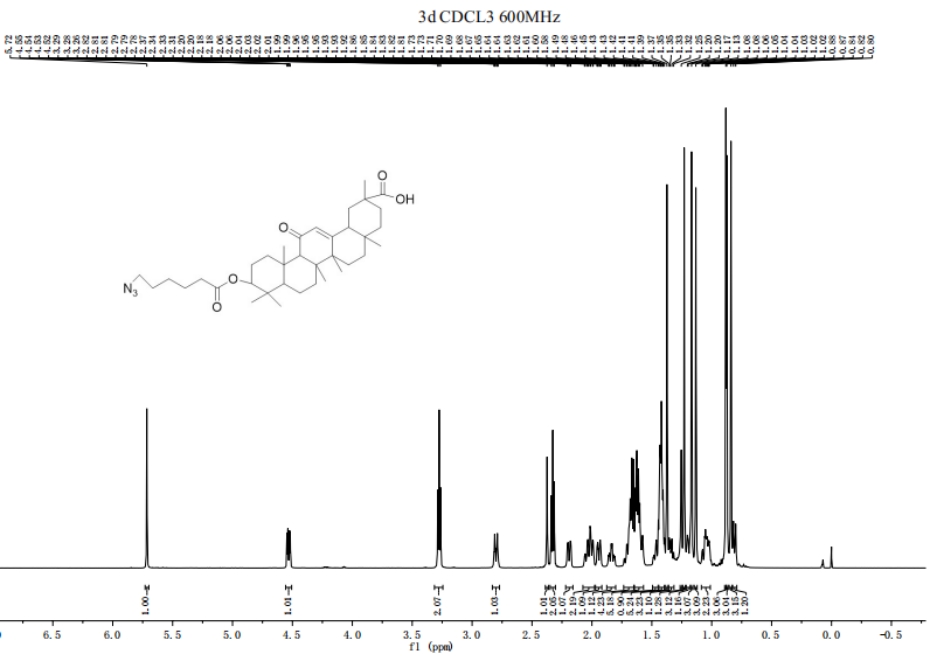 |

Fig. 10 The ^1^H-NMR spectrum of compound **9d**


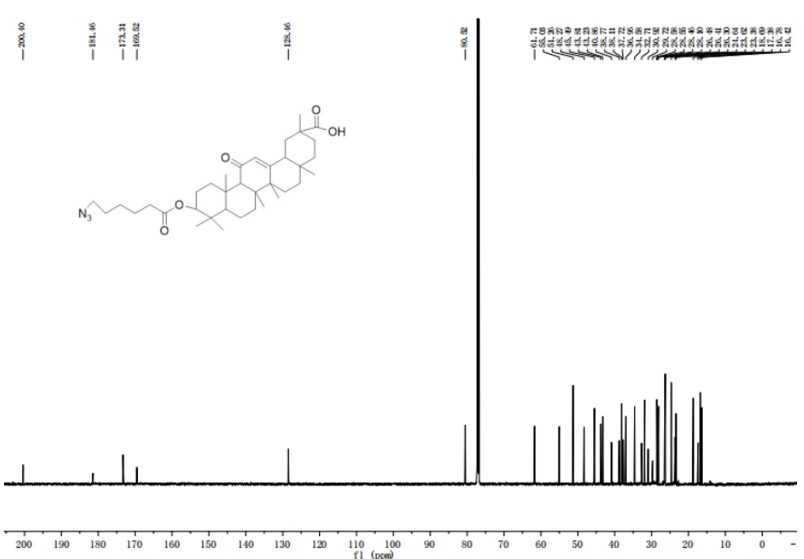


Fig. 11 The ^13^C-NMR spectrum of compound **9d**

|  |  |
| --- | --- |
|  | 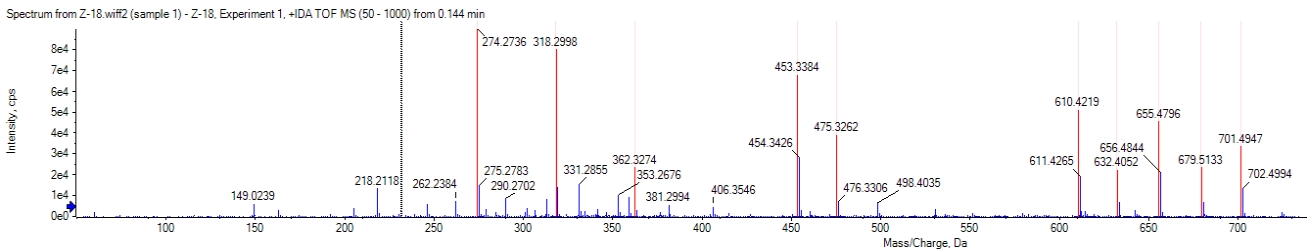 |

Fig. 12 The high-resolution mass spectrum of compound **9d**


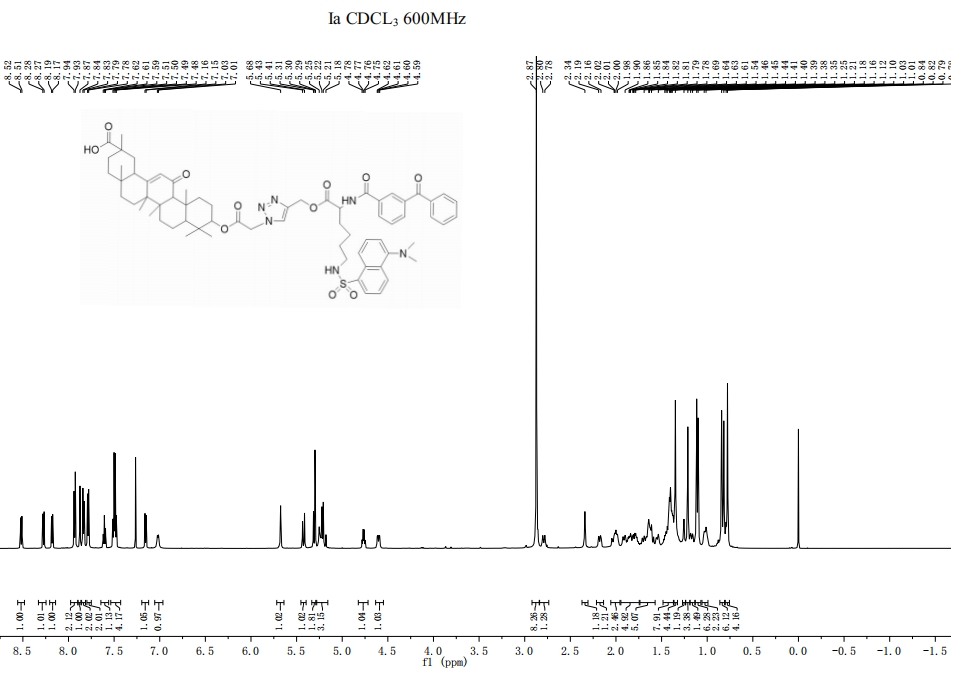


Fig. 13 The ^1^H-NMR spectrum of compound **Ⅰa**

| 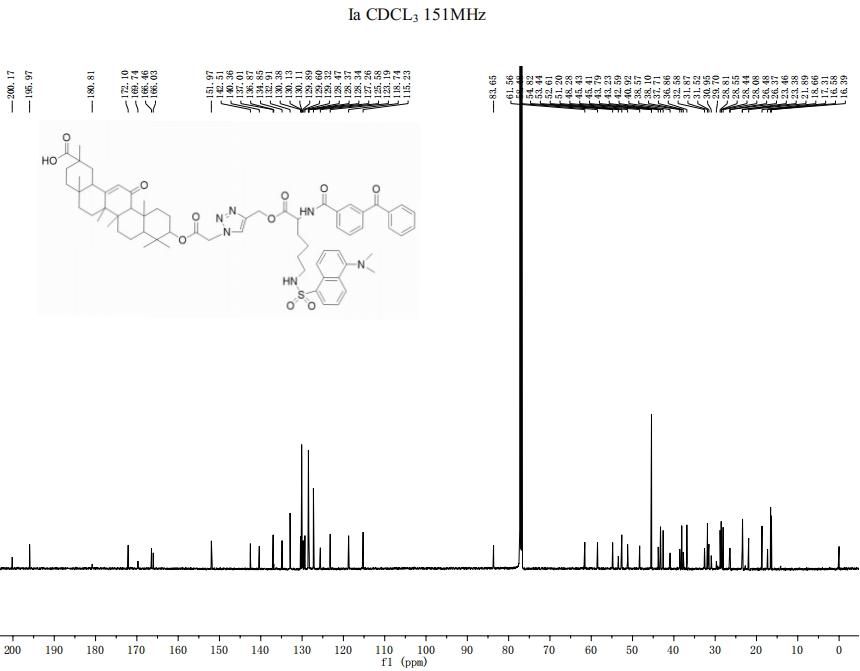 |
| --- |
|  |

|  |
| --- |

Fig. 14 The ^13^C-NMR spectrum of compound **Ⅰa**

|  |  |
| --- | --- |
|  | 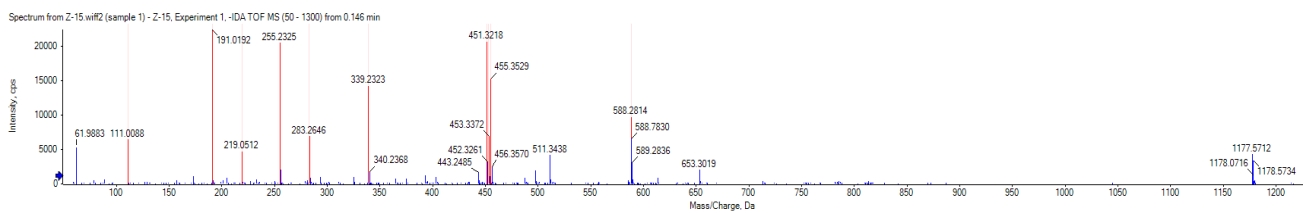 |

Fig. 15 The high-resolution mass spectrum of compound **Ⅰa**

|  |  |
| --- | --- |
|  | 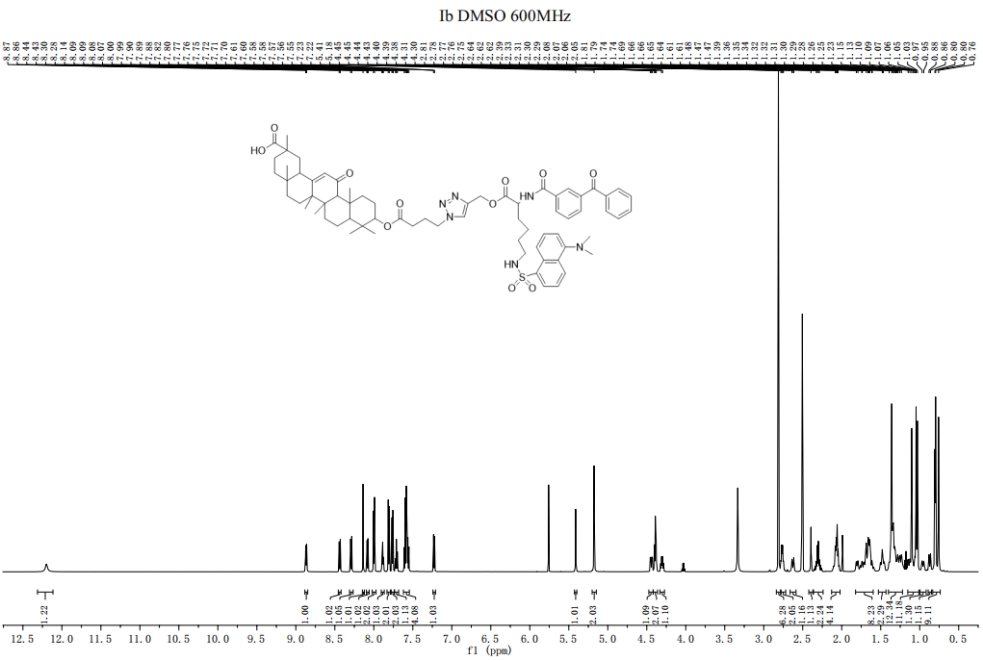 |

Fig. 16 The ^1^H-NMR spectrum of compound **Ⅰb**

|  |  |
| --- | --- |
|  | 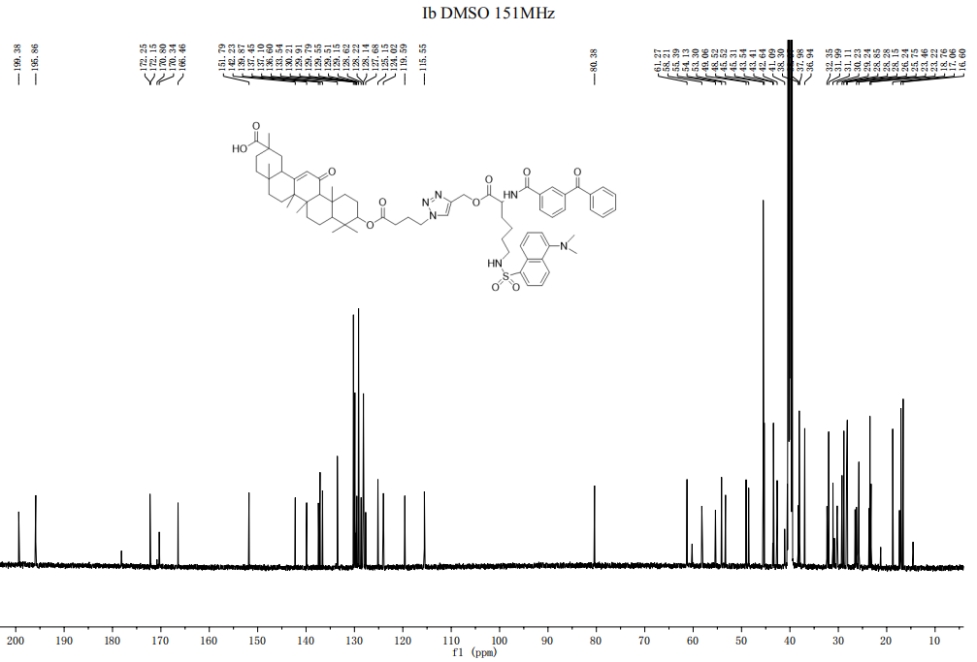 |

Fig. 17 The ^13^C-NMR spectrum of compound **Ⅰb**

|  |  |
| --- | --- |
|  | 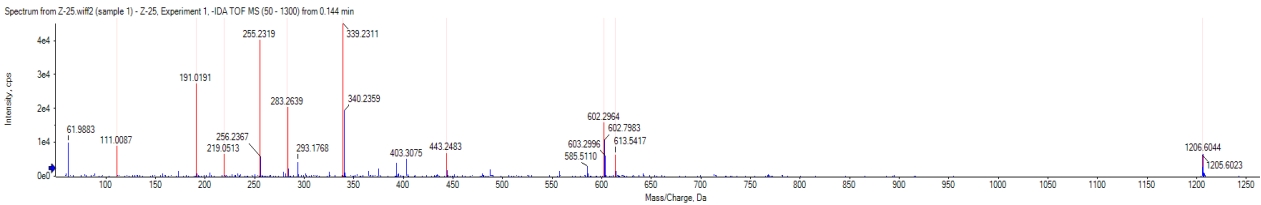 |

Fig. 18 The high-resolution mass spectrum of compound **Ⅰb**

|  |
| --- |
| 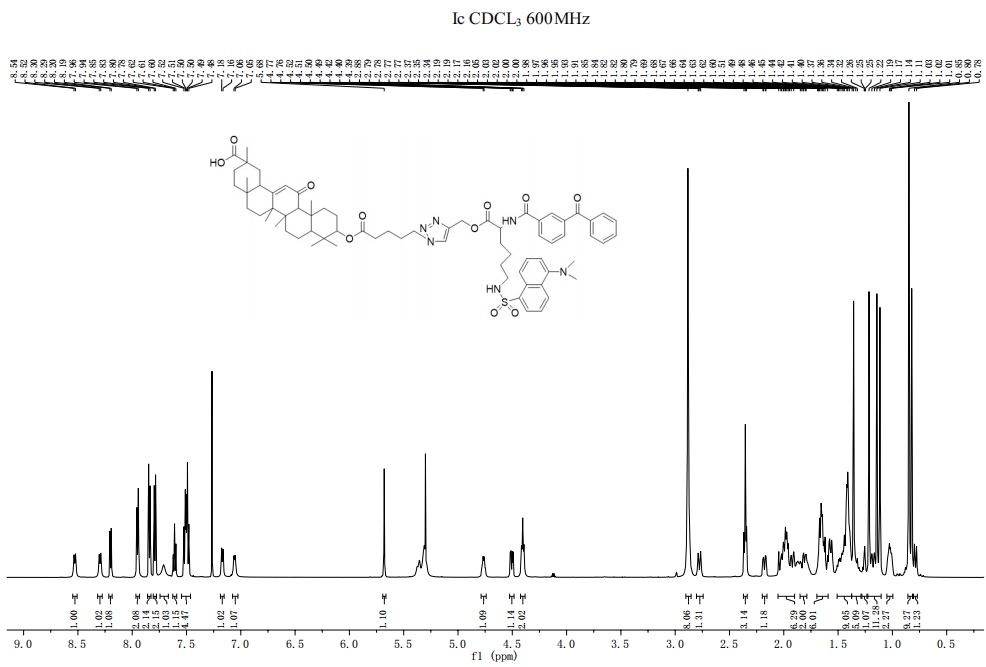  Fig. 19 The ^1^H-NMR spectrum of compound **Ⅰc** 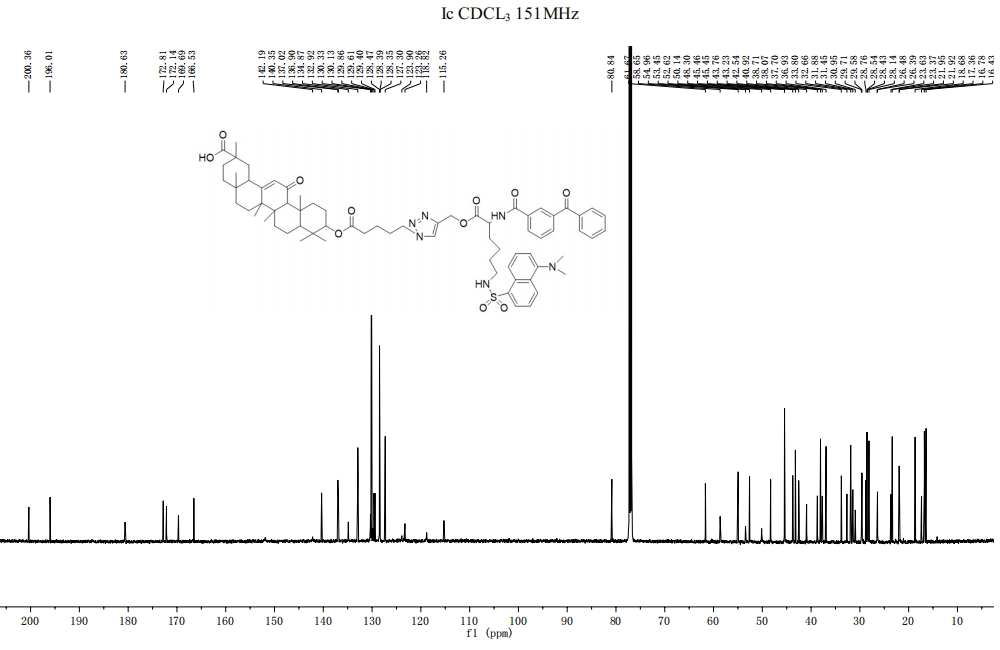 |

|  |  |
| --- | --- |
|  |  |

Fig. 20 The ^13^C-NMR spectrum of compound **Ⅰc**

|  |  |
| --- | --- |
|  | 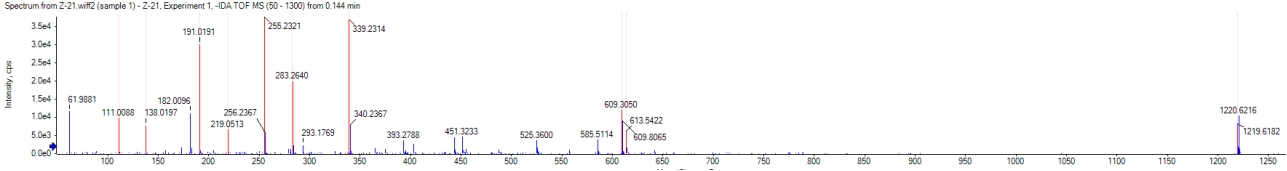 |

Fig. 21 The high-resolution mass spectrum of compound **Ⅰc**

|  |  |
| --- | --- |
|  | 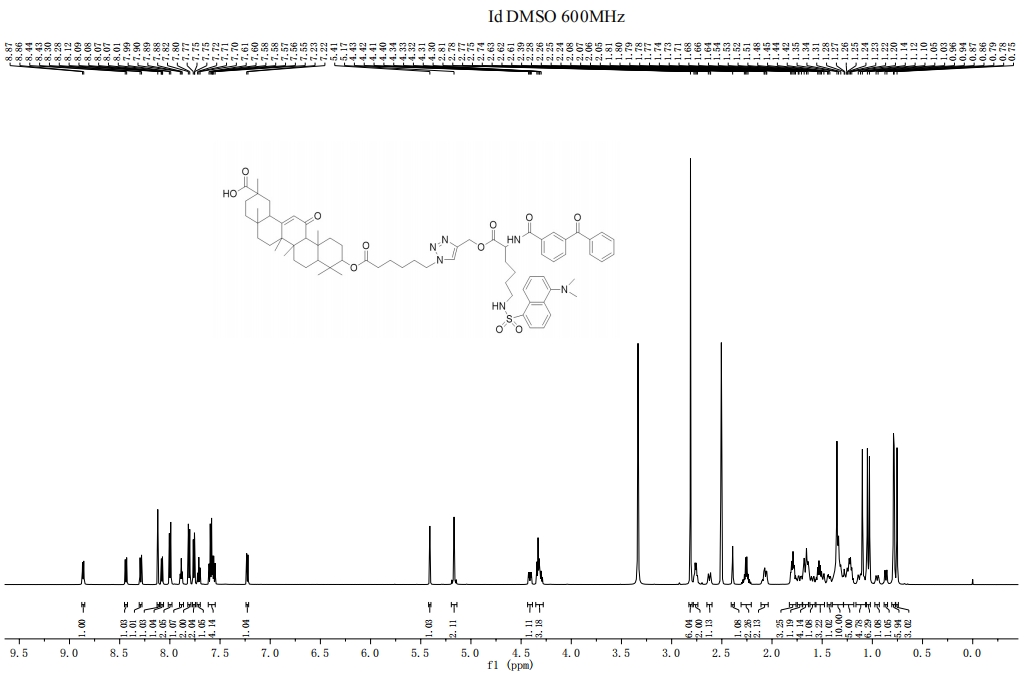 |

Fig. 22 The ^1^H-NMR spectrum of compound **Ⅰd**

|  | 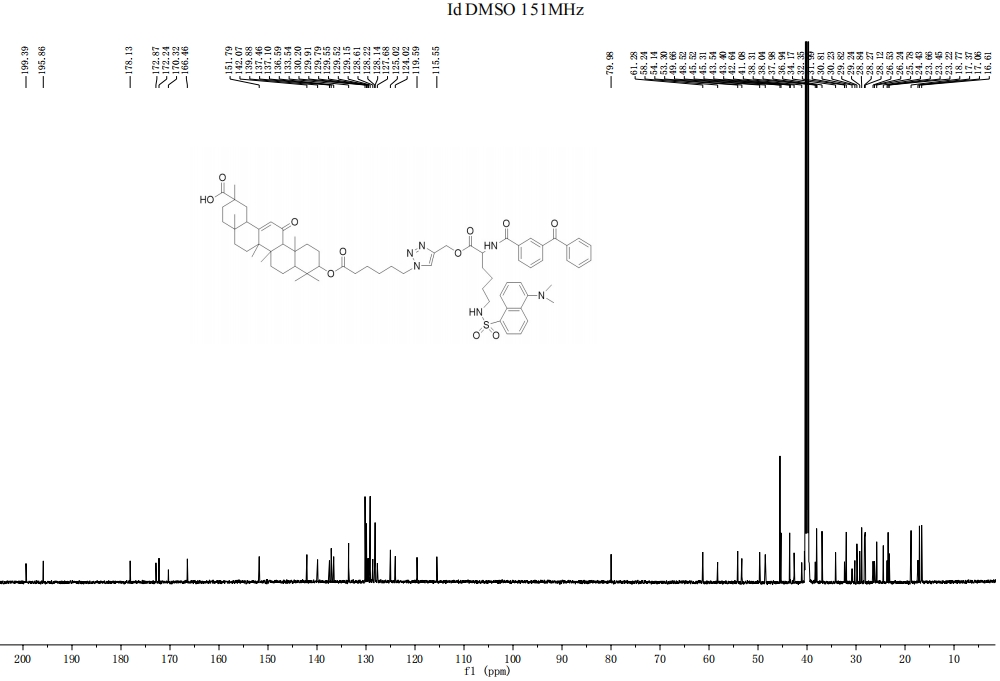 |
| --- | --- |

Fig. 23 The ^13^C-NMR spectrum of compound **Ⅰd**

|  |  |
| --- | --- |
|  | 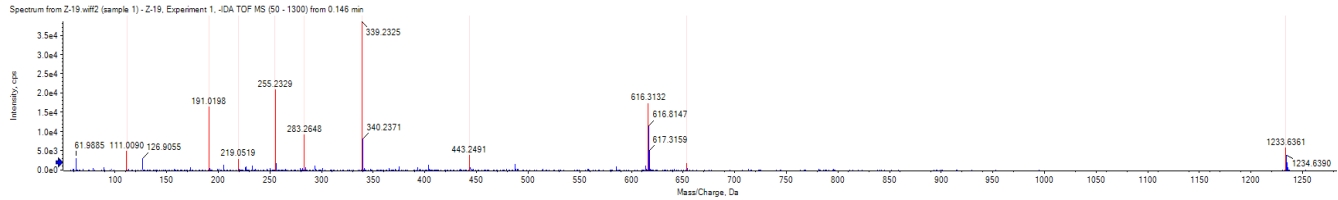 |

Fig. 24 The high-resolution mass spectrum of compound **Ⅰd**

Spectrum of Class II compounds:


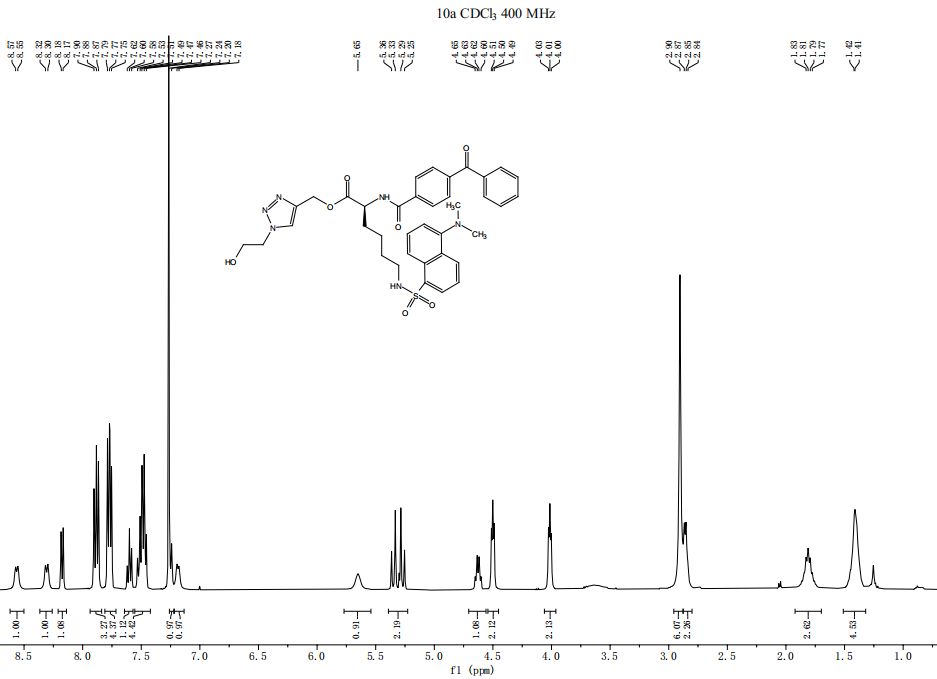


Fig. 25 ^1^H-NMR spectrum of compound **10a**


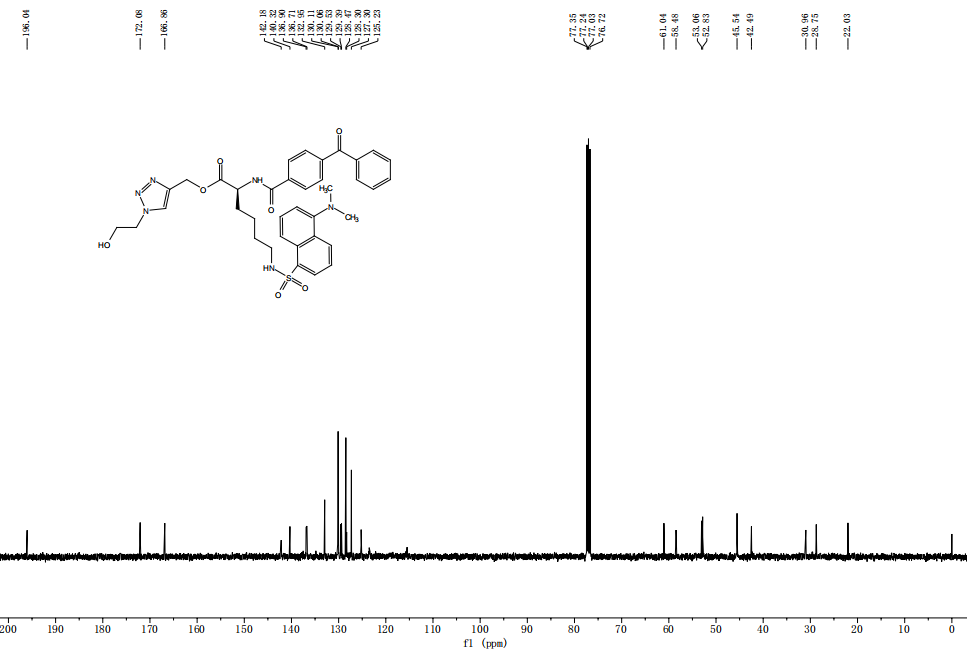


Fig. 26 ^13^C-NMR spectrum of compound **10a**


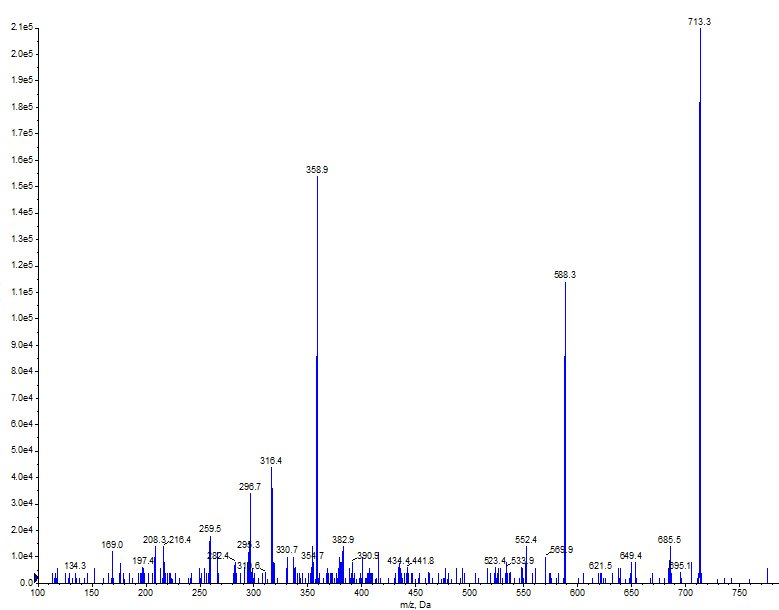


Fig. 27 Low resolution mass spectra of compound **10a**


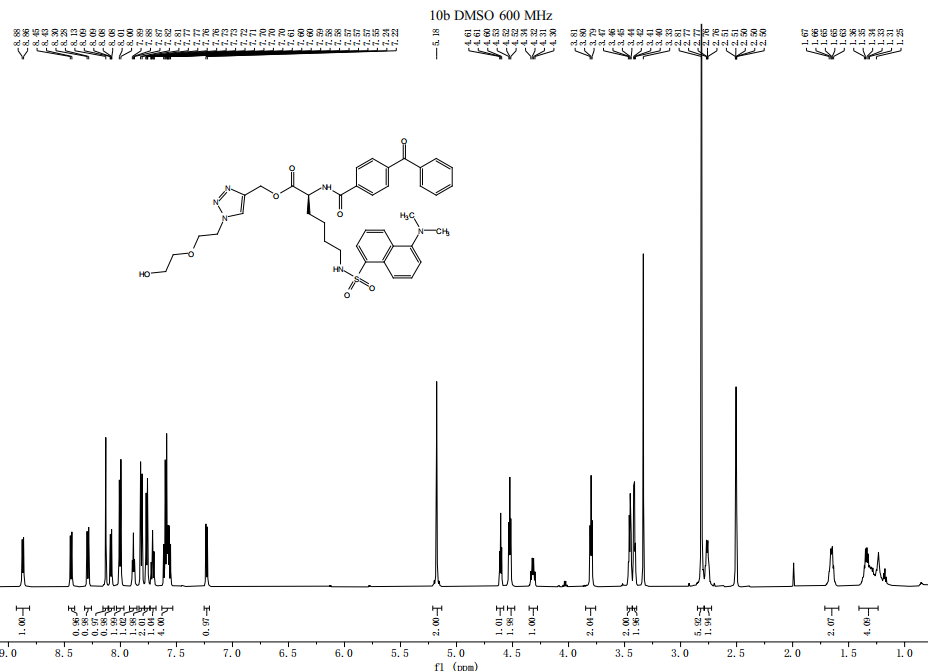


Fig. 28 ^1^H-NMR spectrum of compound **10b**


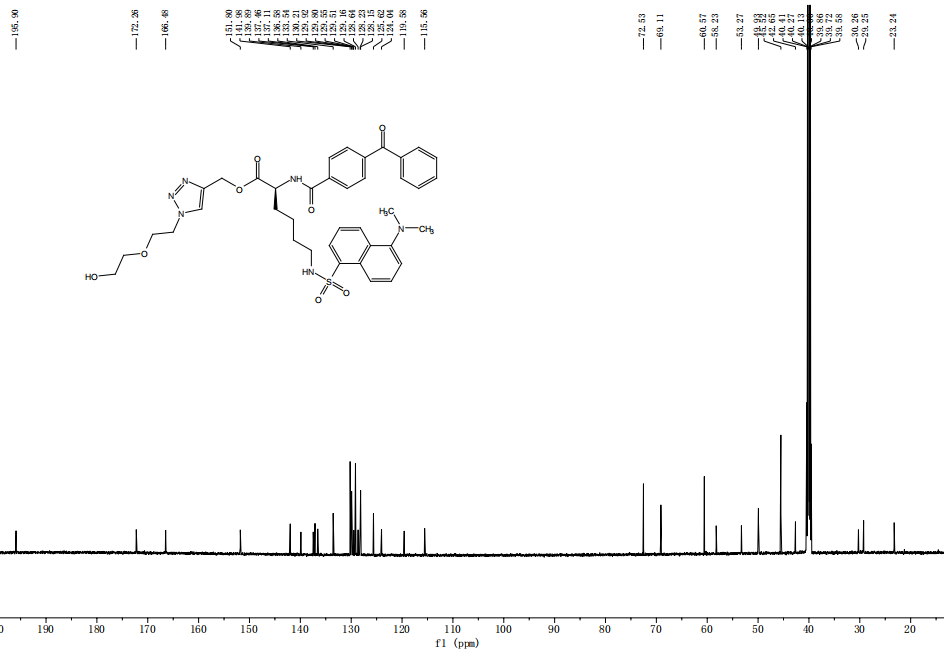


Fig. 29 ^13^C-NMR spectrum of compound **10b**

Fig. 30 High resolution mass spectra of compound **10b**


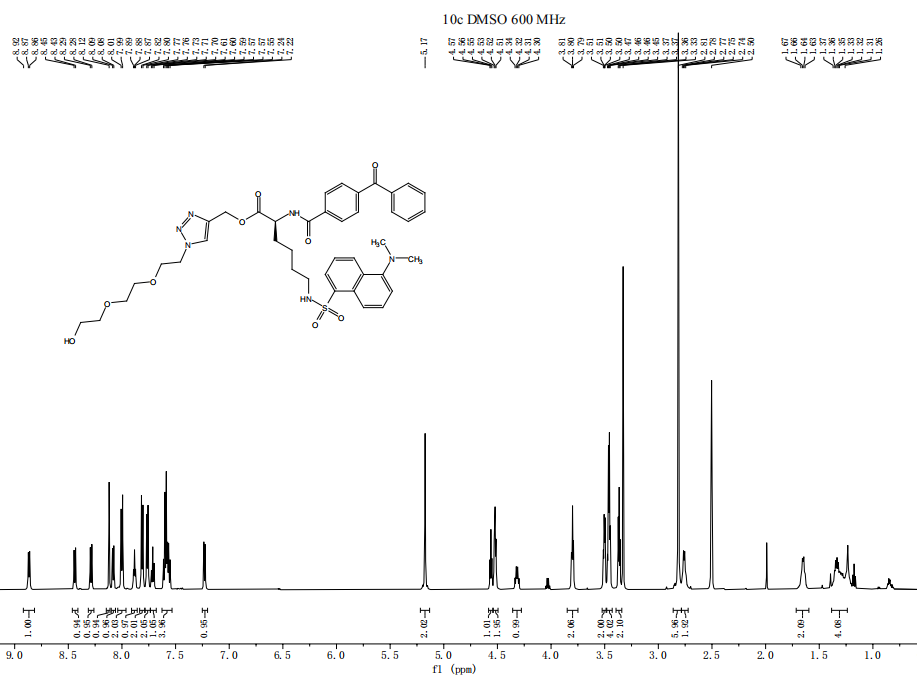


Fig. 31 ^1^H-NMR spectrum of compound **10c**


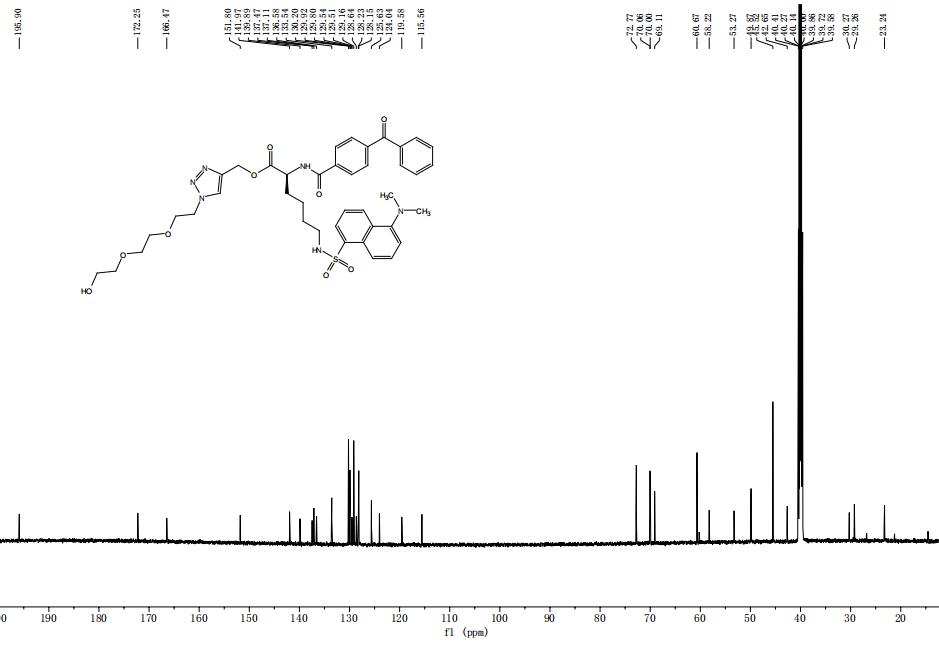


Fig. 32 ^13^C-NMR spectrum of compound **10c**

Fig. 33 High resolution mass spectra of compound **10c**


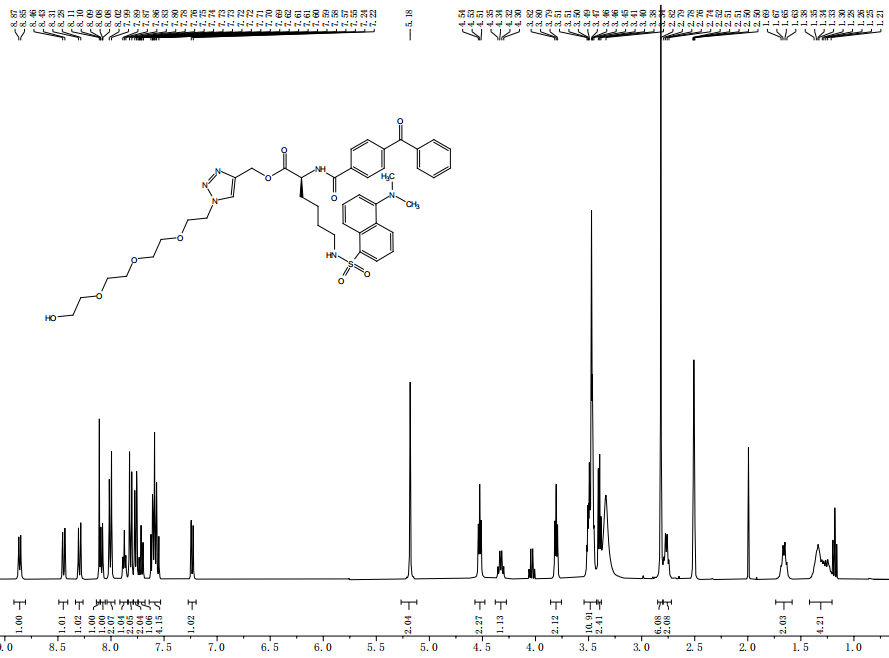


Fig. 34 ^1^H-NMR spectrum of compound **10d**


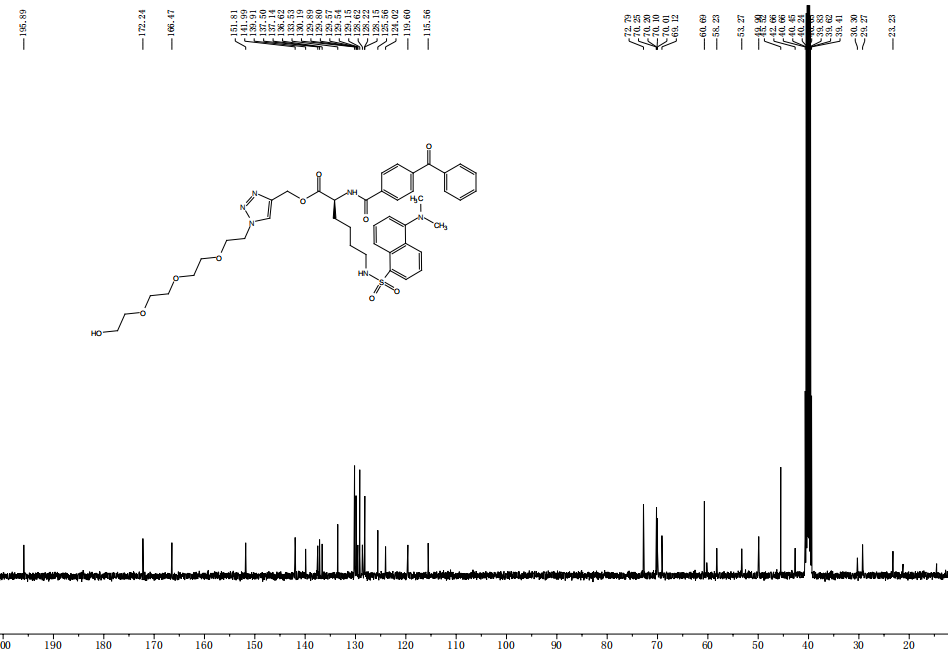


Fig. 35 ^13^C-NMR spectrum of compound **10d**


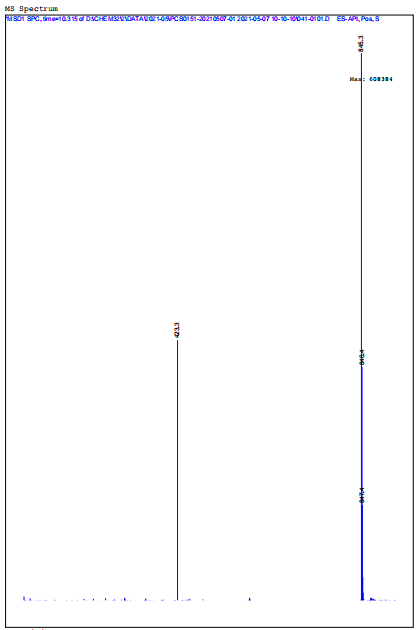


Fig. 36 Low resolution mass spectra of compound **10d**


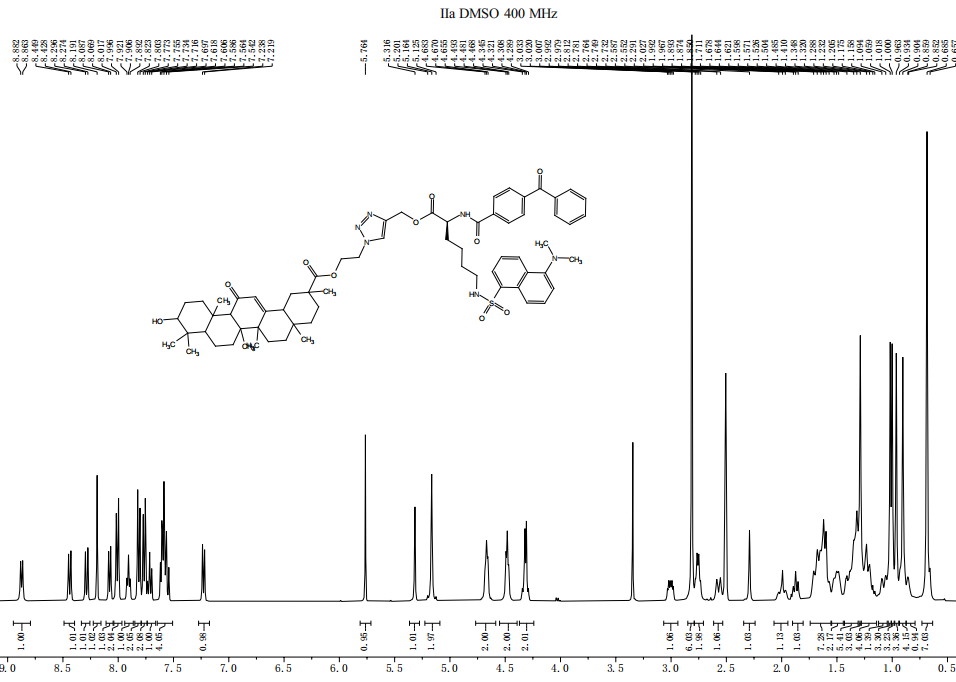


Fig. 37 ^1^H-NMR spectrum of compound **Ⅱa**


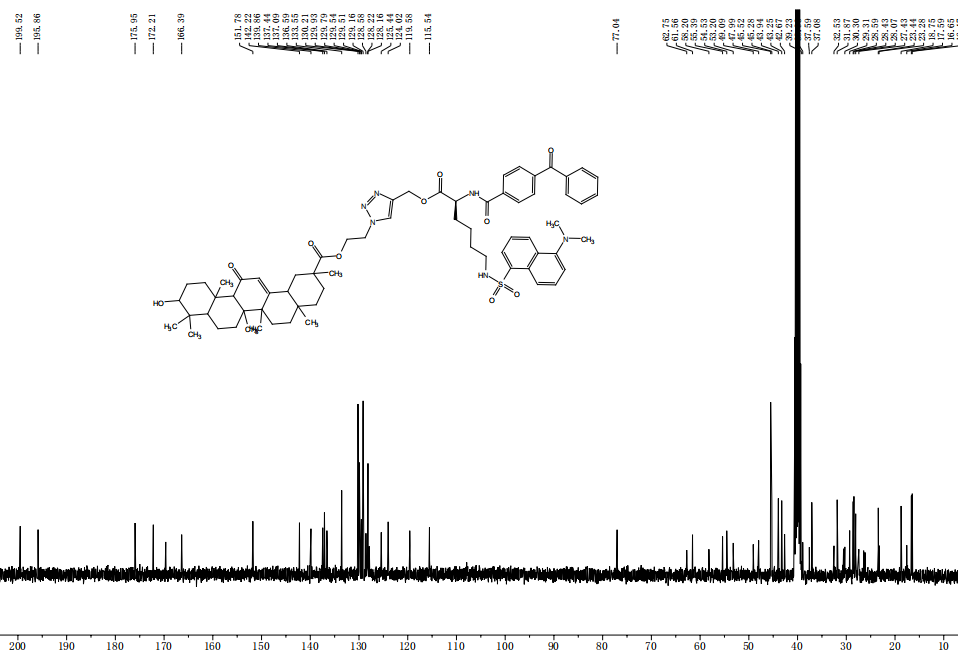


Fig. 38 ^13^C-NMR spectrum of compound **Ⅱa**


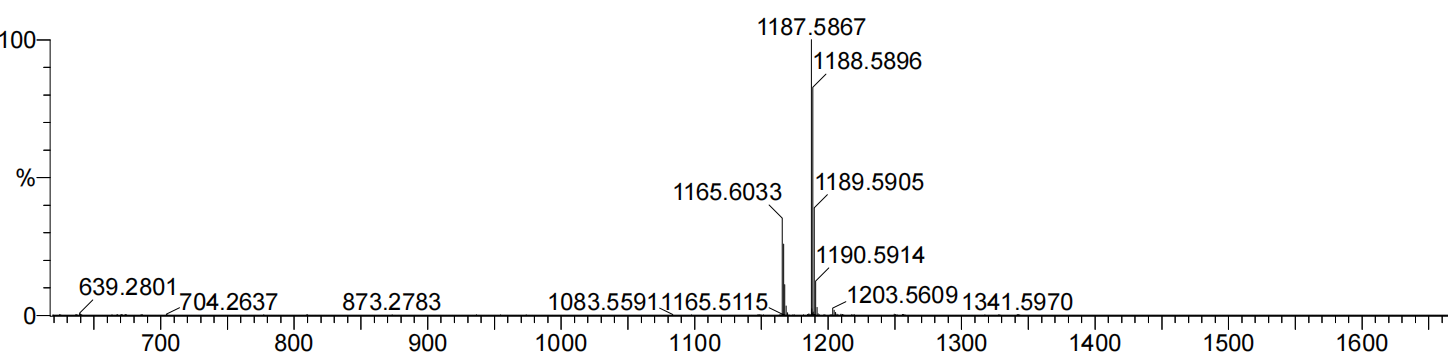


Fig. 39 High resolution mass spectra of compound **Ⅱa**


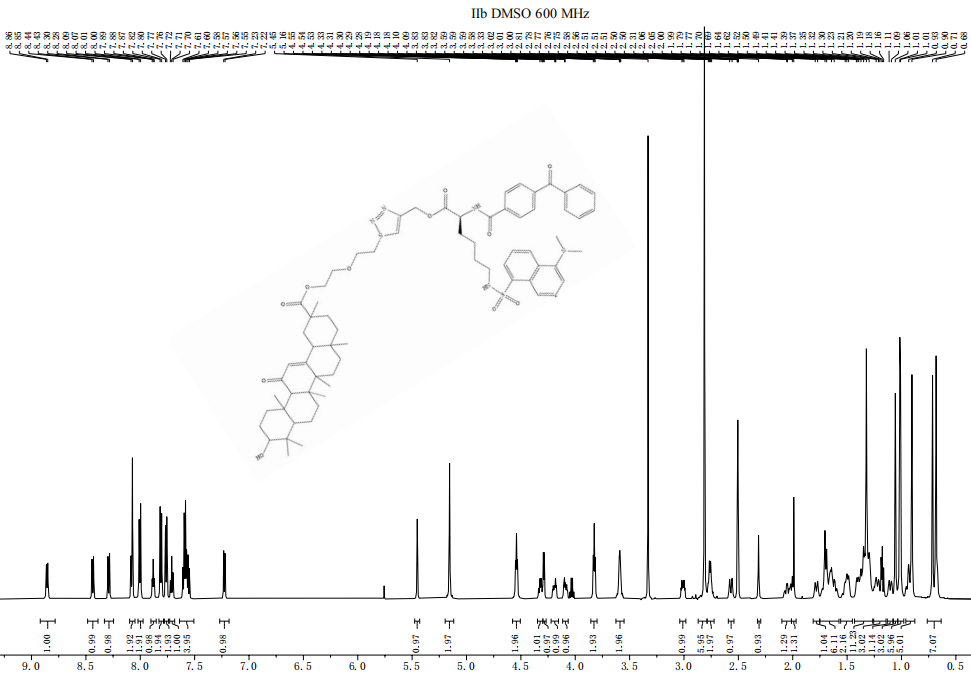


Fig. 40 ^1^H-NMR spectrum of compound **Ⅱb**


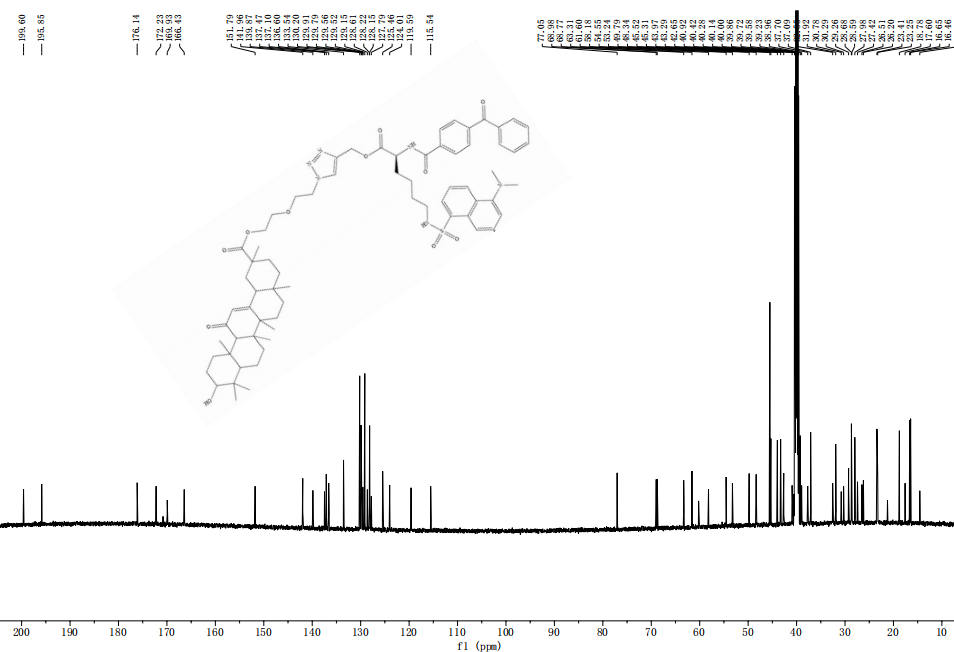


Fig. 41 ^13^C-NMR spectrum of compound **Ⅱb**


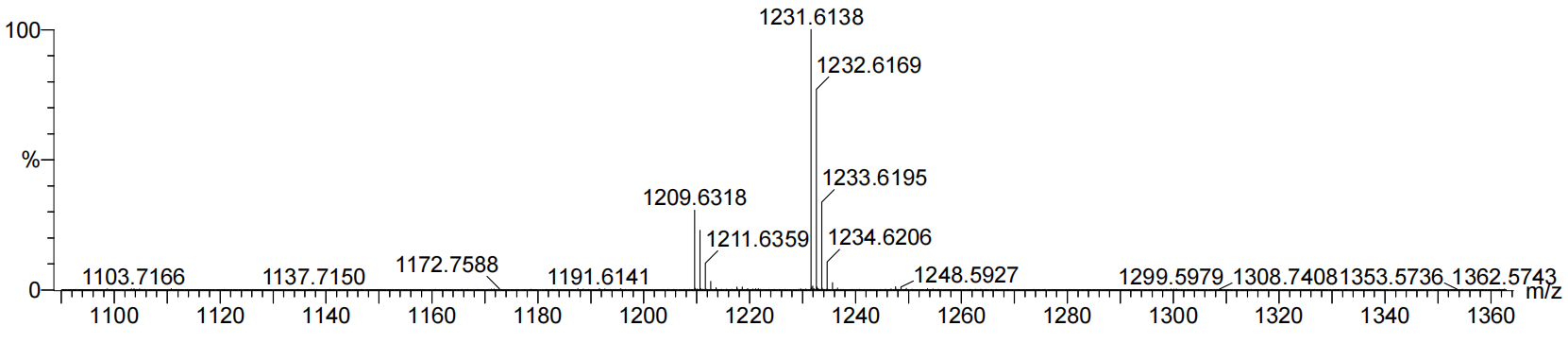


Fig. 42 High resolution mass spectra of compound **Ⅱb**


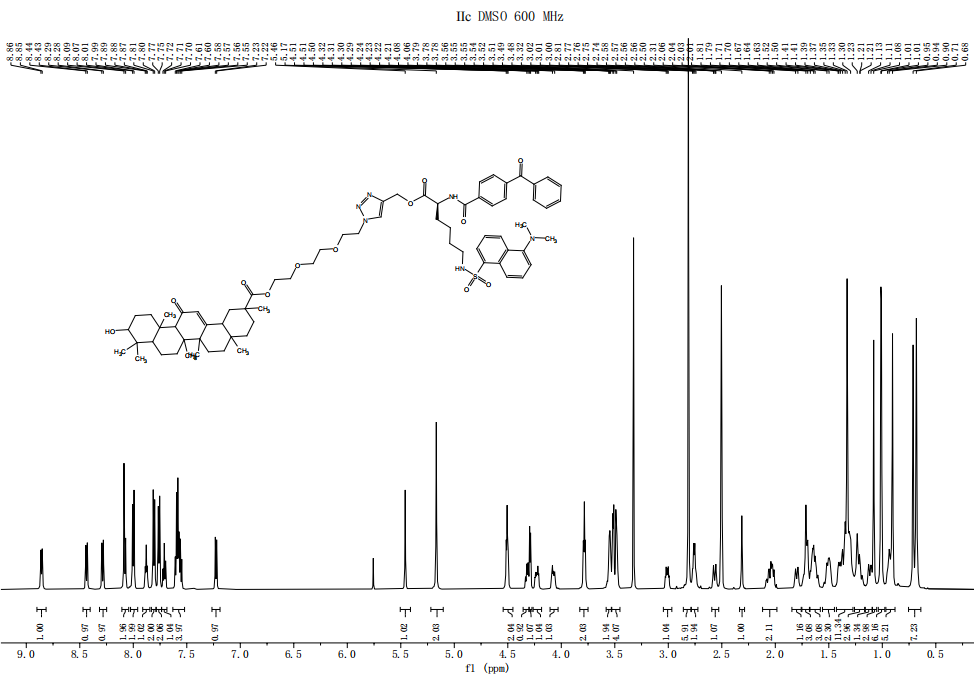


Fig. 43 ^1^H-NMR spectrum of compound **Ⅱc**


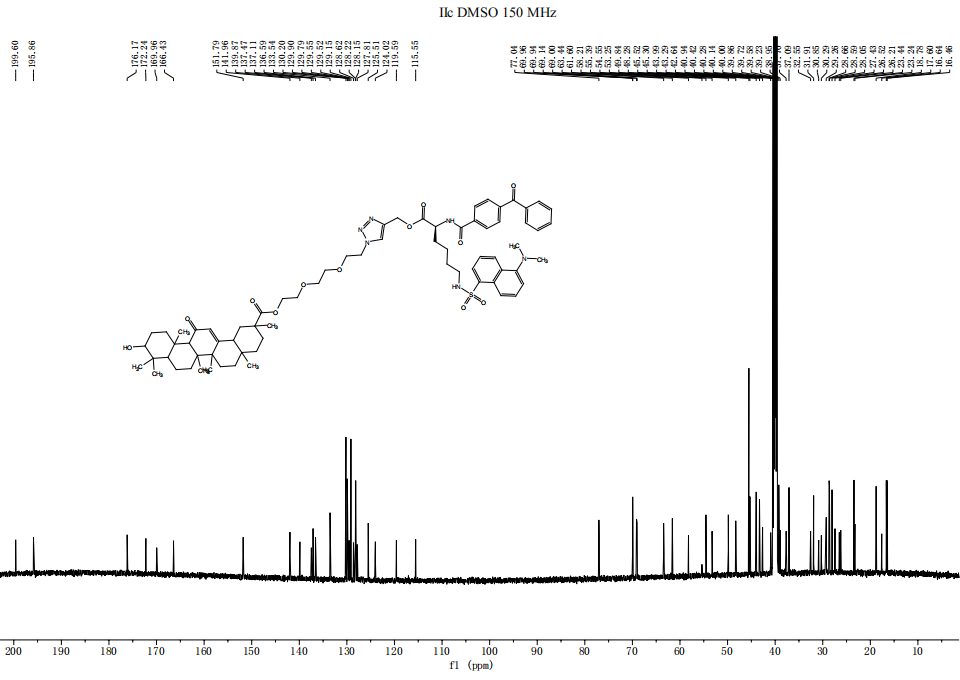


Fig. 44 ^13^C-NMR spectrum of compound **Ⅱc**


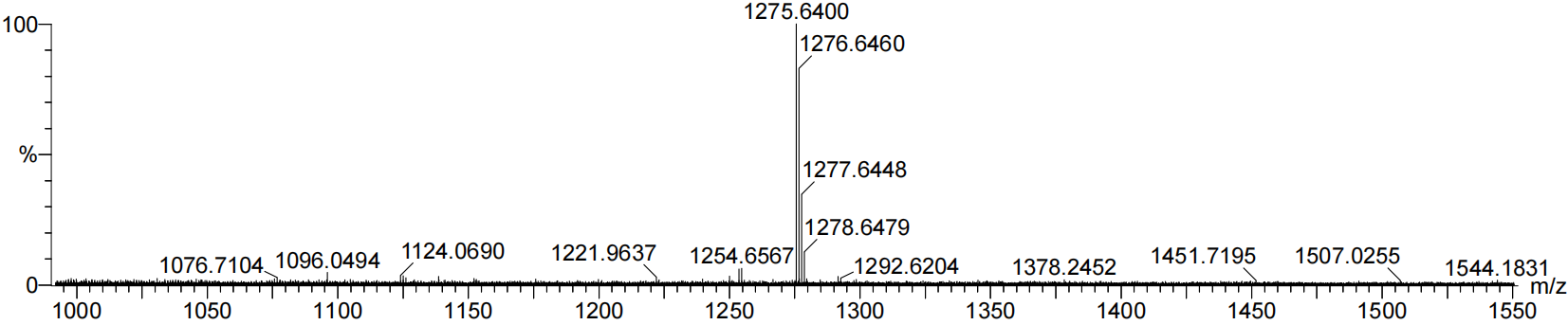


Fig. 45 High resolution mass spectra of compound **Ⅱc**


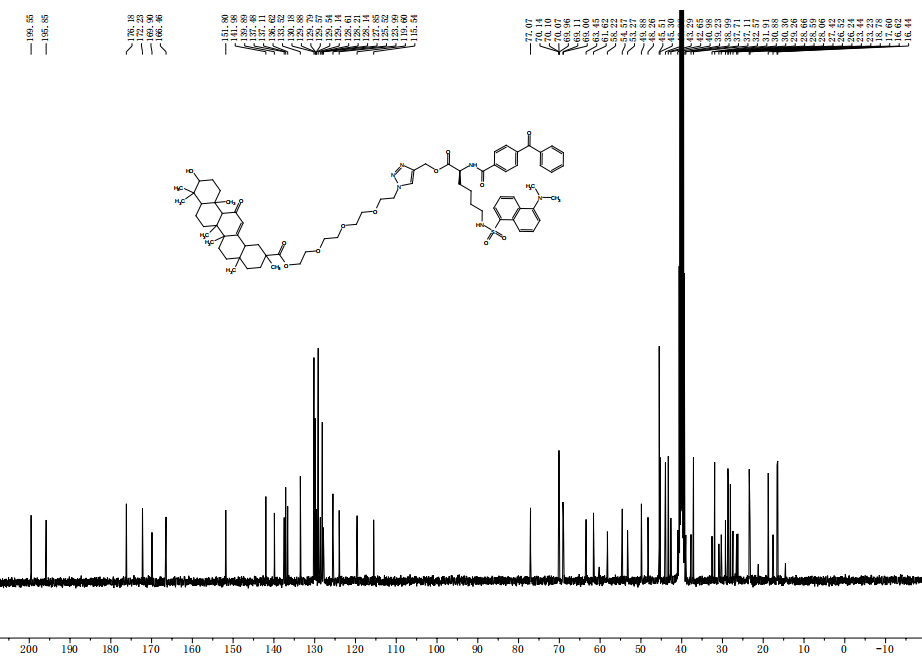


Fig. 46 ^1^H-NMR spectrum of compound **Ⅱd**


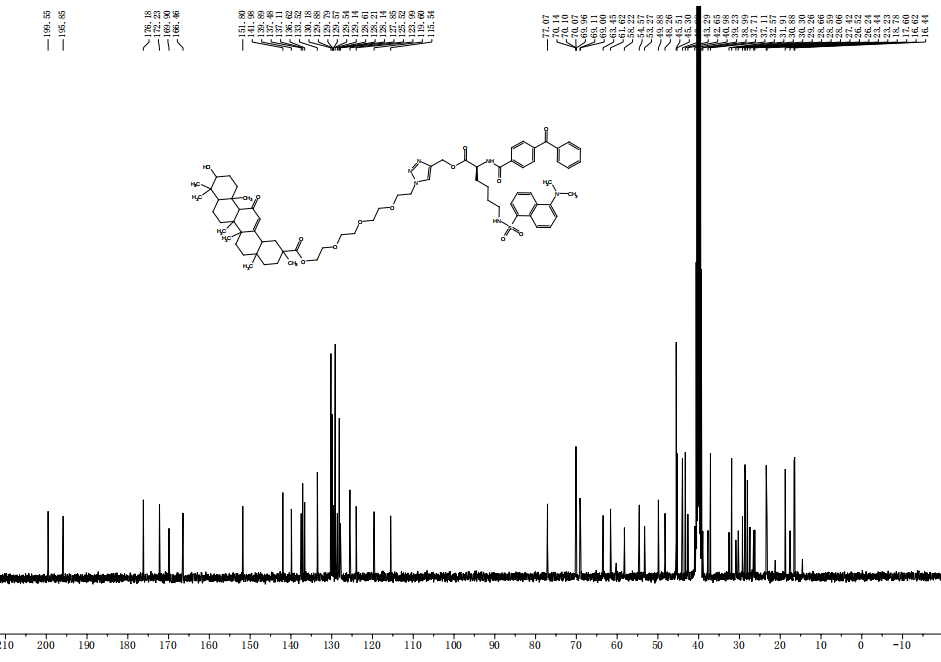


Fig. 47 ^13^C-NMR spectrum of compound **Ⅱd**

Fig. 48 High resolution mass spectra of compound **Ⅱd**

Spectrum of Class Ⅲ compounds:


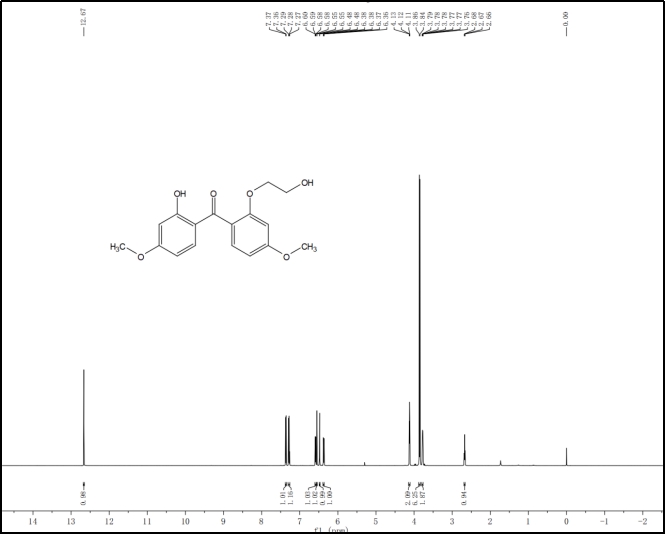


Fig. 49 ^1^H-NMR spectrum of compound **13a**


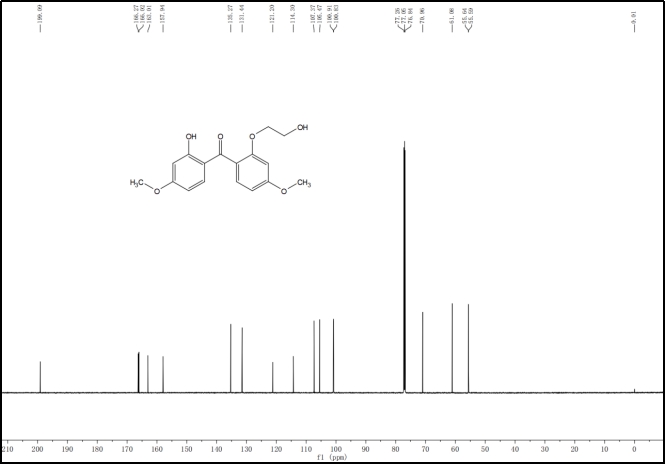


Fig. 50 ^13^C-NMR spectrum of compound **13a**


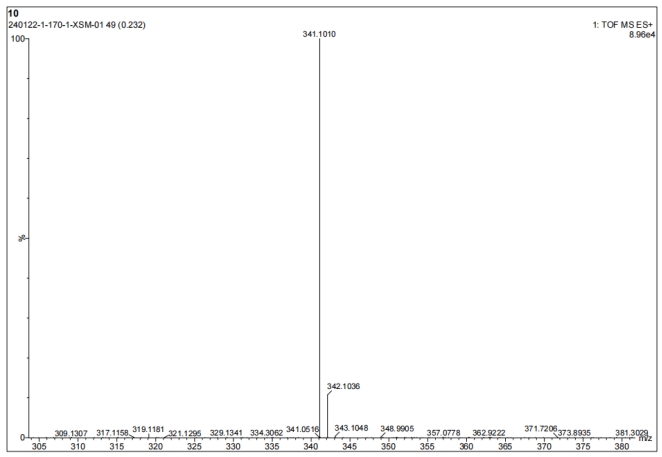


Fig. 51 High resolution mass spectra of compound **13a**


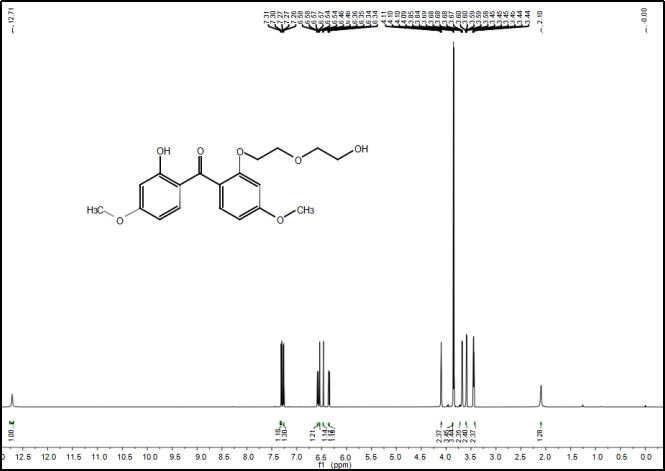


Fig. 52 ^1^H-NMR spectrum of compound **13b**


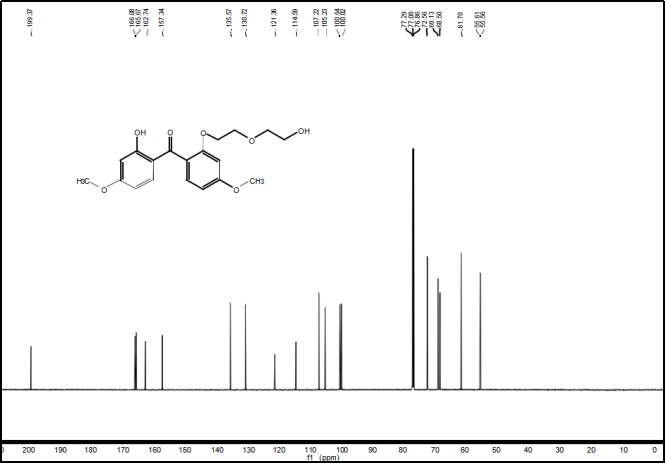


Fig. 53 ^13^C-NMR spectrum of compound **13b**


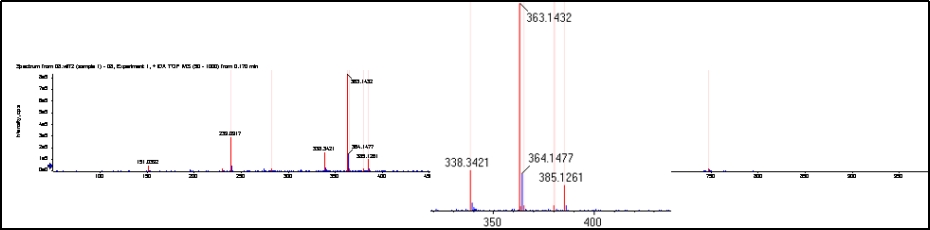


Fig. 54 High resolution mass spectra of compound **13b**
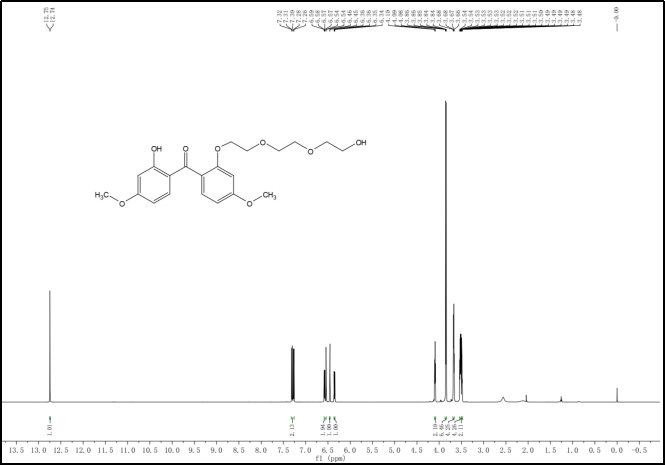


Fig. 55 ^1^H-NMR spectrum of compound **13c**


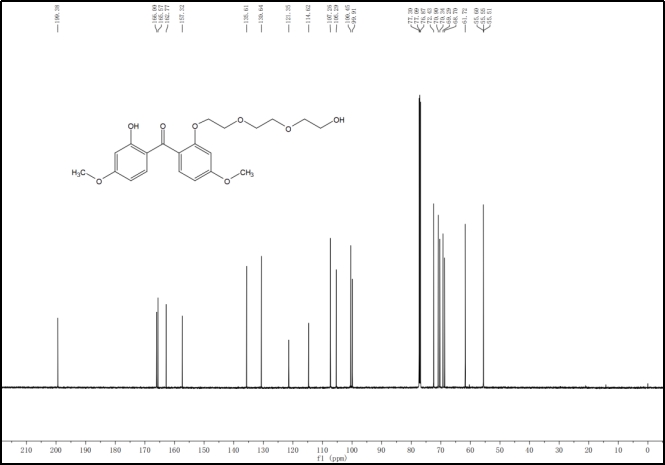


Fig. 56 ^13^C-NMR spectrum of compound **13c**
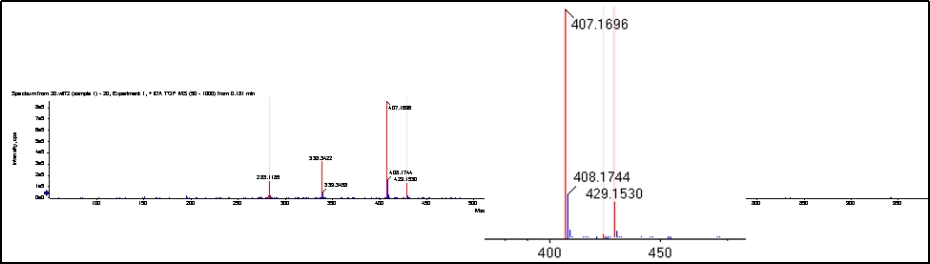


Fig. 57 High resolution mass spectra of compound **13c**
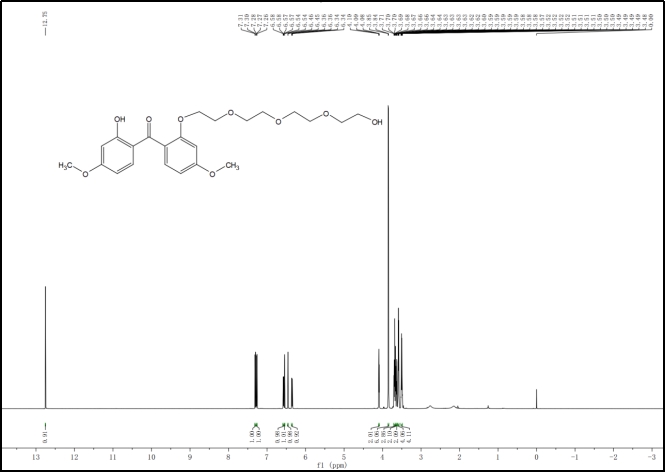


Fig. 58 ^1^H-NMR spectrum of compound **13d**


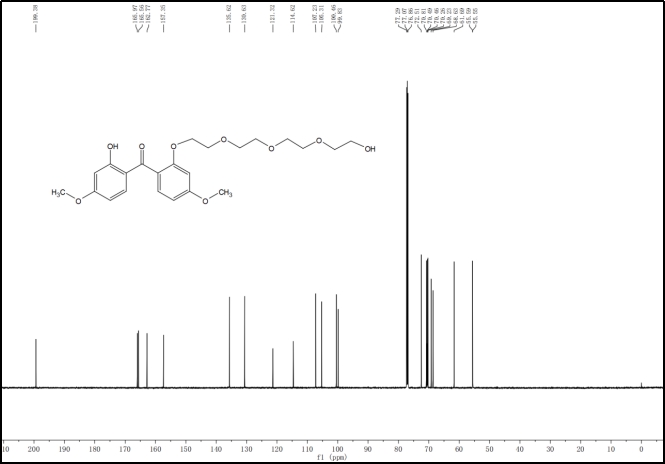


Fig. 59 ^13^C-NMR spectrum of compound **13d**
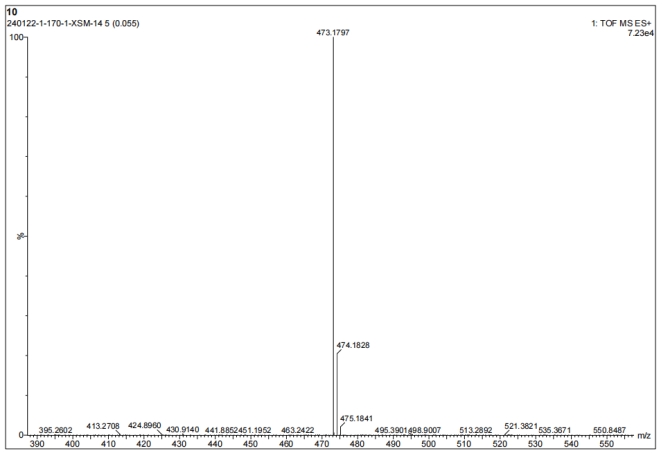


Fig. 60 High resolution mass spectra of compound **13d**
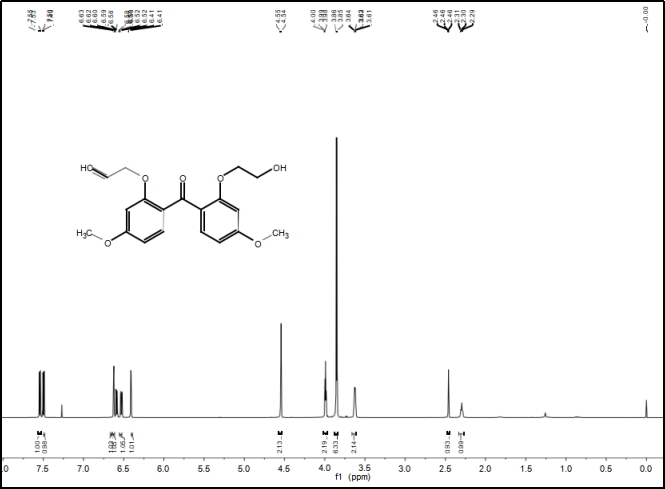


Fig. 61 ^1^H-NMR spectrum of compound **14a**
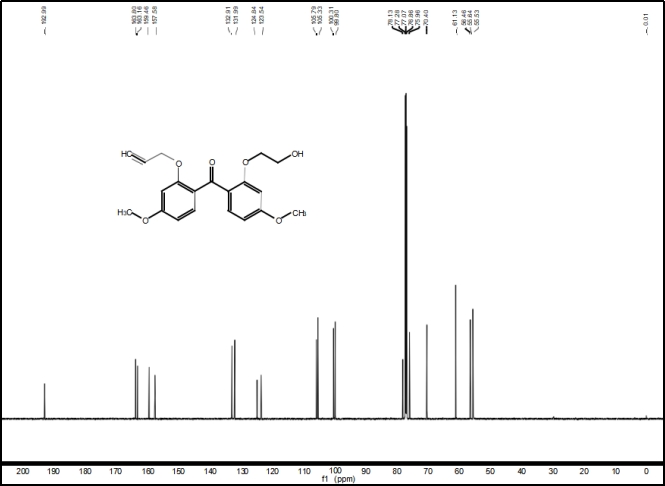


Fig. 62 ^13^C-NMR spectrum of compound **14a**
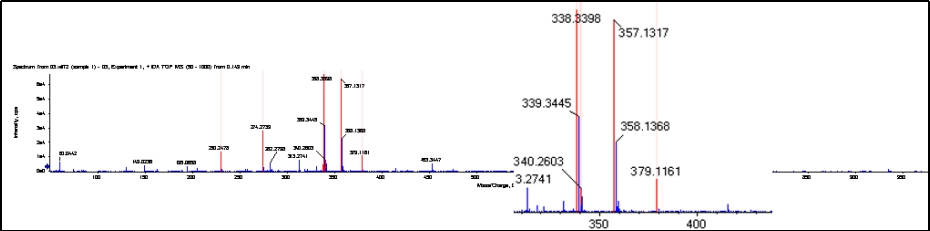


Fig. 63 High resolution mass spectra of compound **14a**


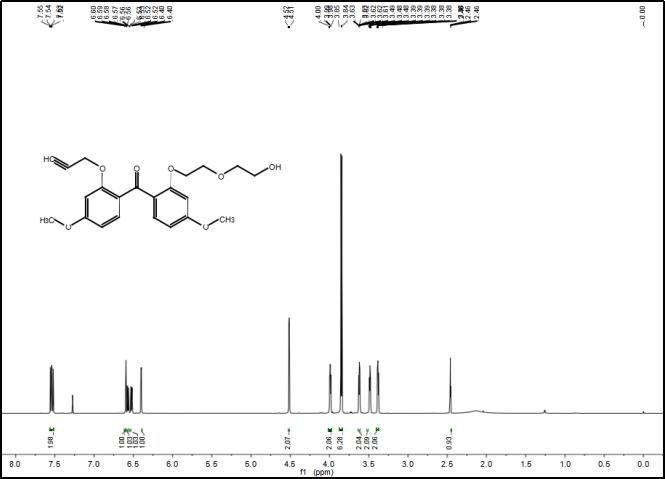


Fig. 64 ^1^H-NMR spectrum of compound **14b**


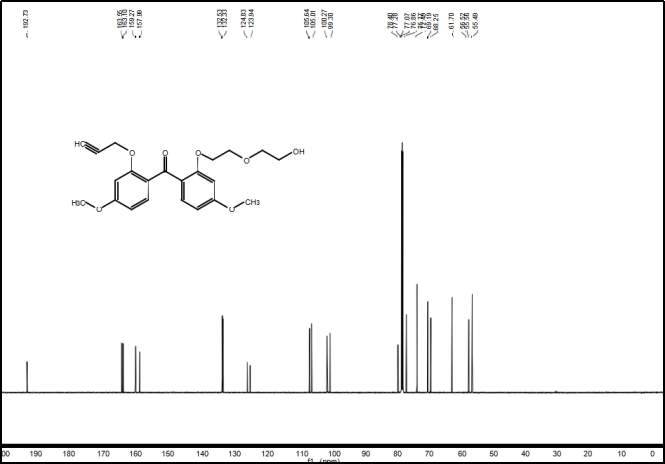


Fig. 65 ^13^C-NMR spectrum of compound **14b**


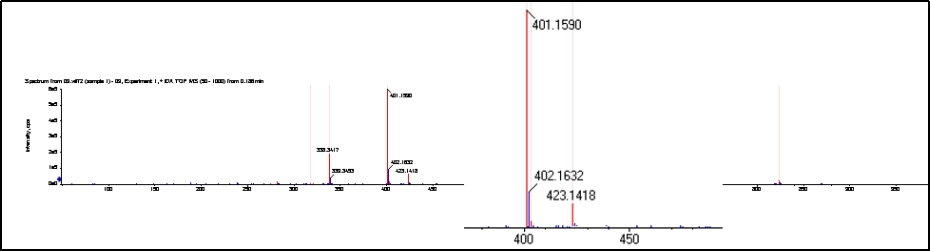


Fig. 66 High resolution mass spectra of compound **14b**


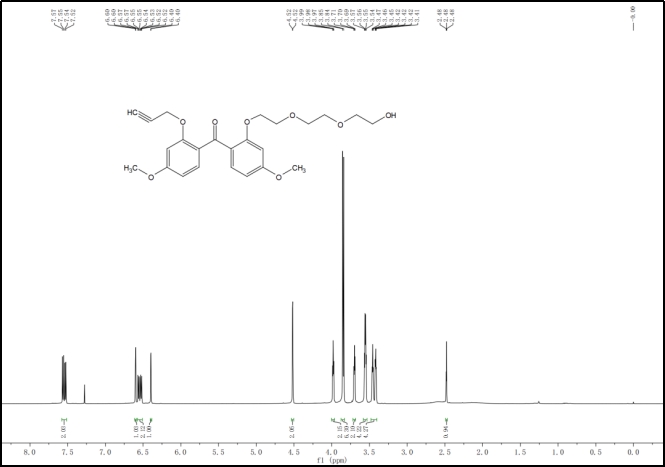


Fig. 67 ^1^H-NMR spectrum of compound **14c**


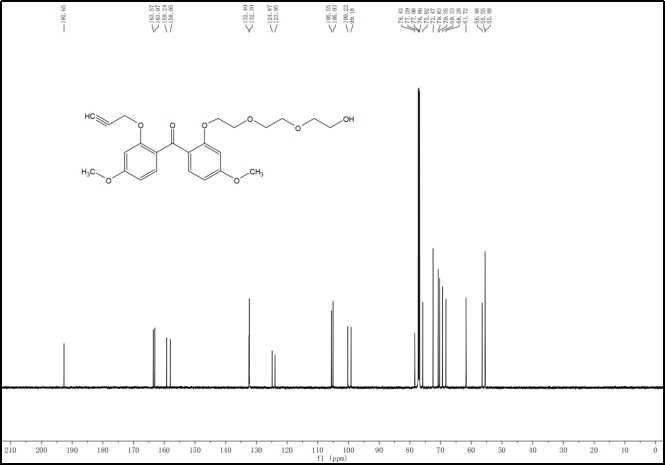


Fig. 68 ^13^C-NMR spectrum of compound **14c**


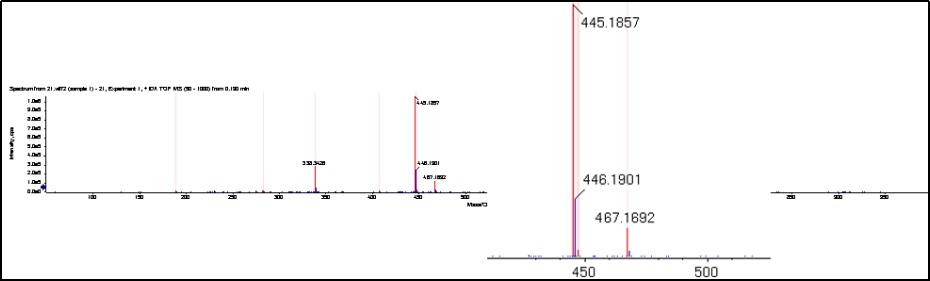


Fig. 69 High resolution mass spectra of compound **14c**


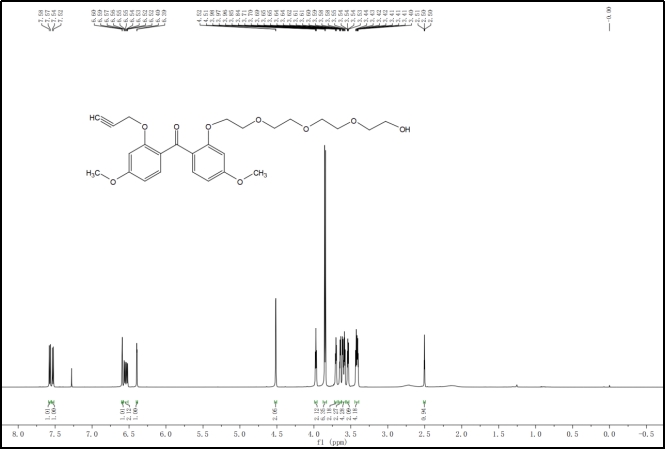


Fig. 70 ^1^H-NMR spectrum of compound **14d**


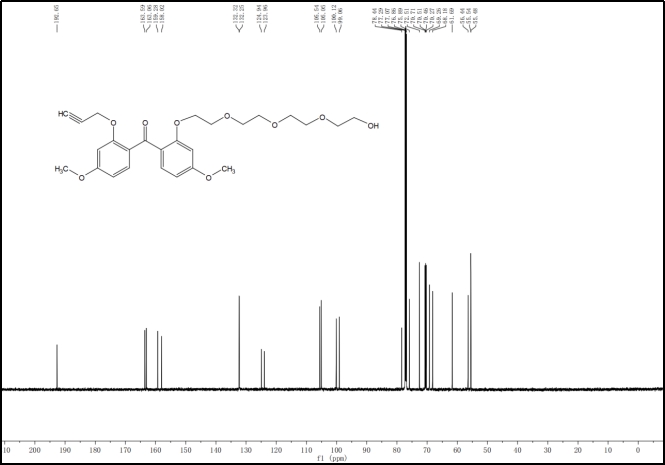


Fig. 71 ^13^C-NMR spectrum of compound **14d**


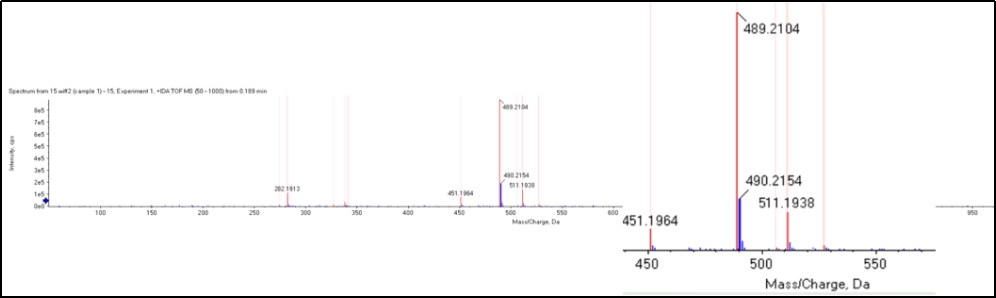


Fig. 72 High resolution mass spectra of compound **14d**


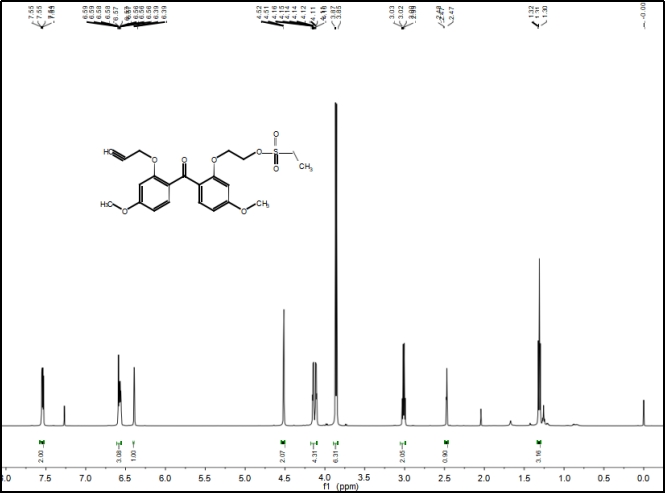


Fig. 73 ^1^H-NMR spectrum of compound **15a**


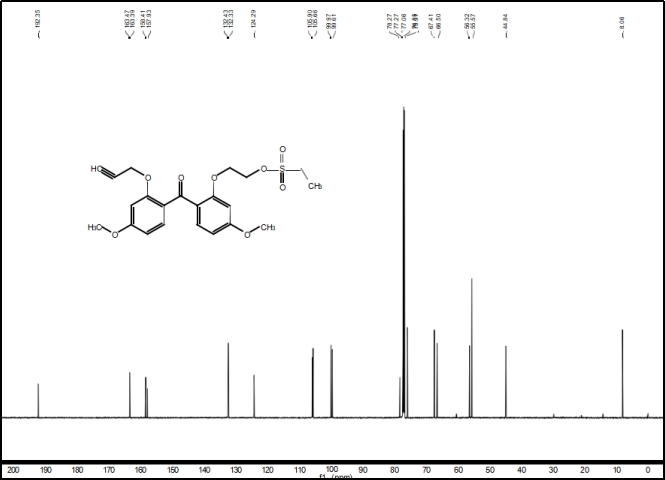


Fig. 74 ^13^C-NMR spectrum of compound **15a**


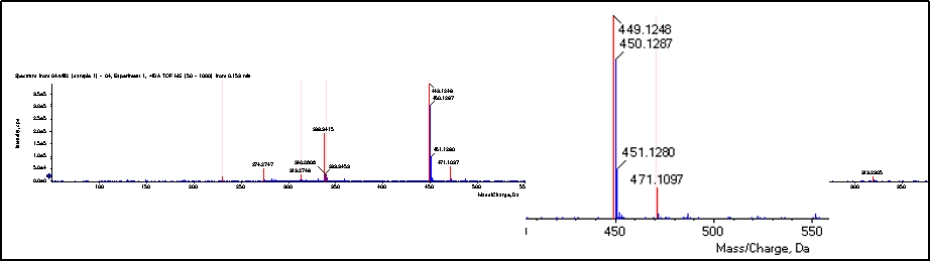


Fig. 75 High resolution mass spectra of compound **15a**


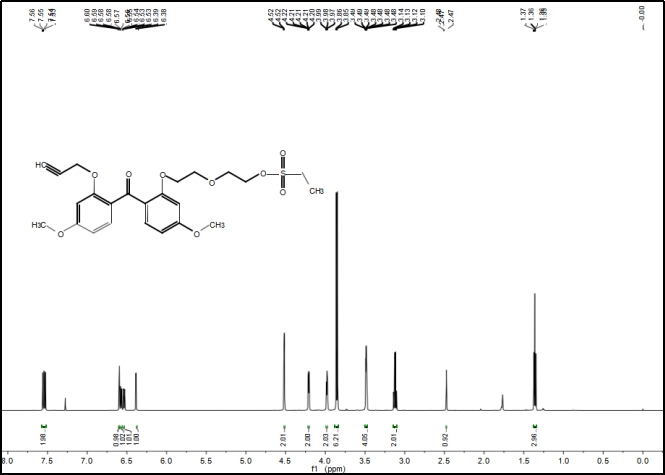


Fig. 76 ^1^H-NMR spectrum of compound **15b**


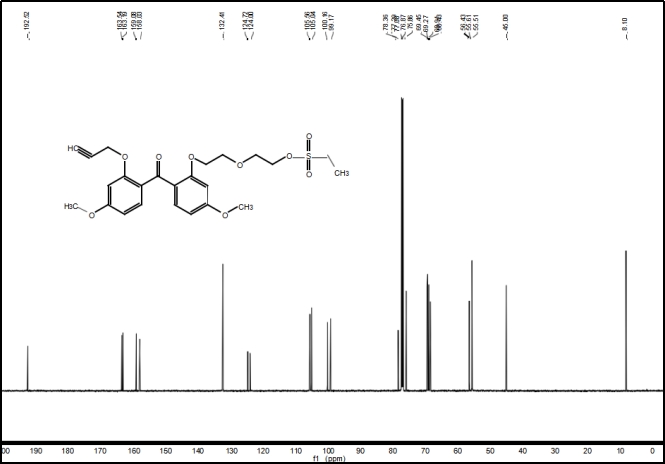


Fig. 77 ^13^C-NMR spectrum of compound **15b**


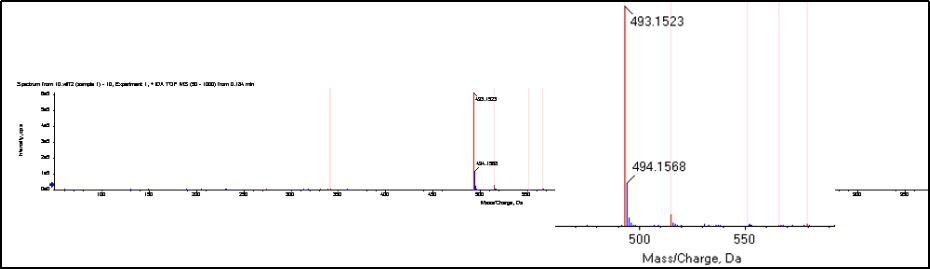


Fig. 78 High resolution mass spectra of compound **15b**


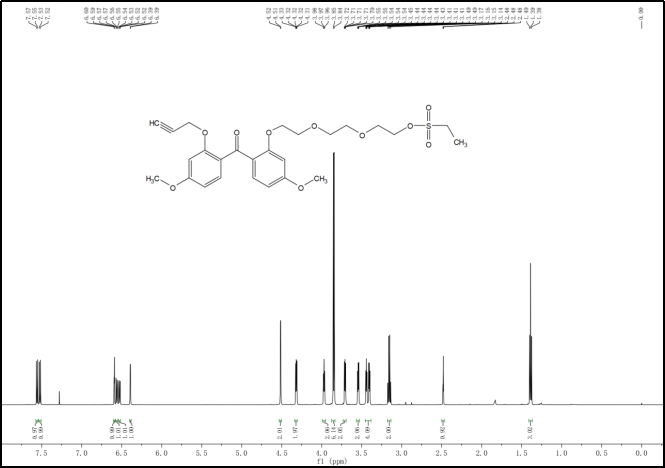


Fig. 79 ^1^H-NMR spectrum of compound **15c**


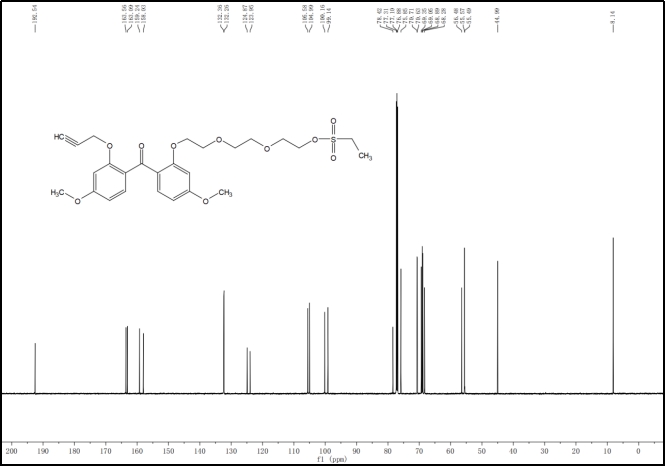


Fig. 80 ^13^C-NMR spectrum of compound **15c**


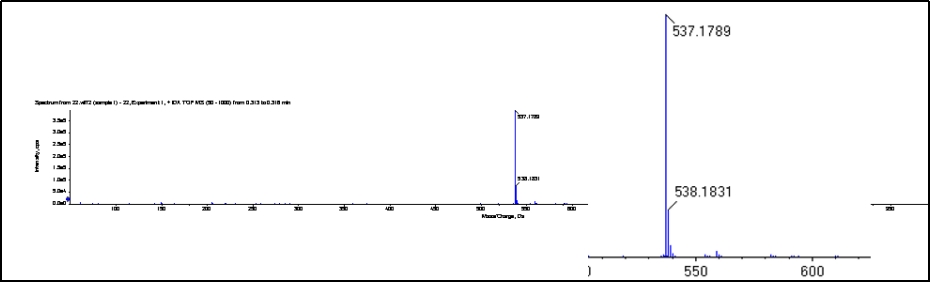


Fig. 81 High resolution mass spectra of compound **15c**


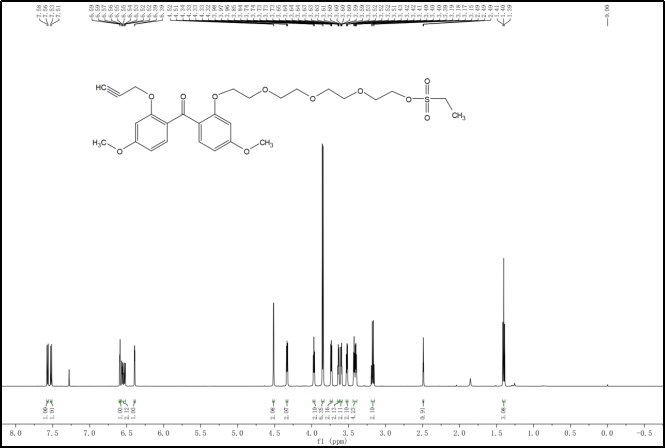


Fig. 82 ^1^H-NMR spectrum of compound **15d**


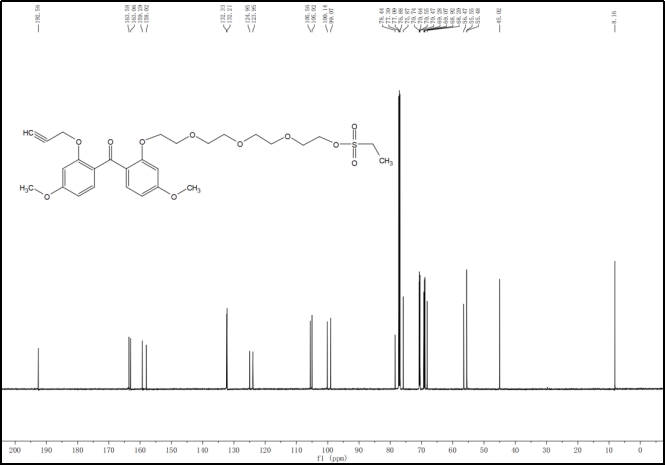


Fig. 83 ^13^C-NMR spectrum of compound **15d**


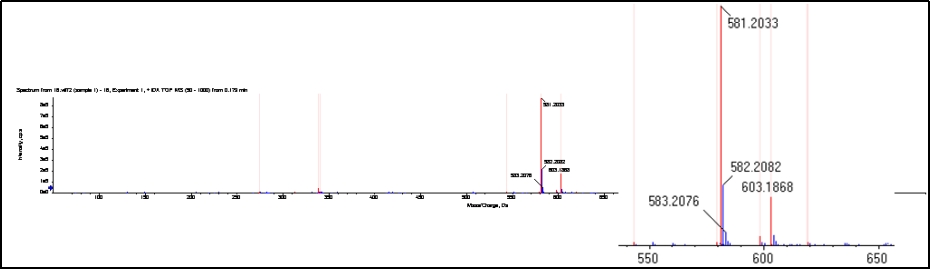


Fig. 84 High resolution mass spectra of compound **15d**


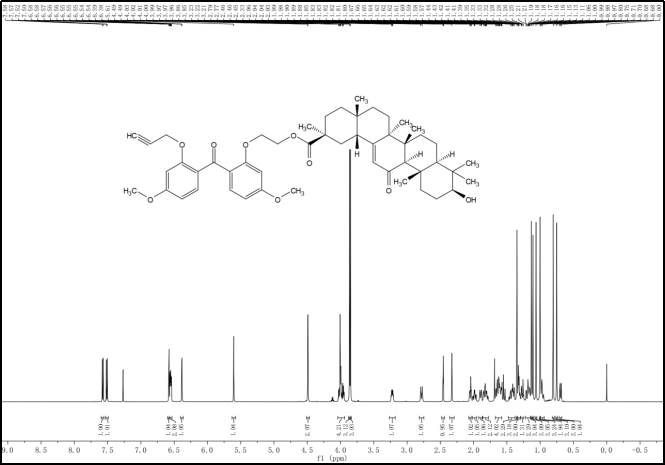


Fig. 85 ^1^H-NMR spectrum of compound **16a**


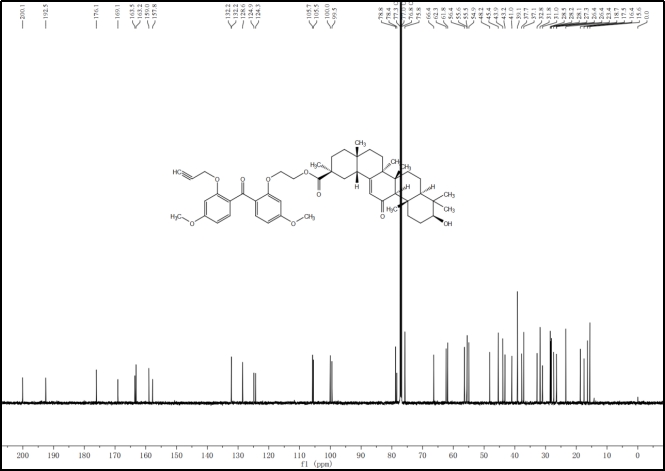


Fig. 86 ^13^C-NMR spectrum of compound **16a**


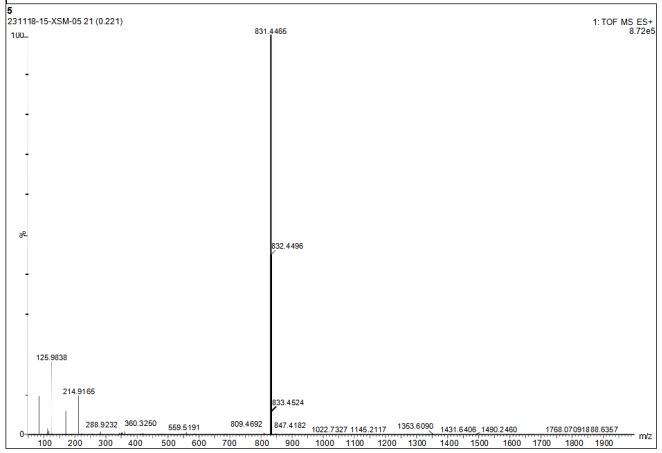


Fig. 87 High resolution mass spectra of compound **16a**


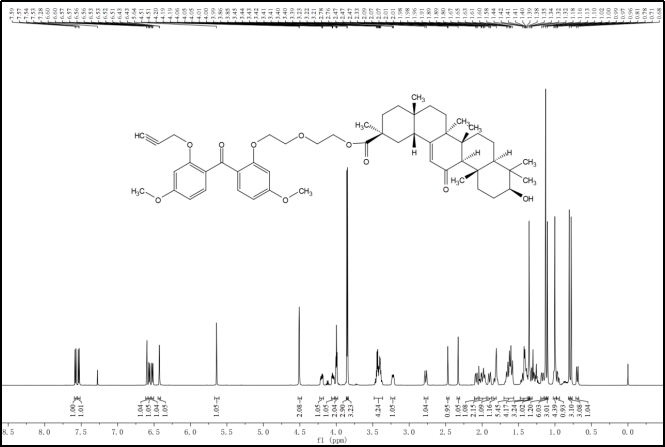


Fig. 88 ^1^H-NMR spectrum of compound **16b**


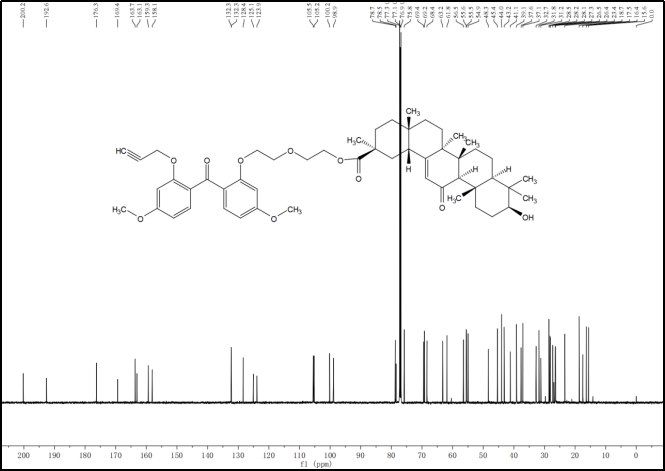


Fig. 89 ^13^C-NMR spectrum of compound **16b**


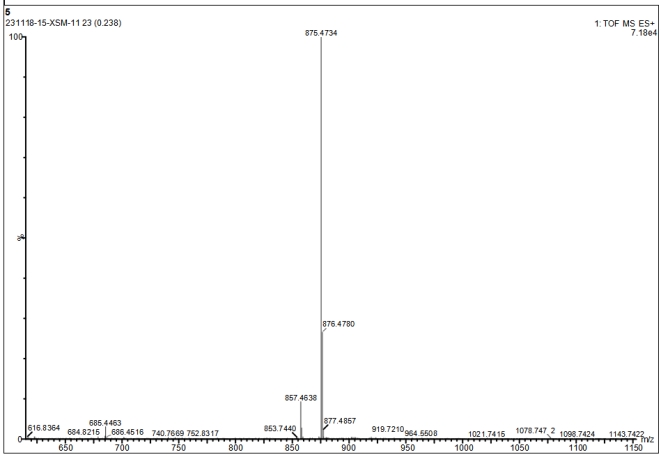


Fig. 90 High resolution mass spectra of compound **16b**


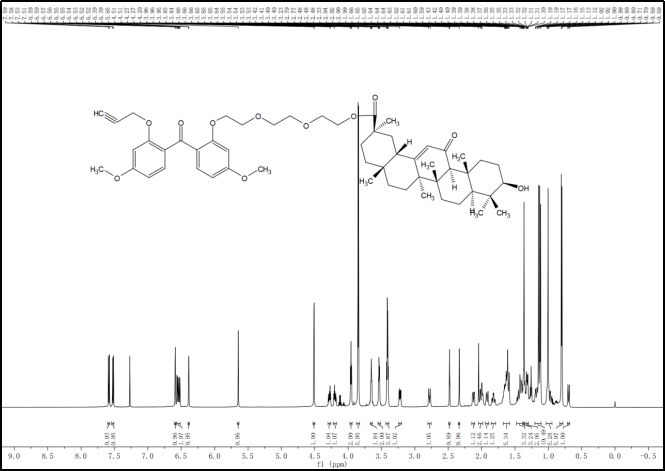


Fig. 91 ^1^H-NMR spectrum of compound **16c**


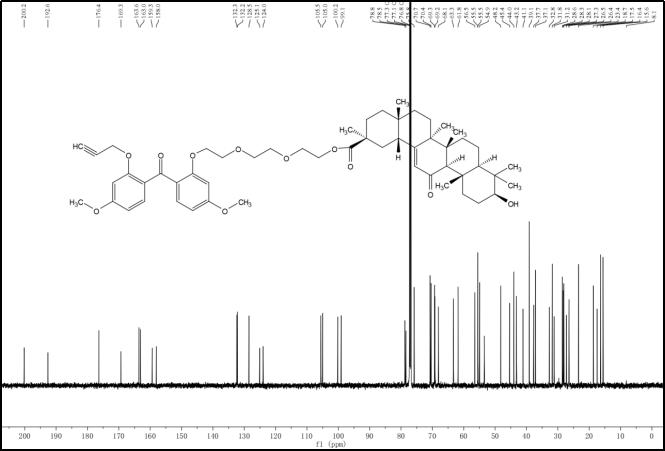


Fig. 92 ^13^C-NMR spectrum of compound **16c**


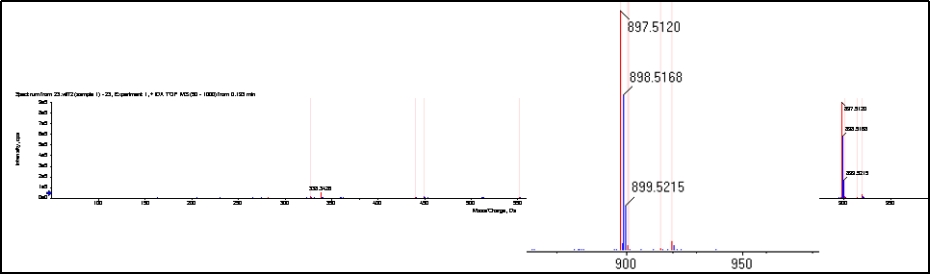


Fig. 93 High resolution mass spectra of compound **16c**


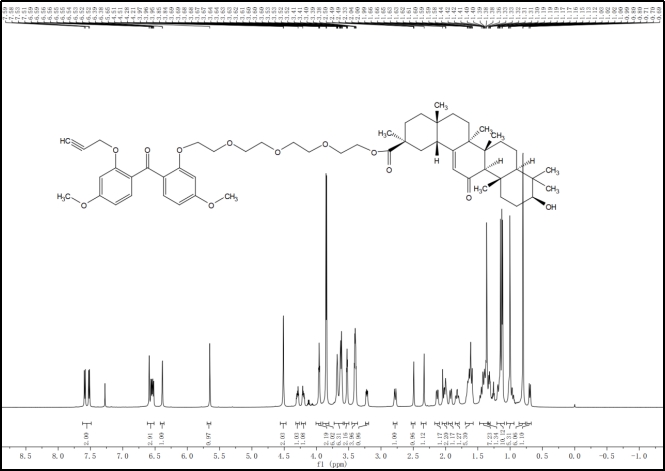


Fig. 94 ^1^H-NMR spectrum of compound **16d**


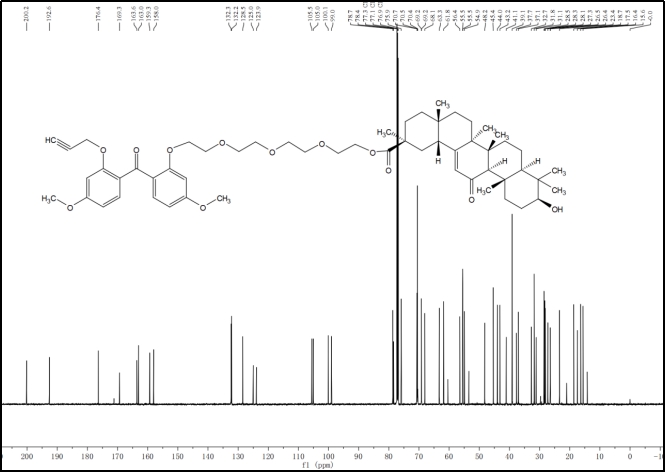


Fig. 95 ^13^C-NMR spectrum of compound **16d**


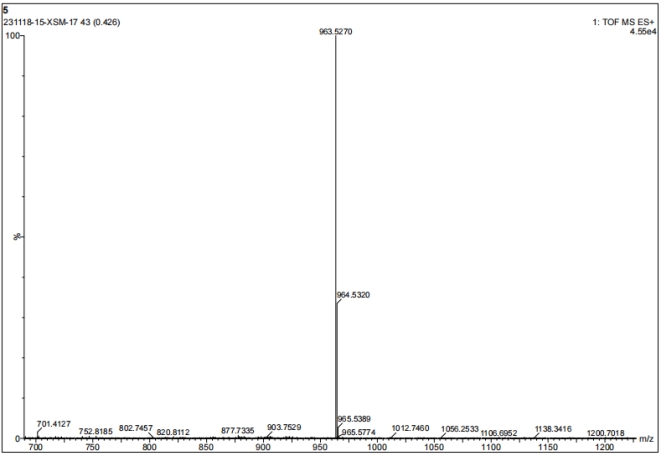


Fig. 96 High resolution mass spectra of compound **16d**


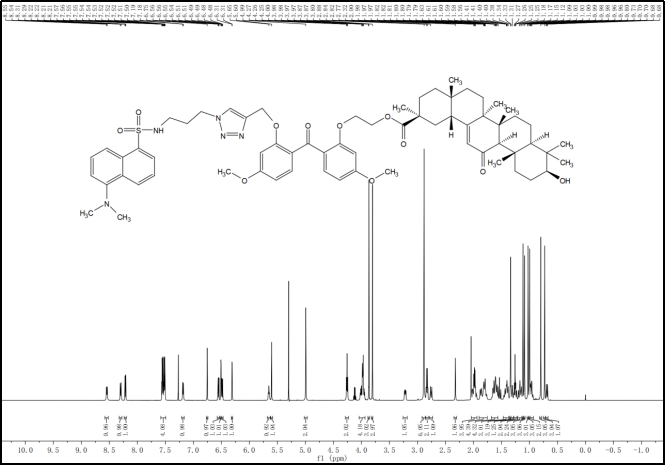


Fig. 97 ^1^H-NMR spectrum of compound **Ⅲa**


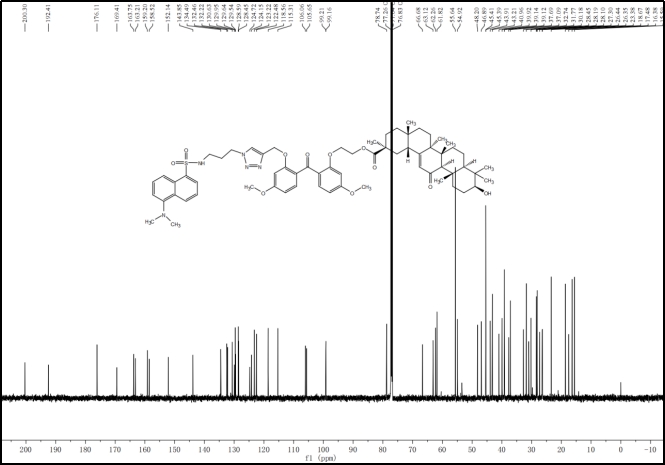


Fig. 98 ^13^C-NMR spectrum of compound **Ⅲa**
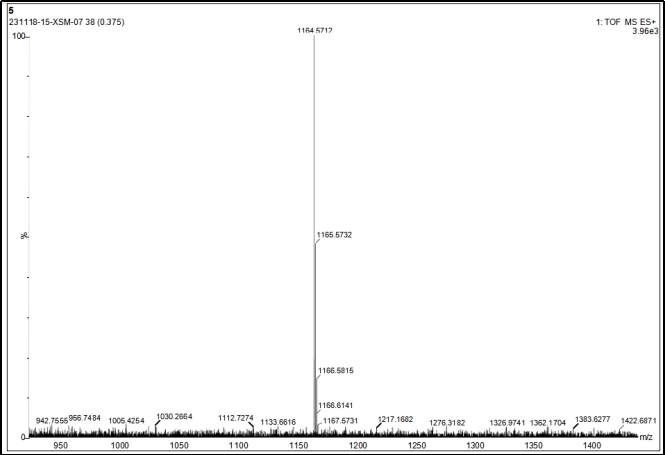


Fig. 99 High resolution mass spectra of compound **Ⅲa**


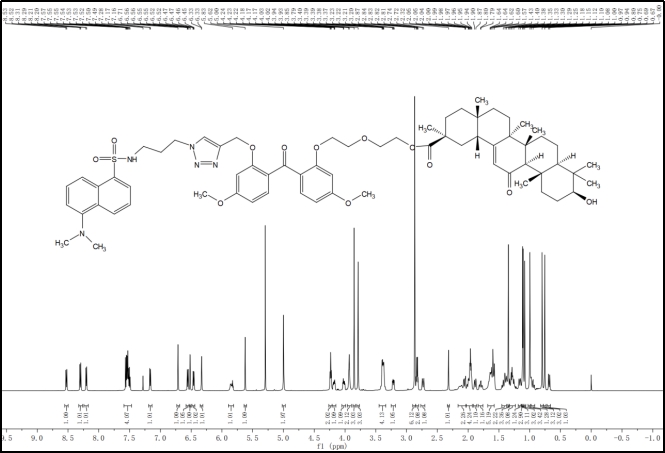


Fig. 100 ^1^H-NMR spectrum of compound **Ⅲb**


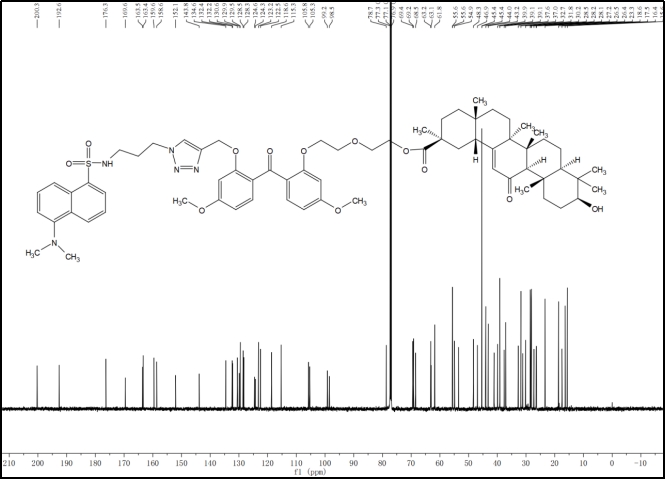


Fig. 101 ^13^C-NMR spectrum of compound **Ⅲb**


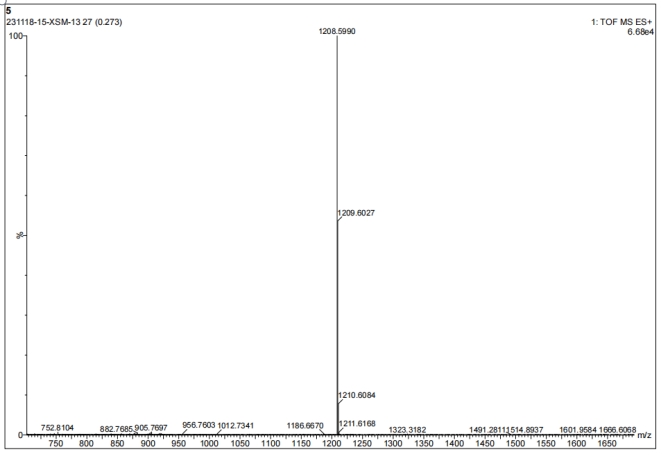


Fig. 102 High resolution mass spectra of compound **Ⅲb**


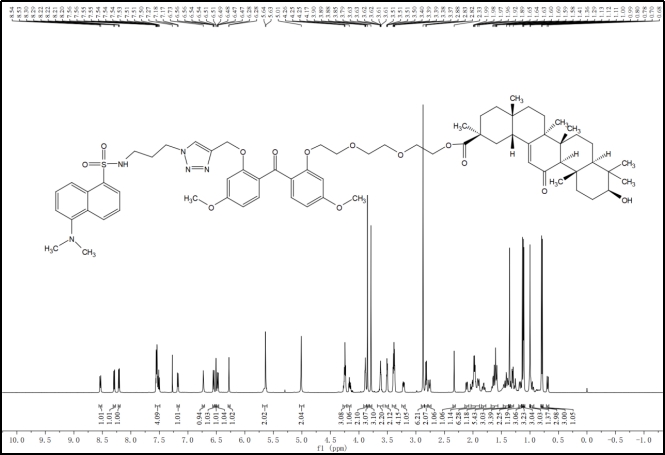


Fig. 103 ^1^H-NMR spectrum of compound **Ⅲc**

Fig. 104 ^13^C-NMR spectrum of compound **Ⅲc**

Fig. 105 High resolution mass spectra of compound **Ⅲc**

Fig. 106 ^1^H-NMR spectrum of compound **Ⅲd**

Fig. 107 ^13^C-NMR spectrum of compound **Ⅲd**

Fig. 108 High resolution mass spectra of compound **Ⅲd**
